# Human milk nutritional composition across lactational stages in Central Africa

**DOI:** 10.3389/fnut.2022.1033005

**Published:** 2022-11-16

**Authors:** Violeta Moya-Alvarez, Simone R. B. M. Eussen, Marko Mank, Jean-Christophe Junior Koyembi, Yawo Tufa Nyasenu, Gilles Ngaya, Daniel Mad-Bondo, Jean-Bertrand Kongoma, Bernd Stahl, Philippe J. Sansonetti, Raphaëlle Bourdet-Sicard

**Affiliations:** ^1^Unité de Pathogénie Microbienne Moléculaire, INSERM U1202, Department of Cell Biology and Infection, Institut Pasteur, Paris, France; ^2^Epidemiology of Emergent Diseases Unit, Global Health Department, Institut Pasteur, Paris, France; ^3^Human Milk Research and Analytical Science, Danone Nutricia Research, Utrecht, Netherlands; ^4^Unité d'Epidémiologie, Institut Pasteur de Bangui, Bangui, Central African Republic; ^5^Laboratoire d'Analyses Médicales, Institut Pasteur de Bangui, Bangui, Central African Republic; ^6^Laboratoire de Biologie Moléculaire et d'Immunologie, Université de Lomé, Lomé, Togo; ^7^Direction du Service de Santé de la Gendarmerie, Sis Camp Henri Izamo, Bangui, Central African Republic; ^8^Department of Chemical Biology and Drug Discovery, Utrecht Institute for Pharmaceutical Sciences, Utrecht University, Utrecht, Netherlands; ^9^Chaire de Microbiologie et Maladies Infectieuses, Collège de France, Paris, France; ^10^Health and Nutrition Africa, Danone Nutricia Research, Palaiseau, France

**Keywords:** human milk, human milk oligosaccharides (HMOs), Africa, fatty acids, amino acids

## Abstract

The African region encompasses the highest undernutrition burden with the highest neonatal and infant mortality rates globally. Under these circumstances, breastfeeding is one of the most effective ways to ensure child health and development. However, evidence on human milk (HM) composition from African women is scarce. This is of special concern, as we have no reference data from HM composition in the context of food insecurity in Africa. Furthermore, data on the evolution of HM across lactational stages in this setting lack as well. In the MITICA study, we conducted a cohort study among 48 Central-African women and their 50 infants to analyze the emergence of gut dysbiosis in infants and describe the mother-infant transmission of microbiota between birth and 6 months of age. In this context, we assessed nutritional components in HM of 48 lactating women in Central Africa through five sampling times from week 1 after birth until week 25. Unexpectedly, HM-type III (Secretor + and Lewis genes -) was predominant in HM from Central African women, and some nutrients differed significantly among HM-types. While lactose concentration increased across lactation periods, fatty acid concentration did not vary significantly. The overall median level of 16 detected individual human milk oligosaccharides (HMOs; core structures as well as fucosylated and sialylated ones) decreased from 7.3 g/l at week 1 to 3.5 g/l at week 25. The median levels of total amino acids in HM dropped from 12.8 mg/ml at week 1 to 7.4 mg/ml at week 25. In contrast, specific free amino acids increased between months 1 and 3 of lactation, e.g., free glutamic acid, glutamine, aspartic acid, and serine. In conclusion, HM-type distribution and certain nutrients differed from Western mother HM.

## Introduction

The oneiric image of Hera's spilled breastmilk forming the path of stars leading to Olympus, the Milky Way, reflects the importance of breastmilk for humanity since the origin of humankind. Breastfeeding gives every newborn the best possible welcoming in life. It nourishes him or her, protects from infections, and provides emotional benefits to both newborns and mothers. Indeed, human milk (HM) is the gold-standard nutrient source for newborns, providing a tailored plethora of nutrients, bioactive compounds, and immunologically active molecules essential for optimal development of infants (1, 2). Early initiation of breastfeeding, within 1 h after birth, protects the newborn from acquiring infections and reduces the risk of neonatal mortality (3). Human milk provides all the energy and nutrients that the infant needs for the first 6 months of life, and it continues to provide up to half or more of a child's necessary nutrients during the rest of the 1st year, and up to one third during the 2nd year of life (3). Stunting rates and child mortality due to diarrhea are reduced in exclusively breastfed infants, and children and adolescents who were breastfed are less likely to be overweight or obese (1, 2). Also, they perform better on intelligence tests and have higher school attendance. Additionally, breastfeeding plays a notable role in birth-spacing (2), which is especially important in low-income countries.

The African region encompasses the highest undernutrition burden with the highest neonatal and infant mortality rates globally (4). In 2020, 30.7% of children under 5 years were stunted (5), and undernutrition was associated with 2.7 million child deaths globally (45% of all child deaths) (1). Only 45.7% of African infants 0–6 months old are exclusively breastfed according to World Health Organization (WHO) estimates (1). Breastfeeding is essential to ensure child health and survival in this region. Estimates presume that optimal breastfeeding between 0 and 23 months could save over 820,000 children's lives annually (2). Therefore, WHO recommends exclusive breastfeeding during the first 6 months of life worldwide and continued breastfeeding along with complementary foods up to 2 years and beyond.

Recent research from middle- and high-income countries has stressed the importance of HM composition to respond to the energetic demands and immunological development of the newborn (6–8). The HM fat fraction constitutes the main source (44% on average) of energy supplies during infancy and its concentration depends on maternal diet, lactation period and other miscellaneous factors (9). Human milk proteins, peptides and free amino acids (AAs) support adequate growth and immune functions (10–12). Human milk oligosaccharides (HMOs) are effective prebiotics with metabolic, anti-infective, and immunomodulatory properties (13, 14). They may also assist in healthy development of the gut microbiome and gut maturation (13, 15–19). HM is proposed to comprise more than 1,000 individual and unique HMOs of which ~>160 are fully structurally characterized (20). Depending on maternal Secretor (Se) and Lewis (Le) genes (21, 22), four different milk groups or milk types can be distinguished in human milk specimens. Lactating women with Se+ and Le+, Se- and Le+, Se+ and Le-, or Se- and Le- genotype produce HM-type I, HM-type II, HM-type III, and HM-type IV, respectively. HM-type distribution may vary in different geographies. For example, HM type I is predominant in Europe (21, 22).

Despite the importance of HM for the infant nutrition, health and development, and the undernutrition and neonatal morbidity burden in the African region, evidence on HM composition during the first 6 months from African women is currently lacking. Therefore, we aim to describe the HM composition of 48 lactating women from birth until 6 months in Bangui (Central African Republic). The analyses of fatty acids (FAs), AAs, lactose, retinol, and HMOs levels through this critical period of life will shed light on HM composition, a first step to address the effect of HM on infant health in the African region.

## Materials and methods

### Study design

**Figure 1:**
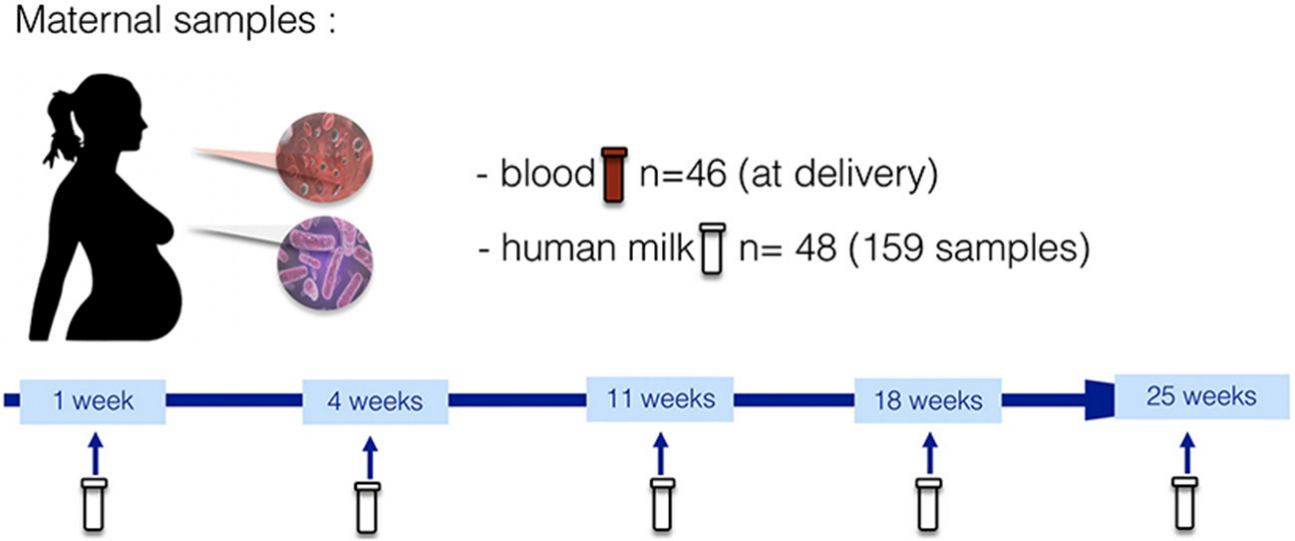
Maternal biological sampling and data collection timeline in the MITICA study.

The HM analyses were performed in the context of the “Mother-to-Infant Transmission of microbiota in Central Africa” (MITICA) study, an observational cohort whose main objective was to describe the mechanisms of acquisition of dysbiotic gut microbiota, associated with pediatric environmental enteric dysfunction (EED) and stunting, in Central Africa. More precisely, the MITICA cohort aimed to investigate the role of mothers' malnutrition in priming conditions for child intestinal dysbiosis. Forty-eight pregnant women were recruited and followed from delivery until 6 months with their 50 vaginally born infants in Bangui, the capital of the Central African Republic. After birth, mother-infant couples were followed through scheduled visits at postpartum weeks 1, 4, 11, 18, and 25. At each visit, a HM sample was collected and analyzed for HMOs, lactose, FAs, and AAs. Additionally, infant feeding practices and maternal dietary intake were assessed using: (i) a 24-h recall questionnaire; (ii) a food-consumption questionnaire (including feeding practices); and (iii) a food security questionnaire at each follow-up visit. Complementary information on socio-economic status and hygienic measures was also gathered. The concrete timeline of HM sampling and data collection is presented in Figure 1.

### Study setting, local engagement, and ethical clearance

Pregnant women were pre-included either at the Henri Izamo maternity (also known as “Gendarmerie” maternity) during antenatal visits or in the neighborhoods surrounding the maternity with the support of community workers. The population of these neighborhoods are mainly state-workers and students of middle-class Central African families. The study was explained to the women (and, possibly, to their family) in Sango (the local language) and their voluntary consent was obtained before enrollment. Over 200 pregnant women were pre-included. The recruitment period (December 2017–June 2019) was set in advance due to logistic and financial constraints.

The MITICA study was approved by the Ethics Committee of the Faculty of Sciences of Bangui (Approval number 9/UB/FACSS/CSVPR/17), the Ministry of Health of the Central African Republic (Approval number 189/MSP/DIRCAB/DGPGHV/DGEHU), and the Institutional Review Board of the Institut Pasteur in France (Approval number 2016-09/IRB). The Municipality of Bangui is divided into administrative units (“arrondissements”), which are composed of different neighborhoods managed by a local authority (“chef de quartier”). The study was presented to all “chefs de quartier” of the neighborhoods involved in the recruitment process, and their written consent was obtained. The Henri Izamo maternity is managed by the Central African Republic Army. Consequently, the additional consent of the Ministry of the Defense was also obtained.

### Study participants

At delivery, pre-included pregnant women attended the Henri Izamo maternity. Women with severe conditions or women requiring a Caesarian-section were transferred to other maternities in Bangui. Otherwise, delivering women were tested for HIV, HBV, and HCV. If one of the tests was positive, women were tested again for diagnostic confirmation and, if necessary, referred to the National Program, after delivery. All women delivering within the laboratory opening hours (8 AM−2 PM) with negative rapid diagnostic tests results for HIV, HBV, and HCV during this period were *de facto* included in the cohort. Forty-eight mother-infant couples (including two couples of twins) were included from December 8th, 2017 to June 29th, 2019.

### Inclusion and non-inclusion criteria

Pregnant women should be ≥ 15 years old, planning to deliver at the Henri Izamo maternity, and should have been pre-included at least at 20 weeks of pregnancy. All pregnant women with a pre-inclusion signed document were asked for further participation agreement at delivery. The HIV, HBV, and HCV Rapid Diagnostic Tests performed upon arrival at the maternity ward for delivery should be negative, and delivery should be vaginal. Finally, the health status of women at inclusion should be fit enough to allow all exams and procedures. Due to the sample treatment requirements and the Institut Pasteur de Bangui (IPB) laboratory working schedule, only women delivering between 8 AM and 2 PM were included in the cohort.

Women without a previous pre-inclusion document were not included in the study cohort. Included women should not have diagnosed or suspected previous pathologies (such as arterial hyper-tension, diabetes, renal failure, mental disorders), nor pregnancy-related complications (placenta previa, pre-eclampsia, previous history of premature labor, infections, early cervical dilatation, premature rupture of the membranes, or vaginal bleeding). Women with signs of ongoing diseases, such as malaria-related symptoms, respiratory stress, diarrhea, cardio-vascular symptoms, etc., were also excluded, as well as obese women (body-mass-index (BMI) > 27 kg/m^2^). Treatments during the previous 2 months with anti-helminths, antibiotics, food or nutritional supplements (except micronutrient supplements) were considered further exclusion criteria.

### Mothers' questionnaires and procedures at delivery

At delivery, the maternity midwife performed a complete physical examination encompassing anthropometric parameters, temperature, blood pressure, and assessment of edema, in addition to a full obstetric exam. This exam included symphysis fundal height (SFH), essential for the estimation of gestational age, uterus dilatation, integrity of the membranes, and a fetal heart-beat monitoring. Anthropometric parameters' assessment included BMI, mid-upper arm circumference (MUAC), but also bicipital, tricipital, pectoral, subscapular, abdominal, quadricipital, and supra-iliac skinfolds were measured. MUAC is an easy-to-perform technique, considered as a good proxy for BMI in pregnant women (23). Skinfold thicknesses are useful non-invasive tools to ascertain the fat content of the body (24–26). Albeit considerable efforts have concluded to concrete BMI and MUAC ranges aiming to define undernourishment in pregnant women (27), albumin concentration in venous blood seems to be a more accurate measure of undernourishment among pregnant women (28–30). Therefore, in the MITICA study, undernourishment status was defined according to albumin levels <35 g/l (28–30).

After birth, the clinical research associate, a trained anthropologist, interviewed the mother to complete a 24-h recall questionnaire, a food-consumption questionnaire (including feeding practices), and supplementary information on the food security status of the household, its socio-economic status, and childcare hygienic measures, in addition to the mother's decisional capacity in the household. Blood samples (8 ml) were drawn at delivery from women to determine albumin, hemoglobin, ferritin, CRP, vitamin A, C, and E levels in addition to a complete blood count.

### Maternal and infant follow-up visits

Follow-up visits were scheduled at 1, 4, 11, 18, and 25 weeks. The entire follow-up was scheduled and agreed with the mothers after delivery. However, mothers were reminded of their next upcoming visit by telephone 3 days before the scheduled day. In case of a missed visit, the visit was rescheduled, when possible. Then, the clinical research associate filled the same questionnaires as at delivery, except for the socio-economic questionnaire (24-h recall, food-consumption questionnaire, food security, and childcare hygienic measures).

### Blood biochemistry analyses

Complete cell blood counts (CBC) and hemoglobin analyses were performed using Horiba's Yumizen 500 and Pentra XLR, respectively. Ferritin levels were analyzed using BioMérieux' multiparametric VIDAS. Plasmatic CRP and albumin levels were assessed using Horiba's Pentra 400. WHO defined in 2020 new cut-off for iron deficiency determined by serum ferritin levels <70 ng/ml for adults in presence of inflammation (CRP > 5 mg/dl) (31). There was no specific recommendation for pregnant women in these recent WHO guidelines, but as in MITICA study, 60% of women had ongoing inflammation, which could lead to underestimation of iron deficiency prevalence assessment, we decided to apply cut-off values of serum ferritin <15 ng/ml in the absence of inflammation and <70 ng/ml in presence of inflammation, according to these guidelines (31).

For vitamin determination, blood was collected in a lithium-heparin tube and was immediately centrifuged for 15 min at 3,000 r/min at 4°C. For vitamin A and vitamin E, 100 μl of serum were placed into a cryotube and stored at −80°C at the IPB before being transferred to the Cochin Hospital in Paris (France) within 2 months. There, vitamin A and vitamin E concentrations were analyzed using a high-performance liquid chromatography (HPLC) Ultimate 3000 (Thermo Scientific) through a HPLC inverse phase and UV detection technique at the Biochemistry service. For vitamin C analyses, 200 μl of plasma were combined with 200 μl of a deproteinization solution of 2 g of meta-phosphoric acid and 15 ml 0.1% EDTA. This mix was vortexed for 1 min, incubated for 10 min at 4°C, centrifuged for 4 min at 10,000 r/min at 4°C. Then it was stored at −80°C until its transfer to the Cochin Hospital, where vitamin C levels were determined using HPLC Ultimate 3000 (Thermo Scientific) through an HPLC inverse phase and UV detection technique at the Biochemistry service. According to WHO definitions (32), vitamin A deficiency was defined by vitamin A levels <1 μmol/l and vitamin E deficiency was defined by vitamin E levels <11.6 μmol/l (33). Hypovitaminosis C was defined by serum vitamin C levels <28 μmol/l and vitamin C deficiency was defined by vitamin C levels <11 μmol/l.

### Human milk sampling and analyses

Human milk samples were manually taken by the mother at each follow-up visit at 1, 4, 11, 18, and 25 weeks (Figure 1). More precisely, between 10 and 12 AM the mother poured 8 ml of her breastmilk manually (from one breast without any preference) into a sterile tube before breastfeeding the infant and, at least, 2 h after the last breastfeeding session. The nipple and areola were not cleaned before pouring the foremilk, as women do not clean systematically the breast before breastfeeding. After sampling, the foremilk aliquots were immediately transferred into a −80°C freezer located at the IPB (same site where the sampling took place), and then sent on dry-ice to the Institut Pasteur (Paris, France) where human milk samples were pasteurized to avoid any possible infectious contamination. After this treatment, HM samples were sent on dry-ice to Danone Nutricia Research laboratory facilities in Utrecht (The Netherlands). Due to the different volumes of HM in the samples, the number of nutritional analyses performed differs depending on the exams: 147 samples had enough volume to perform all analyses (FAs, AAs, retinol and lactose, and HMOs); and up to 158 samples had enough volume to perform FA analyses.

### Determination of fatty acids and amino acids in human milk

Human milk samples were thawed overnight at 4°C and then gently vortexed and aliquoted. Two 250 μL milk samples were analyzed for either AA or FA content and composition by standard methods described elsewhere in detail (34–36). The milk samples used for FA analysis were spiked prior to lipid extraction (37) with C19:0 to enable quantification. Fatty acid composition was analyzed by means of a gas chromatograph (GC) equipped with a flame ionization detector (FID). Processing and derivatization was according to Morrison and Smith (34). Precisely, for the poly-unsaturated fatty acids (PUFA), a certain amount of C19:0 PC was added (as a standard) to 100 μl HM. The HM lipids were converted to fatty acid methyl esters with methanol and 2% sulphuric acid at 100°C for 60 min. We extracted the fatty acid methyl esters (FAMEs) using hexane and, after evaporation, they were dissolved in isooctane. We injected 1 μl of isooctane into the GC. We separated FAMEs on a CP-Sil 88 column and detected them using a FID detector. The identification of FAME was based on retention time. The absolute concentration was calculated after normalization with the C19:0 peak, and the relative concentration was based on the peak area.

The methodology we employed to analyze amino acids was based on Teerlink et al. (36). More concretely, the HM proteins were broken down to amino acids by acid hydrolysis with hydrochloric acid. The concentration of the different amino acids in the hydrolysate was determined by ultra-fast liquid chromatography (UFLC) using a pre-column derivatization with o-phtaldialdehyde and fluorimetry as a detection method. After precipitation of proteins and polypeptides with perchloric acid, the HM sample was centrifuged. Both levels of FAAs (i.e., unbound AA) and total amino-acids (TAAs; i.e., unbound + conjugated AA) were analyzed by liquid chromatography as described in detail elsewhere (35, 36). This method omitted the detection of proline and cysteine, yielding a total of 18 detectable FAAs and the non-proteogenic AA taurine. The measurements of TAA required acidic hydrolysis of proteins, which enabled the detection of 15 TAA and disabled the detection of tryptophan, cysteine, and proline. The acidic hydrolysis process also transformed asparagine into aspartate and glutamine into glutamate, disabling the detection of these TAA individually. In parallel, the FAA analysis does not enable us to detect tryptophan, cysteine, and proline, yielding a total of 18 detectable FAAs, and taurine.

### Quantitation of lactose and human milk oligosaccharides by targeted LC-MS/MS

Quantitative determination of individual HMOs structures by targeted liquid chromatography mass spectrometry (LC-MS)/MS analysis was conducted using a validated method as essentially described by Mank et al. and Siziba et al. (38, 39). Briefly, HM samples had been thawed before at room temperature and were vortexed. We added 15 μl of α-L- arabinopentaose as internal standard solution ITS (0.05 mmol/l) to 135 μl of HM. This was further diluted 1:11 (v/v) in 2-ml eppendorf test tubes by adding 1,350 μl H_2_O (used as LC-MS grade). Then we transferred 450 μl of this diluted mixture into a 500-μl Amicon Ultra centrifugal filter device with a 3-kDa cutoff. Precisely, the ultrafiltration (UF) was performed at 14,000 g for 1 h. We transferred the UF permeate into a 300-μl LC-MS vial with screw top (Thermo Fisher Scientific, Waltham, MA, USA). Then either we performed LC-MS analysis directly or we stored samples at −20°C until further use. All sample preparation procedures were conducted at room temperature. The concrete parameters of the negative ion mode multiple reaction monitoring LC-ESI-MS2 analyses [including a 1,100 series HPLC stack (Agilent, Santa Clara, CA, USA) with a solvent rack, degasser, binary solvent pump, high-performance autosampler, column oven, and DAD detector] are presented in detail elsewhere (22).

Quantification of absolute HMO concentrations could be performed for 16 HMO structures and lactose. The analyzed HMOs comprised: 2'-fucosyllactose (2'-FL), 3-fucosyllactose (3-FL), 3'-sialyllactose (3'-SL), β4'-galactosyllactose (4'-GL), β6'-galactosyllactose (6'-GL), 3,2'-difucosyllactose (DFL), 6'-sialyllactose (6'-SL); lacto-N-tetraose (LNT), lacto-N-neotetraose (LNnT), lacto-N-fucopentaose-I (LNFP I), lacto-N-fucopentaose-II (LNFP II), lacto-N-fucopentaose-III (LNFP III), lacto-N-fucopentaose-V (LNFP V), Lacto-N-difucohexaose I (LNDFH I), and the sum of the co-eluting Lacto-N-difucohexaose II and Lacto-N-neodifucohexaose II (LNDFH II + LNnDFH II).

Determination of human milk groups (types) based on specific HMO-markers was performed as follows: Human milk samples were assigned to HM-type IV if LNFP I, LNFP II, and LNDFH I were below the lower limit of quantification (LLOQ). HM-type III was assigned if LNFP II and LNDFH I were below LLOQ. HM type II was assigned if LNFP I and LNDFH I were below LLOQ. Finally, all residual HM samples were categorized to belong to HM-type I. Simpson's Diversity index of HMOs was calculated as the reciprocal sum of the square of the relative abundance of each of the measured HMOs.

### Human milk retinol determination

Full HM samples were treated at ambient temperature with an ethanolic potassium hydroxide solution for 15–20 h. Precisely, the potassium hydroxide solution consisted in 45 g potassium hydroxide dissolved in 80 g demi water. For the saponification solution, 0.5 g ascorbic acid was dissolved in 25 ml of potassium hydroxide solution. The extraction solution was prepared with acetonitrile/acetic acid 95/5 (v/v). The mix was placed in an autosampler vial tube with 0.2 ml ethanol and 0.1 ml of saponification solution and 0.1 ml sample. The headspace was filled with Nitrogen and the vials were covered. The solutions standed for 15–20 h in the dark room. Then 0.8 ml of the extraction solution were added and extracted for 1 min. The mix was centrifuged for 2 min. at 2,500 rpm. Finally 10 μl of the upper layer were injected into the HPLC. The content of retinol in the extract was determined by HPLC using UV properties for detection by comparing with standard solutions.

### Statistical analyses

Questionnaire's data were gathered on the field using REDCap (40, 41) electronic data tools hosted at Institut Pasteur. The Skillings-Mack test was used to analyze the evolution of the continuous variables in human milk during follow-up (FAs, AAs, HMOs, lactose, and retinol levels). This non-parametric test is a generalization of the Friedman's ANOVA method and Durbin's rank test for data obtained from block designs with random missing observations. Its *P*-value is based on the chi-squared distribution and Monte Carlo method. Mixed-models with a random intercept at the mother's levels were used to evaluate multivariate analyses of the different HM nutrients (FAs, AAs, HMOs, lactose, and retinol levels). Observations with missing values were omitted from the models. Due to the infant death (not related to the study procedures) two women were lost-to-follow-up and they were not included in the final analyses. Data at the baseline did not differ significantly between included women and lost-to-follow-up women. These statistical analyses and box plots on the distribution of the variables during follow-up were performed using Stata MP Software (Stata Corp, College Station, TX, USA). The article has been written following the Strengthening the Reporting of Observational Studies in Epidemiology (STROBE) statement (42).

## Results

### Description of the cohort and maternal characteristics at delivery

Forty-eight women were included in the MITICA cohort between December 2017 and June 2019. At delivery, women were aged 15–39 years, and the median age was 23 years. Over 10% of them were primigravidae. Forty out of 48 women (83.3%) had secondary or higher education, 22 (45.8%) worked at home, and 24 (50.0%) were students. Table 1 summarizes the most significant information of the included women. Sixteen women out of the 46 women with an albumin measure (34.8%) had a BMI <23 kg/m^2^–a gross proxy for undernourishment status in pregnant women (27), and eight were anemic (8/43 with a hemoglobin result at delivery = 18.6%). Micronutrient deficiencies were highly prevalent among these women. Eighteen out of 43 women (37.5%) were iron-deficient, 23/37 (62.2%) had vitamin A deficiency, 19/45 (42.2%) had vitamin C deficiency, and 5/38 (13.2%) had vitamin E deficiency. There were significant differences in the Household Food Insecurity Access Scale (HFIAS) between undernourished and not undernourished mothers at delivery (*P*-value = 0.049). Concrete data of the maternal diet at delivery, and during the 6 first months of the infant's life are presented elsewhere (43), but, briefly, Central-African women ate a significantly less number of meals during the 1st week after birth, compared to the rest of the follow-up (*P*-value < 0.001), and their Women's Dietary Diversity Score (WDDS) was also significantly lower right after birth (*P*-value < 0.001), compared to later periods. The maternal diet was monotonous, and mainly based on tapioca, peanut oil, peanut paste, local doughnuts, onion, some meat or dried fish and dark-green leafy vegetables (spinach, amaranth, and cassava leaves). At delivery, and according to the 24-h recalls, undernourished women consumed significantly more dark-green leafy vegetables than non-undernourished women (*P*-value = 0.01), and significantly less milk and dairy products (*P*-value = 0.04), compared to non-undernourished women.

**Table 1:** Baseline characteristics of the women at delivery.

Variables*	***n* (proportion) or median (95% CI)**
Number of participants	48
Age (years)	22.7 (20.7; 29.6)
Gravidity	2 (1, 3)
Primigravidae	5 (10.4%)
1–3 previous gestations	38 (79.2%)
4+	5 (10.4%)
**Education**	
Primary	8 (16.7%)
Secondary	33 (68.8%)
Higher	7 (14.6%)
**Occupation**	
Homemaker	22 (45.8%)
Work outside home	2 (4.2%)
Student	24 (50.0%)
Undernourishment status at delivery**	16 (34.8%)
Weight (kg)	63.5 (59.5; 67.5)
Height (m)	1.6 (1.6; 1.7)
BMI (kg/m^2^)	25.0 (23.6; 26.3)
BMI <23 (undernourishment in pregnant women; kg/m^2^)	8 (16.7%)
Fever (T > 38 C)	3 (6.3%)
Albumin (g/l)	36.0 (33.0; 38.0)
Hemoglobin (g/dl)	12.4 (11.2; 13.8)
Anemia (Hemoglobin <11 g/dl)	8 (17.4%)
Ferritin (ng/ml)	48.0 (27.0; 105.0)
Iron deficiency***	20 (41.7%)
Vitamin C plasmatic levels (μmol/l)	15.3 (6.2; 27.8)
Vitamin C hypovitaminosis (vitamin C levels <28 μmol/l)	35 (77.8%)
Vitamin C deficiency (vitamin C levels <11 μmol/l)	19 (42.2%)
Vitamin A plasmatic levels (μmol/l)	0.9 (0.8; 1.2)
Vitamin A deficiency (vitamin A levels <1 μmol/l)	23 (62.2%)
Vitamin E plasmatic levels (μmol/l)	26.2 (20.6; 30.7)
Vitamin E deficiency (vitamin E levels <11.6 μmol/l)	5 (13.2%)
CRP (mg/l)	7.0 (1.8; 26.2)
Inflammation (CRP > 5 mg/l)	28 (60.9%)

*For continuous variables, median and inter-quartile range are provided. For binary and variables with categories, n and percentages are provided.

**Undernourished status was defined according to albumin levels <35 g/l. The albumin values are missing for five women.

***Iron deficiency is defined by serum ferritin levels <15 ng/ml in the absence of inflammation. In the presence of inflammation, iron deficiency is defined by serum ferritin levels <70 ng/ml.

### Human milk-type distribution

**Figure 2:**
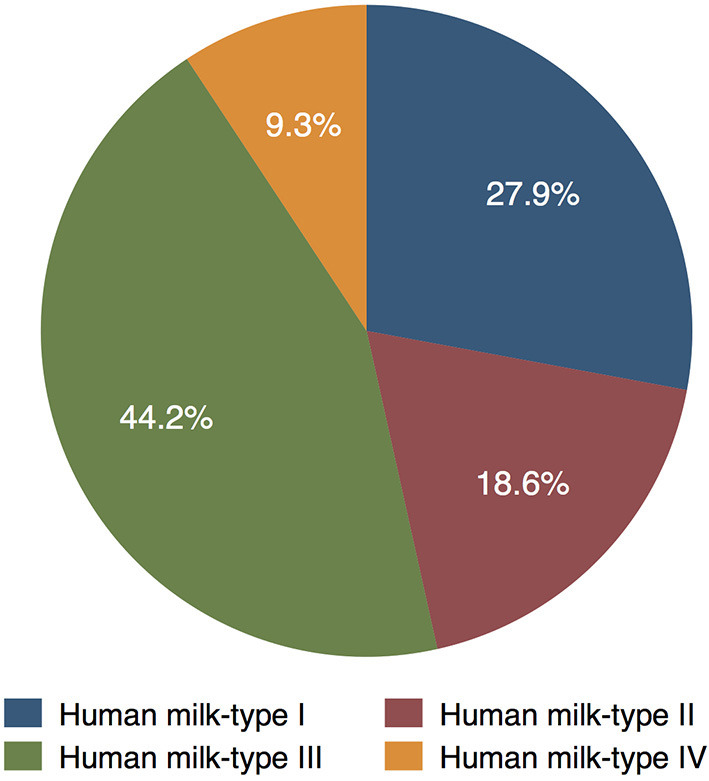
Distribution of HM-types among women in the MITICA cohort (as percentage of the 43 women with HM samples).

In the MITICA cohort, 12 women were Se+ and Le+ [corresponding to HM-type I (27.9%)], eight women were Se- and Le+ [HM-type II (18.6%)], 19 women were Se+ and Le- [HM-type III (44.2%)], and four women were Se- and Le- [HM-type IV (9.3%; Figure 2)]. Altogether, 31 (72%) women were secretors and 12 (28%) were non-secretors. HMOs were determined only in 154 of the 159 samples analyzed, we assumed that the five samples not analyzed (for HMOs) belonged to the same HM-type as the other samples of the same woman. Graphical information on HM-type distribution is shown in Figure 2. Despite missing HM samples at certain visits, there were no significant differences in the number of samples belonging to the different HM-types during follow-up.

Interestingly, the proportion of male infants among HM-type I more than doubled the proportion of female infants [8/20 (40.0%) boys vs. 4/23 (17.4%) girls], and the proportion of female infants almost doubled the proportion of male infants among HM-type III infants [13/23 (56.5%) females vs. 6/20 (30%) males]. However, the distribution of males and females was similar among HM-types II and IV [4/23 (17.4%) girls vs. 4/20 (20%) boys, and 2/23 (8.3%) girls vs. 2/20 (10%) boys, respectively]. This gender differences between HM-types correlated with the overall distribution of the different milk-types did not reached statistically significance according to the Fisher test when considering the number of infant but, when considering the total HM samples gathered during follow-up (n=159), there were overall significant differences in HM-type of boys and girls (*P*-value = 0.007 in the Fisher test). Precisely, there were 15/84 (17.9%) samples of girls vs. 31/75 (41.3%) samples of boys among HM-type I, 16/84 (19.1%) samples of girls vs. 13/75 (17.3%) samples of boys among HM-type II, 45/84 (53.6%) samples of girls vs. 24/75 (32.0%) samples of boys among HM-type III, and 8/84 (9.5%) samples of girls vs. 7/75 (9.3%) samples of boys among HM-type IV infants.

### Human milk oligosaccharides

The specific distribution of HMOs and HMO diversity score during follow-up is shown in Table 2. Total detected HMOs levels decreased significantly during the first 6 months. At week 1, the median levels of total detected HMOs were 7.3 g/l [inter-quartile-range (IQR) = 6.2; 8.8]. Then they decreased to 5.4 g/l (IQR = 4.7; 6.1) at week 4, 4.3 g/l (IQR = 3.6; 5.3) at week 11 and 4.5 g/l (IQR = 3.8; 5.4) at week 18 and down to 3.5 g/l (IQR = 3.1; 4.1) at week 25. In parallel, HMO diversity also decreased significantly depending on the lactation period: from 3.7 (IQR = 2.5; 4.4) at week 1, to 3.6 (IQR = 3.0; 4.7) at week 4, 3.1 (IQR = 2.3; 4.3) at week 11, 3.0 (IQR = 2.4; 4.2) at week 18, and 2.9 (IQR = 2.2; 3.8) at week 25. Absolute values of 2'-FL, 3'-SL, 6'-SL, 6'-GL, LNT, LNnT, LNFP I, and LNDFH I also decreased significantly from birth until 6 months. In contrast to the overall trend of decreasing HMOs levels during follow-up, the absolute values of concentrations of 4'-GL, DFL, and LNDFH II + LNnDFH II in HM remained constant during the first 6 months of life, and 3-FL levels increased significantly during this period. Relative values of 2'-FL, 3-FL, 3'-SL, 4'-GL, DFL, LNFP II, LNFP III, LNFP V, LNDFH I, and LNDFH II + LNnDFH II increased significantly until 6 months, although 3'-SL decreased a slightly at the beginning. On the contrary, relative values of 6'-SL, 6'-GL, LNT, and LNFP I diminished significantly until 6 months.

**Table 2:** Human milk-types and HMO content during follow-up.

	**Total**	**1 week**	**4 weeks**	**11 weeks**	**18 weeks**	**25 weeks**	*P*-value*
**Number of samples**	154	35	30	34	25	30	
**Human milk-types**							
Type I	43 (27.92%)	8 (22.86%)	9 (30.00%)	8 (23.53%)	8 (32.00%)	10 (33.33%)	
Type II	29 (18.83%)	6 (17.14%)	7 (23.33%)	7 (20.59%)	3 (12.00%)	6 (20.00%)	
Type III	68 (44.16%)	17 (48.57%)	11 (36.67%)	16 (47.06%)	13 (52.00%)	11 (36.67%)	
Type IV	14 (9.09%)	4 (11.43%)	3 (10.00%)	3 (8.82%)	1 (4.00%)	3 (10.00%)	
**Human milk oligosaccharides (absolute value)**							
Total detected HMOs (g/l)	4.76 (3.73; 6.23)	7.33 (6.17; 8.78)	5.41 (4.70; 6.11)	4.25 (3.60; 5.25)	4.45 (3.75; 5.36)	3.46 (3.11; 4.09)	**<0.001**
2'-fucosyllactose (2'-FL; g/l)	2.00 (0.10; 3.10)	2.60 (0.10; 4.50)	1.80 (0.10; 2.80)	1.75 (0.10; 3.10)	2.20 (1.20; 3.30)	1.45 (0.10; 2.20)	**<0.001**
3-fucosyllactose (3-FL; g/l)	0.22 (0.04; 0.72)	0.08 (0.04; 0.28)	0.23 (0.05; 0.80)	0.23 (0.04; 0.88)	0.24 (0.04; 0.72)	0.43 (0.08; 0.85)	**<0.001**
3'-sialyllactose (3'-SL; g/l)	0.15 (0.11; 0.20)	0.22 (0.17; 0.28)	0.15 (0.12; 0.18)	0.11 (0.09; 0.16)	0.15 (0.12; 0.17)	0.11 (0.08; 0.14)	**<0.001**
6'-sialyllactose (6'-SL; g/l)	0.14 (0.04; 0.32)	0.51 (0.51; 0.51)	0.22 (0.16; 0.30)	0.08 (0.05; 0.14)	0.05 (0.03; 0.11)	0.02 (0.02; 0.04)	**<0.001**
4'-galactosyllactose (4'-GL; g/l)	0.001 (0.001; 0.001)	0.001 (0.001; 0.001)	0.001 (0.001; 0.001)	0.001 (0.001; 0.001)	0.001 (0.001; 0.001)	0.001 (0.001; 0.001)	0.29
6'-galactosyllactose (6'-GL; g/l)	0.02 (0.01; 0.05)	0.05 (0.05; 0.05)	0.03 (0.02; 0.05)	0.02 (0.01; 0.03)	0.01 (0.01; 0.02)	0.03 (0.02; 0.14)	**<0.001**
Difucosyllactose (DFL; g/l)	0.02 (0.02; 0.11)	0.02 (0.02; 0.11)	0.02 (0.02; 0.11)	0.02 (0.02; 0.09)	0.06 (0.02; 0.13)	0.02 (0.02; 0.14)	0.47
Lacto-N-tetraose (LNT; g/l)	0.79 (0.45; 1.50)	1.70 (0.96; 2.80)	1.10 (0.74; 1.90)	0.65 (0.45; 0.92)	0.60 (0.40; 0.82)	0.49 (0.22; 0.71)	**<0.001**
Lacto-N-neotetraose (LNnT; g/l)	0.12 (0.06; 0.20)	0.18 (0.07; 0.25)	0.13 (0.08; 0.22)	0.12 (0.04; 0.17)	0.13 (0.06; 0.18)	0.07 (0.03; 0.15)	**0.002**
Lacto-N-fucopentaose I (LNFP I; g/l)	0.25 (0.04; 0.82)	0.91 (0.04; 1.60)	0.41 (0.04; 0.85)	0.23 (0.04; 0.55)	0.29 (0.07; 0.57)	0.08 (0.04; 0.18)	**<0.001**
Lacto-N-fucopentaose II (LNFP II; g/l)	0.04 (0.04; 0.31)	0.04 (0.04; 0.35)	0.08 (0.04; 0.49)	0.01 (0.01; 0.05)	0.04 (0.04; 0.14)	0.05 (0.04; 0.17)	**<0.001**
Lacto-N-fucopentaose III (LNFP III; g/l)	0.12 (0.02; 0.22)	0.12 (0.02; 0.26)	0.14 (0.02; 0.24)	0.13 (0.02; 0.22)	0.14 (0.02; 0.20)	0.12 (0.02; 0.19)	**0.003**
Lacto-N-fucopentaose V (LNFP V; g/l)	0.01 (0.01; 0.05)	0.02 (0.01; 0.05)	0.03 (0.01; 0.07)	0.04 (0.04; 0.31)	0.041(0.01; 0.03)	0.01 (0.01; 0.02)	**<0.001**
Lacto-N-difucohexaose I (LNDFH I; g/l)	0.02 (0.02; 0.12)	0.02 (0.02; 0.02)	0.02 (0.02; 0.18)	0.02 (0.02; 0.02)	0.02 (0.02; 0.22)	0.02 (0.02; 0.08)	**<0.001**
Lacto-N-difucohexaose II + lacto-N-neodifucohexaose II (LNDFH II + LNnDFH II; g/l)	0.01 (0.01; 0.03)	0.01 (0.01; 0.01)	0.01 (0.01; 0.05)	0.01 (0.01; 0.03)	0.01 (0.01; 0.02)	0.01 (0.01; 0.03)	0.18
**Human milk oligosaccharides (percentage of total detected HMOs)**							
2'-fucosyllactose (2'-FL; %)	40.94 (4.11; 57.31)	37.21 (2.37; 48.07)	31.22 (2.90; 48.37)	43.34 (4.34; 60.52)	53.60 (26.94; 59.76)	43.11 (4.83; 57.31)	**<0.001**
3-fucosyllactose (3-FL; %)	3.51 (0.92; 15.39)	1.87 (0.52; 3.54)	3.98 (0.86; 14.16)	6.45 (0.93; 21.16)	4.96 (0.92; 15.49)	14.95 (2.36; 23.80)	**<0.001**
3'-sialyllactose (3'-SL; %)	2.98 (2.14; 4.14)	3.10 (2.09; 4.38)	2.56 (2.02; 3.54)	2.53 (2.02; 3.89)	3.00 (2.29; 3.97)	3.26 (2.33; 4.43)	**<0.001**
6'-sialyllactose (6'-SL; %)	2.70 (1.01; 5.08)	6.54 (5.28; 8.18)	4.29 (2.96; 5.26)	1.99 (1.49; 3.04)	0.93 (0.71; 2.02)	0.63 (0.46; 1.26)	**<0.001**
4'-galactosyllactose (4'-GL; %)	0.02 (0.02; 0.03)	0.02 (0.02; 0.02)	0.02 (0.02; 0.03)	0.03 (0.02; 0.03)	0.02 (0.02; 0.03)	0.03 (0.03; 0.04)	**<0.001**
6'-galactosyllactose (6'-GL; %)	0.46 (0.29; 0.69)	0.66 (0.54; 0.78)	0.55 (0.39; 0.81)	0.45 (0.34; 0.64)	0.27 (0.22; 0.36)	0.30 (0.22; 0.47)	**<0.001**
Difucosyllactose (DFL; %)	0.60 (0.40; 1.98)	0.41 (0.26; 1.37)	0.53 (0.35; 1.49)	0.59 (0.47; 1.98)	1.17 (0.45; 2.92)	0.86 (0.53; 3.88)	**<0.001**
Lacto-N-tetraose (LNT; %)	16.38 (9.10; 30.13)	21.37 (10.17; 40.18)	20.49 (12.97; 35.03)	16.13 (8.48; 22.36)	12.84 (10.34; 19.22)	13.62 (6.57; 21.45)	**<0.001**
Lacto-N-neotetraose (LNnT; %)	2.43 (1.17; 3.87)	2.51 (1.17; 3.52)	2.43 (1.44; 3.33)	2.62 (1.08; 4.30)	2.18 (1.39; 4.73)	1.98 (0.67; 3.95)	0.051
Lacto-N-fucopentaose I (LNFP I; %)	5.90 (1.23; 13.36)	11.95 (0.95; 19.91)	7.60 (1.07; 14.51)	5.13 (1.26; 10.65)	7.88 (1.74; 13.36)	2.16 (1.41; 5.82)	**0.004**
Lacto-N-fucopentaose II (LNFP II; %)	1.18 (0.75; 6.98)	0.81 (0.52; 4.59)	1.31 (0.74; 13.95)	1.26 (0.80; 10.58)	1.09 (0.79; 3.25)	1.71 (1.10; 6.07)	**<0.001**
Lacto-N-fucopentaose III (LNFP III; %)	2.78 (0.63; 4.91)	1.72 (0.34; 4.33)	2.86 (0.58; 4.31)	3.00 (0.63; 5.45)	2.80 (0.60; 5.27)	3.28 (0.75; 5.80)	**<0.001**
Lacto-N-fucopentaose V (LNFP V; %)	0.33 (0.21; 1.13)	0.24 (0.14; 0.73)	0.54 (0.20; 1.52)	0.35 (0.22; 1.45)	0.28 (0.21; 0.63)	0.37 (0.27; 0.88)	**<0.001**
Lacto-N-difucohexaose I (LNDFH I; %)	0.62 (0.44; 2.89)	0.38 (0.31; 0.57)	0.54 (0.43; 3.81)	0.66 (0.48; 1.66)	0.64 (0.48; 4.79)	0.82 (0.61; 2.23)	**0.001**
Lacto-N-difucohexaose II (LNDFH II; %) + lacto-N-neodifucohexaose II (LNnDFH II; %)	0.27 (0.20; 0.92)	0.17 (0.13; 0.26)	0.28 (0.20; 1.33)	0.29 (0.21; 1.16)	0.27 (0.21; 0.44)	0.36 (0.28; 0.94)	**<0.001**
HMO diversity score	3.34 (2.43; 4.32)	3.66 (2.49; 4.39)	3.56 (2.99; 4.68)	3.09 (2.28; 4.27)	3.00 (2.40; 4.18)	2.87 (2.23; 3.77)	**<0.001**

*P-value on the evolution of the different items during follow-up. Median and inter-quartile range are provided. P-values in bold are statistically significant.

**Figure 3:**
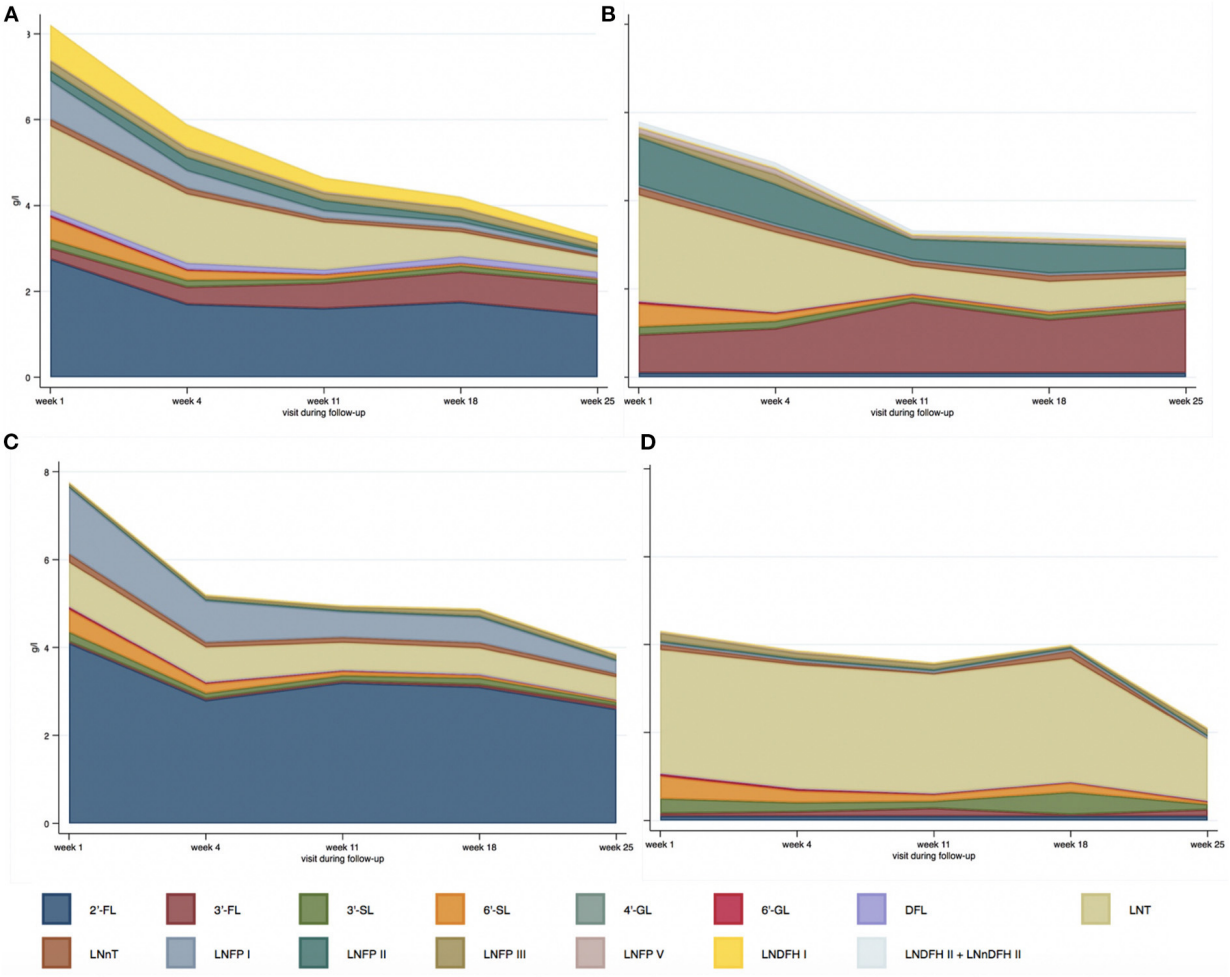
Median absolute values of HMOs (g/l) depending on human milk-type across lactation period [**(A)** HM-type I; **(B)** HM-type II; **(C)** HM-type III; **(D)** HM-type IV].

Significant statistical differences in HMO concentration were found among the different HM-types, except for 4'-GL, 6'-SL, LNnT, and LNFP-III. Figure 3 and Supplementary Figure 1 present the distribution of HMOs among HM-types and their evolution during lactation.

Precisely, adjusted for lactation period, and compared to HM-type III (the most prevalent), 2'-FL levels were lower in HM-type I [adjusted ß-coefficient (aß-coef) = −1.3, *P*-value < 0.001]; 3-FL was higher in HM-type I and HM-type II (aß-coef = 0.4, *P*-value < 0.001; and aß-coef = 1.1, *P*-value < 0.001, respectively); 3'-SL was higher in HM-type IV (aß-coef = 0.1, *P*-value < 0.001); 6'-GL was lower in HM-type I (aß-coef = −0.01, *P*-value = 0.046); DFL was higher in HM-type I (aß-coef = 0.1, *P*-value < 0.001); LNT was higher in HM-type II and HM-type IV (aß-coef = 0.4, *P*-value = 0.02; and aß-coef = 1.6, *P*-value < 0.001, respectively); LNFP V was higher in HM-type I and HM-type II (aß-coef = 0.02, *P*-value = 0.001; and aß-coef = 0.1, *P*-value < 0.001, respectively); and LNDFH II + LNnDFH II were higher in HM-type I and HM-type II (aß-coef = 0.02, *P*-value = 0.04; and aß-coef = 0.1, *P*-value < 0.001, respectively). The significant differences in LNFP I, LNFP II, and LNDFH I levels have not been addressed as their concentration defines the different HM-types. The analyses of HMO relative values are presented in Appendix A.

### Lactose

Lactose concentration varied significantly across the different lactation periods (*P* < 0.001). One week after delivery the median value of lactose concentration in HM was 62.0 g/l (IQR = 60.0; 67.5), similar to the median value 4 weeks after delivery (61.0 g/l, IQR = 56.5; 65.5). Then lactose concentration increased up to a median concentration of 65.0 g/l (IQR = 62.0; 70.5) at 11 weeks, 73.0 g/l (IQR = 70.0; 75.0) at 18 weeks, and 73.0 g/l (IQR = 70.0; 75.0) at 25 weeks months. Indeed, compared to weeks 1 and 4, lactose levels were significantly higher at week 11 [ß-coefficient (ß-coef) = 3.5, *P*-value = 0.01], week 18 (ß-coef = 10.5, *P*-value < 0.001), and week 25 (ß-coef = 10.2, *P*-value < 0.001) after delivery.

Overall, there were no significant differences in lactose concentration depending on HM-types. However, at week 4 after delivery, lactose concentration was significantly higher in HM-type II (aß-coef = 7.8, *P*-value = 0.01), compared to HM-type III.

### Fatty acids

Overall, the concentration of fatty acids in HM was quite stable during the first 6 months of life. The median concentration of fatty acids during the first 6 months of life was 28.5 g/l (IQR = 20.7; 39.9; Table 3). The median concentration of saturated fatty acids during the same period was 14.4 g/l (IQR = 10.3; 20.5) and their median relative contribution to total fatty acids was 49.4 % (IQR = 45.8; 53.1). Unsaturated fatty acids levels were lower: mono-unsaturated fatty acid (MUFA) concentration was 10.2 g/l (IQR = 7.7; 14.7), whereas poly-unsaturated fatty acid (PUFA) level was 3.8 g/l (IQR = 2.6; 5.5). Among PUFAs, omega-6 poly-unsaturated fatty acid (Ω-6 PUFA) concentration was 3.5 g/l (IQR = 2.4; 5.0), while omega-3 poly-unsaturated fatty acids (Ω-3 PUFAs) level was 0.3 g/l (IQR = 0.2; 0.4). Only relative values of arachidonic acid (ARA; 20:4), and absolute and relative values of docosahexaenoic acid (DHA; 22:6) decreased significantly depending on the lactation period during the entire follow-up (*P*-value < 0.001, *P*-value = 0.003, and *P*-value = 0.009, respectively, Table 3). Also, in multilevel models HM concentration of ARA (absolute and relative values), and DHA (absolute and relative values) were significantly lower later on, compared to delivery. Distribution of the different fatty acids in absolute value as box-plots can be found in Supplementary Data 2.

**Table 3:** Fatty acids in human milk during follow-up.

	**Total**	**1 week**	**4 weeks**	**11 weeks**	**18 weeks**	**25 weeks**	*P*-value*
**Number of samples**	158	35	31	37	25	30	
**Fatty acids (absolute values)**							
Total fatty acids (g/l)	28.48 (20.71; 39.93)	32.75 (20.94; 41.87)	30.14 (23.04; 47.22)	26.69 (20.71; 34.98)	26.12 (19.58; 33.95)	31.14 (20.46; 37.83)	0.51
Saturated fatty acids (SFsA, g/l)	14.44 (10.25; 20.49)	16.46 (11.08; 20.26)	14.33 (11.19; 24.38)	13.42 (10.07; 17.11)	13.91 (9.25; 20.46)	15.08 (9.70; 19.73)	0.22
Mono-unsaturated fatty acids (MUFAs, g/l)	10.23 (7.68; 14.71)	11.85 (7.70; 15.80)	10.78 (8.16; 17.25)	9.35 (6.91; 14.05)	9.52 (7.86; 10.74)	10.36 (8.27; 13.99)	0.27
Poly-unsaturated fatty acids (PUFAs, g/l)	3.78 (2.58; 5.46)	4.09 (2.66; 5.54)	3.97 (3.05; 6.48)	3.58 (2.51; 4.99)	3.28 (2.94; 4.79)	3.81 (2.84; 4.92)	0.84
Omega-6 poly-unsaturated fatty acids (Ω 6-PUFAs, g/l)	3.48 (2.45; 4.97)	3.68 (2.42; 5.07)	3.62 (2.90; 5.94)	3.41 (2.19; 4.67)	3.05 (2.73; 4.49)	3.54 (2.63; 4.61)	0.83
Linoleic acid (LA, g/l)	3.17 (2.22; 4.45)	3.19 (2.08; 4.69)	3.24 (2.55; 5.51)	3.14 (1.98; 4.25)	2.79 (2.48; 4.11)	3.25(2.44; 4.31)	0.76
Arachidonic acid (ARA, g/l)	0.09 (0.07; 0.13)	0.12 (0.09; 0.19)	0.09 (0.07; 0.15)	0.08 (0.06; 0.12)	0.08 (0.06; 0.12)	0.09 (0.07; 0.12)	0.29
Omega-3 poly-unsaturated fatty acids (Ω 3-PUFAs, g/l)	0.27 (0.17; 0.42)	0.28 (0.19; 0.44)	0.28 (0.16; 0.44)	0.26 (0.13; 0.34)	0.20 (0.15; 0.36)	0.25 (0.20; 0.36)	0.58
Docosahexaenoic acid (DHA, g/l)	0.10 (0.06; 0.15)	0.12 (0.09; 0.17)	0.12 (0.06; 0.16)	0.06 (0.04; 0.11)	0.08 (0.04; 0.13)	0.09 (0.07; 0.12)	**0.003**
α-Linolenic acid (ALA, g/l)	0.10 (0.06; 0.16)	0.10 (0.06; 0.15)	0.11 (0.06; 0.20)	0.10 (0.06; 0.16)	0.09 (0.06; 0.16)	0.09 (0.07; 0.15)	0.98
Eicosapentaenoic acid (EPA, g/l)	0.02 (0.01; 0.03)	0.01 (0.002; 0.03)	0.02 (0.01; 0.04)	0.01 (0.01; 0.03)	0.02 (0.01; 0.05)	0.02 (0.01; 0.03)	0.63
**Fatty acids (percentage of total fatty acids)**							
Saturated fatty acids (SFAs, %)	49.43 (45.77; 53.09)	51.30 (46.77; 54.78)	48.99 (47.47; 51.63)	48.53 (45.44; 52.60)	48.88 (45.39; 55.33)	50.45 (44.63; 52.78)	0.43
Mono-unsaturated fatty acids (MUFAs, %)	36.94 (34.16; 39.51)	36.44 (32.58; 39.51)	37.38 (34.43; 38.74)	37.12 (34.68; 39.22)	37.10 (33.24; 40.55)	38.18 (34.78; 40.18)	0.22
Poly-unsaturated fatty acids (PUFAs, %)	13.15 (11.63; 14.70)	12.32 (11.45; 14.35)	13.35 (12.10; 14.51)	13.07 (12.03; 15.29)	13.56 (10.66; 14.94)	13.08 (11.38; 14.11)	0.28
Omega-6-poly-unsuturated fatty acids (Ω 6-PUFAs, %)	11.98 (10.72; 13.54)	11.14 (10.27; 13.01)	12.41 (11.31; 13.23)	12.35 (10.83; 13.82)	12.79 (9.83; 14.28)	12.23 (10.53; 13.20)	0.22
Linoleic acid (LA, %)	10.80 (9.47; 12.46)	9.68 (8.52; 11.90)	11.47 (10.15; 12.14)	11.21 (9.76; 12.91)	11.72 (9.16; 13.12)	11.11 (9.64; 12.34)	0.07
Arachidonic acid (ARA, %)	0.33 (0.28; 0.39)	0.42 (0.37; 0.51)	0.30 (0.25; 0.35)	0.32 (0.28; 0.35)	0.30 (0.28; 0.36)	0.31 (0.27; 0.36)	**<0.001**
Omega-3-poly-unsuturated fatty acids (Ω 3-PUFAs, %)	0.88 (0.66; 1.51)	0.88 (0.71; 1.23)	0.92 (0.63; 1.19)	0.87 (0.66; 1.19)	0.83 (0.64; 1.09)	0.87 (0.71; 1.01)	0.96
Docosahexaenoic acid (DHA, %)	0.32 (0.23; 0.41)	0.41 (0.28; 0.51)	0.31 (0.24; 0.41)	0.28 (0.20; 0.35)	0.29 (0.17; 0.42)	0.33 (0.22; 0.39)	**0.009**
α-Linolenic acid (ALA, %)	0.32 (0.26; 0.43)	0.29 (0.24; 0.36)	0.36 (0.25; 0.43)	0.32 (0.29; 0.50)	0.34 (0.34; 0.43)	0.33 (0.25; 0.40)	0.45
Eicosapentaenoic acid (EPA, %)	0.05 (0.02; 0.10)	0.03 (0.01; 0.07)	0.05 (0.02; 0.10)	0.05 (0.03; 0.08)	0.07 (0.02; 0.13)	0.07 (0.04; 0.09)	0.10

*P-value on the evolution of the different items during follow-up. Median and inter-quartile range are provided. P-values in bold are statistically significant.

There were significant differences in the levels of Ω-3 PUFAs (in relative values), eicosapentaenoic acid (EPA, in relative levels), and DHA (in relative values) between the different HM-types using Kruskal-Wallis test (*P*-value = 0.046; *P*-value = 0.04; and *P*-value = 0.03, respectively). Precisely, Ω-3 PUFAs in HM-type I and HM-type II were higher [median = 1.0% (IQR = 0.7; 1.2%); and median = 1.0% (IQR = 0.9; 1.3%), respectively] compared to HM-type III and HM-type IV [median = 0.8% (IQR = 0.6; 1.1%); and median = 0.8 (IQR = 0.7; 1.1%), respectively]. While HM-type II had higher relative levels of EPA [median = 0.1% (IQR = 0.04; 0.12%)], HM-type I, III, and IV had similar relative levels of EPA [median = 0.05% (IQR = 0.03; 0.10%); median = 0.04% (IQR = 0.01; 0.09%); and median = 0.04% (IQR = 0.02; 0.08%), respectively]. In parallel, HM-type II had higher relative levels of DHA [median = 0.4% (IQR = 0.3; 0.5%)], compared to HM-type I, III, and IV, which had similar DHA relative levels [median = 0.3% (IQR = 0.2; 0.4%); median = 0.3% (IQR = 0.2; 0.4%); and median = 0.3 % (IQR = 0.3; 0.4%), respectively].

### Amino acids

#### Total amino acids

The concentration of total amino acids in HM decreased significantly during the first 6 months after delivery (Table 4). Total amino acids values dropped from 12.8 mg/ml at 1 week after delivery (IQR = 11.1; 13.2) to 8.9 mg/ml at 4 weeks (IQR = 8.3; 10.0), 7.9 mg/ml at 11 weeks (IQR = 7.1; 8.6), 7.5 mg/ml at 18 weeks (IQR = 6.5; 8.2), and 7.4 mg/ml at 25 weeks (IQR = 6.8; 8.1). More precisely, total serine, histidine, glycine, threonine, arginine, alanine, tyrosine, valine, methionine, phenylalanine, isoleucine, leucine, lysine, aspartic acid and asparagine, glutamic acid and glutamine (all amino acids analyzed but taurine) decreased significantly in HM during the first 6 months after delivery. The box-plots of total amino acid distribution as absolute value are presented in Supplementary Data 3. The relative levels of total amino acids also differed significantly during follow-up depending on the lactation period (Supplementary Data 4).

**Table 4:** Amino acids in human milk during follow-up.

	**Total**	**1 week**	**4 weeks**	**11 weeks**	**18 weeks**	**25 weeks**	*P*-value*
**Number of samples**	154	36	30	34	25	30	
**Total amino acids**							
**Essential amino acids**							
Histidine (μg/ml)	241.89 (203.26; 311.72)	357.49 (316.84; 394.26)	256.17 (232.20; 283.63)	224.05 (198.14; 247.64)	208.22 (179.52; 229.87)	209.00 (187.74; 225.14)	**<0.001**
Isoleucine (μg/ml)	487.07 (405.61; 599.89)	675.05 (612.74; 775.14)	524.79 (487.01; 580.60)	418.20 (373.34; 469.36)	425.29 (383.44; 488.65)	421.61 (381.34; 478.15)	**<0.001**
Leucine (μg/ml)	988.31 (835.22; 1,216.56)	1,420.29 (1,215.51; 1,534.54)	1,054.36 (957.55; 1,136.81)	910.65 (813.45; 989.23)	868.35 (750.22; 957.81)	854.38 (773.44; 953.81)	**<0.001**
Lysine (μg/ml)	662.83 (564.59; 841.62)	983.13 (864.57; 1,051.25)	713.55 (642.14; 775.76)	618.24 (552.31; 679.20)	568.83 (495.95; 635.49)	591.34 (519.27; 627.59)	**<0.001**
Methionine (μg/ml)	134.74 (108.62; 176.81)	209.04 (177.41; 227.10)	145.78 (131.38; 165.55)	114.29 (107.28; 132.80)	111.76 (91.17; 130.86)	112.80 (102.36; 133.24)	**<0.001**
Phenylalanine (μg/ml)	384.07 (323.61; 502.18)	593.03 (517.87; 630.53)	407.52 (380.68; 463.52)	365.30 (316.67; 395.30)	328.23 (284.21; 371.02)	330.05 (298.50; 353.34)	**<0.001**
Threonine (μg/ml)	453.73 (388.81; 591.79)	714.12 (607.39; 758.44)	485.35 (451.41; 534.61)	421.92 (375.82; 464.33)	390.95 (340.09; 438.18)	399.60 (352.60; 420.26)	**<0.001**
Valine (μg/ml)	578.25 (490.04; 746.60)	861.99 (748.71; 921.74)	624.47 (559.16; 685.09)	527.64 (463.56; 579.42)	499.94 (436.15; 581.12)	515.81 (464.03; 578.25)	**<0.001**
**Non-essential amino acids**							
Alanine (μg/ml)	398.86 (341.84; 518.50)	602.96 (535.70; 660.87)	401.31 (375.11; 473.83)	375.51 (329.28; 409.64)	338.32 (297.96; 387.72)	346.03 (318.67; 382.11)	**<0.001**
Arginine (μg/ml)	295.79 (253.29; 429.58)	506.23 (417.21; 607.61)	317.12 (276.28; 417.12)	289.87 (256.42; 313.39)	247.28 (202.77; 274.63)	268.79 (246.67; 294.57)	**<0.001**
Aspartic acid + asparagine (μg/ml)	946.21 (800.86; 1,226.52)	1,446.00 (1,269.91; 1,532.51)	10,002.64 (931.77; 1,124.30)	904.15 (791.28; 986.80)	810.11 (698.71; 900.95)	814.04 (728.32; 878.19)	**<0.001**
Glutamic acid + glutamine (μg/ml)	1,873.70 (1,634.91; 2,179.88)	2,387.63 (2,202.10; 2,613.18)	1,908.94 (1,769.39; 2,069.16)	1,677.43 (1,615.34; 1,990.23)	1,748.05 (1,554.06; 1,886.35)	1,655.65 (1,526.62; 1,873.11)	**<0.001**
Glycine (μg/ml)	242.78 (209.22; 317.25)	372.95 (323.18; 406.20)	258.09 (223.78; 286.80)	231.59 (197.43; 249.91)	210.95 (179.15; 238.01)	213.35 (190.98; 231.37)	**<0.001**
Serine (μg/ml)	490.67 (425.72; 638.32)	763.79 (668.16; 814.24)	520.35 (480.58; 587.30)	466.49 (408.48; 512.31)	429.77 (372.23; 469.80)	425.72 (390.62; 457.04)	**<0.001**
Taurine (μg/ml)	24.15 (18.40; 31.41)	28.53 (20.65; 37.04)	21.71 (15.02; 33.29)	23.78 (18.02; 28.03)	25.28 (17.83; 29.78)	21.77 (18.52; 31.29)	0.12
Tyrosine (μg/ml)	305.67 (259.83; 398.44)	478.52 (408.04; 524.00)	329.22 (300.23; 380.14)	287.55 (258.74; 314.55)	264.81 (225.85; 296.70)	259.46 (235.00; 282.29)	**<0.001**
Sum of total amino acids (μg/ml)	8,550.70 (7,209.69; 10,923.80)	12,778.92 (11,060.17; 13,212.29)	8,852.44 (8,327.99; 10,033.91)	7,929.39 (7,066.33; 8,644.79)	7,511.92 (6,531.12; 8,247.77)	7,443.45 (6,762.84; 8,090.75)	**<0.001**
**Free amino acids**							
**Essential amino acids**							
Histidine (μg/ml)	4.19 (3.32; 5.40)	5.63 (4.56; 7.56)	3.88 (2.99; 4.83)	4.48 (3.75; 5.07)	3.61 (3.14; 4.11)	3.58 (2.98; 4.20)	**<0.001**
Isoleucine (μg/ml)	2.24 (1.69; 2.74)	2.09 (1.40; 2.68)	1.80 (1.52; 2.45)	2.64 (2.35; 3.49)	2.21 (1.66; 2.53)	2.20 (1.72; 2.79)	**<0.001**
Leucine (μg/ml)	3.99 (3.15; 5.08)	4.30 (3.32; 6.20)	3.55 (3.17; 5.21)	4.28 (3.53; 5.30)	4.07 (2.75; 4.51)	3.37 (2.53; 4.12)	**0.019**
Lysine (μg/ml)	2.81 (2.12; 3.93)	3.25 (2.41; 5.03)	2.50 (1.97; 3.95)	3.17 (2.62; 4.22)	2.76 (1.77; 3.57)	2.41 (1.99; 3.30)	**0.001**
Methionine (μg/ml)	1.51 (1.24; 1.81)	1.27 (1.03; 1.72)	1.25 (1.03; 1.49)	1.55 (1.30; 1.82)	1.69 (1.51; 1.87)	1.67 (1.49; 1.92)	**<0.001**
Phenylalanine (μg/ml)	2.96 (2.40; 3.47)	2.56(2.02; 3.35)	2.54 (1.98; 2.99)	3.22 (2.74; 3.90)	3.14 (2.58; 3.35)	3.19 (2.64; 3.58)	**0.009**
Threonine (μg/ml)	10.13 (7.62; 13.44)	8.87 (7.43; 13.58)	10.32 (6.06; 13.00)	10.03 (7.70; 13.23)	9.91 (7.75; 13.23)	10.61 (8.40; 14.34)	0.59
Tryptophan (μg/ml)	0.74 (0.59; 0.98)	0.74 (0.57; 1.35)	0.50 (0.33; 0.82)	0.71 (0.67; 0.92)	0.81 (0.69; 0.96)	0.80 (0.65; 1.00)	0.06
Valine (μg/ml)	19.21 (16.34; 22.42)	22.35 (19.21; 24.89)	20.95 (17.90; 24.29)	19.56 (16.34; 21.94)	17.84 (15.71; 19.68)	16.07 (14.05; 18.90)	**<0.001**
**Non-essential amino acids**							
Alanine (μg/ml)	25.04 (20.43; 31.41)	22.63 (18.31; 29.65)	22.91 (18.23; 26.02)	23.99 (20.88; 31.32)	29.56 (22.71; 34.07)	27.38 (23.60; 31.41)	0.23
Arginine (μg/ml)	3.36 (2.67; 4.49)	4.11 (3.07; 5.73)	3.34 (2.51; 4.51)	3.47 (2.89; 3.95)	3.10 (2.02; 3.77)	2.99 (2.40; 3.94)	0.13
Asparagine (μg/ml)	3.05 (2.15; 4.38)	2.05 (1.69; 2.89)	2.83 (2.15; 4.24)	3.57 (2.93; 5.23)	3.61 (2.65; 4.83)	3.32 (2.25; 4.37)	**<0.001**
Aspartic acid (μg/ml)	8.99 (5.99; 12.06)	9.08 (5.99; 11.29)	5.88 (4.05; 7.68)	9.35 (6.96; 12.66)	10.50 (7.32; 15.45)	11.16 (8.61; 14.77)	**0.02**
Glutamic acid (μg/ml)	216.41 (176.54; 260.39)	205.63 (151.60; 231.07)	175.84 (147.57; 217.61)	248.90 (199.16; 275.96)	238.78 (203.66; 284.42)	225.40 (200.71; 273.62)	**<0.001**
Glutamine (μg/ml)	57.20 (28.76; 83.81)	25.33 (12.63; 37.54)	39.53 (18.68; 62.24)	67.78 (42.53; 91.41)	78.74 (59.15; 101.41)	77.12 (51.38; 93.11)	**<0.001**
Glycine (μg/ml)	10.83 (9.02; 12.84)	9.53 (7.91; 11.57)	10.78 (9.12; 12.18)	10.58 (9.69; 13.08)	11.46 (9.65; 14.52)	11.68 (10.04; 13.75)	**0.002**
Serine (μg/ml)	15.30 (10.87; 19.48)	10.51 (9.26; 13.16)	12.79 (10.65; 16.45)	17.09 (14.99; 19.63)	18.46 (16.01; 22.61)	17.47 (14.19; 21.52)	**<0.001**
Taurine (μg/ml)	27.67 (21.16; 34.88)	33.80 (27.48; 44.49)	26.64 (20.50; 34.59)	27.46 (20.71; 33.63)	27.34 (20.02; 33.05)	23.43 (19.35; 31.95)	**0.002**
Tyrosine (μg/ml)	5.74 (4.29; 6.89)	6.38 (5.60; 7.92)	0.31 (0.18; 6.65)	6.20 (4.84; 6.89)	5.81 (4.99; 6.42)	4.80 (3.80; 5.91)	**<0.001**
Sum of free amino acids (μg/ml)	436.16 (362.71; 508.96)	375.89 (328.24; 448.33)	363.76 (312.26; 444.41)	471.59 (398.85; 555.73)	479.44 (416.31; 555.65)	447.51 (402.99; 539.43)	**<0.001**

*P-value on the evolution of the different items during follow-up. Median and inter-quartile range are provided. P-values in bold are statistically significant.

Interestingly, there were significant differences in total amino acids depending on HM-type in univariate analyses (*P-*value = 0.02). The median values of total amino acids were 7.8 mg/ml (IQR = 6.7 mg/ml, 9.6 mg/ml) in HM-type I, 8.5 mg/ml (IQR = 7.9 mg/ml, 9.1 mg/ml) in HM-type II, 8.5 mg/ml (IQR = 7.4 mg/ml, 11.2 mg/ml) in HM-type III, and 10.7 mg/ml (IQR = 9.6 mg/ml, 11.7 mg/ml) in HM-type IV. In sum, total amino acids were lower in HM-type I, and higher in HM-type IV, compared to the global means. Total taurine was significantly lower in HM-type I, compared to HM-type III in multilevel analyses adjusted on diet determinants and lactation period (median HM-type I taurine = 19.9 μg/ml, IQR = 15.8 μg/ml, 27.5 μg/ml, aß-coef = −10.3 *P-*value = 0.01; Supplementary Table 5). Also, total serine levels were marginally lower in HM-type I, compared to HM-type III, in multilevel analyses (aß-coef = −73.4, *P-*value = 0.05; Supplementary Data 5). Furthermore, HM-type I samples displayed significantly lower levels of alanine (ß-coef = −75.8, *P-*value = 0.03), asparagine + aspartate (ß-coef = −142.5, *P-*value = 0.04), glycine (ß-coef = −51.2, *P-*value = 0.03), phenylalanine (ß-coef = −70.8, *P-*value = 0.046), serine (ß-coef = −88.6, *P-*value = 0.03), taurine (ß-coef = −8.5, *P-*value = 0.03), and threonine (ß-coef = −84.9, *P-*value = 0.04) in univariate analyses, compared to HM-type III.

Glutamic acid + glutamine, leucine, and aspartic acid + asparagine were the amino acids present in HM in a higher proportion (median = 21.7%, IQR = 20.3%, 22.8%; median = 11.5%, IQR = 11.3%, 11.7%; and median = 11.2%, IQR = 10.9%, 11.4%, respectively; Supplementary Data 4).

The analyses of the differences in relative levels of total amino acids depending on HM-type are detailed in the Appendix B.

#### Free amino acids

Most free amino acid levels were significantly different depending on lactation period (Table 4). The median value of the sum of free amino acids decreased initially from 375.9 μg/ml week 1 after delivery (IQR = 328.2; 448.3) to 363.8 μg/ml at week 4 (IQR = 312.2; 444.4), then increased to 471.6 μg/ml (IQR = 398.9; 555.7) at week 11, and 479.4 μg/ml (IQR = 403.0; 555.7) at week 18, and then diminished again to 447.5 (IQR = 416.3; 539.4) at week 25. The concentration of free threonine, tryptophan, alanine, and arginine remained stable within follow-up visits whereas the rest of amino acids varied significantly depending on the lactation period. The box-plots of free amino acid distribution as absolute value are presented in Supplementary Data 6.

There were significant differences in the concentration of free amino acids within the different HM-types. The sum of all free amino acids was significantly lower in HM-type II, adjusted on lactation period and diet determinants, and compared to HM-type III (median = 376.0 μg/ml, IQR = 325.4 μg/ml, 444.5 μg/ml, aß-coef = −83.3, *P*-value = 0.002). In multivariate analyses, compared to HM-type III, HM-type I had significantly lower levels of free tyrosine (median = 5.5 μg/ml, IQR = 3.8 μg/ml, 6.5 μg/ml, aß-coef = −1.6, *P*-value = 0.02); HM-type II had borderline significantly lower levels of free glutamic acid (median = 195.5 μg/ml, IQR = 151.0 μg/ml, 224.7 μg/ml, aß-coef = −25.4, *P*-value = 0.05), and significantly lower levels of glutamine (median = 31.6 μg/ml, IQR = 22.1 μg/ml, 62.7 μg/ml, aß-coef = −25.4, *P*-value = 0.03), serine (median = 13.7 μg/ml, IQR = 10.4 μg/ml, 16.4 μg/ml, aß-coef = −4.3, *P*-value = 0.01), and tyrosine (median = 5.4 μg/ml, IQR = 3.9 μg/ml, 7.0 μg/ml, aß-coef = −1.5, *P*-value = 0.046); and HM type IV had only significantly lower levels of free tyrosine (median = 5.0 μg/ml, IQR = 3.7 μg/ml, 5.7 μg/ml, aß-coef = −2.3, *P*-value = 0.01). These multilevel models are presented in Supplementary Data 7.

Free glutamic acid accounted for half of the concentration of free amino acids during the entire follow-up (median = 50.2%, IQR = 47.0%, 53.4%, Table 4). Glutamic acid, alanine, methionine, phenylalanine, threonine, and tryptophan were the only amino acids whose proportions did not vary significantly during follow-up (Table 4). The analyses of relative levels of free amino acids are presented in Appendix C.

### Retinol

**Figure 4:**
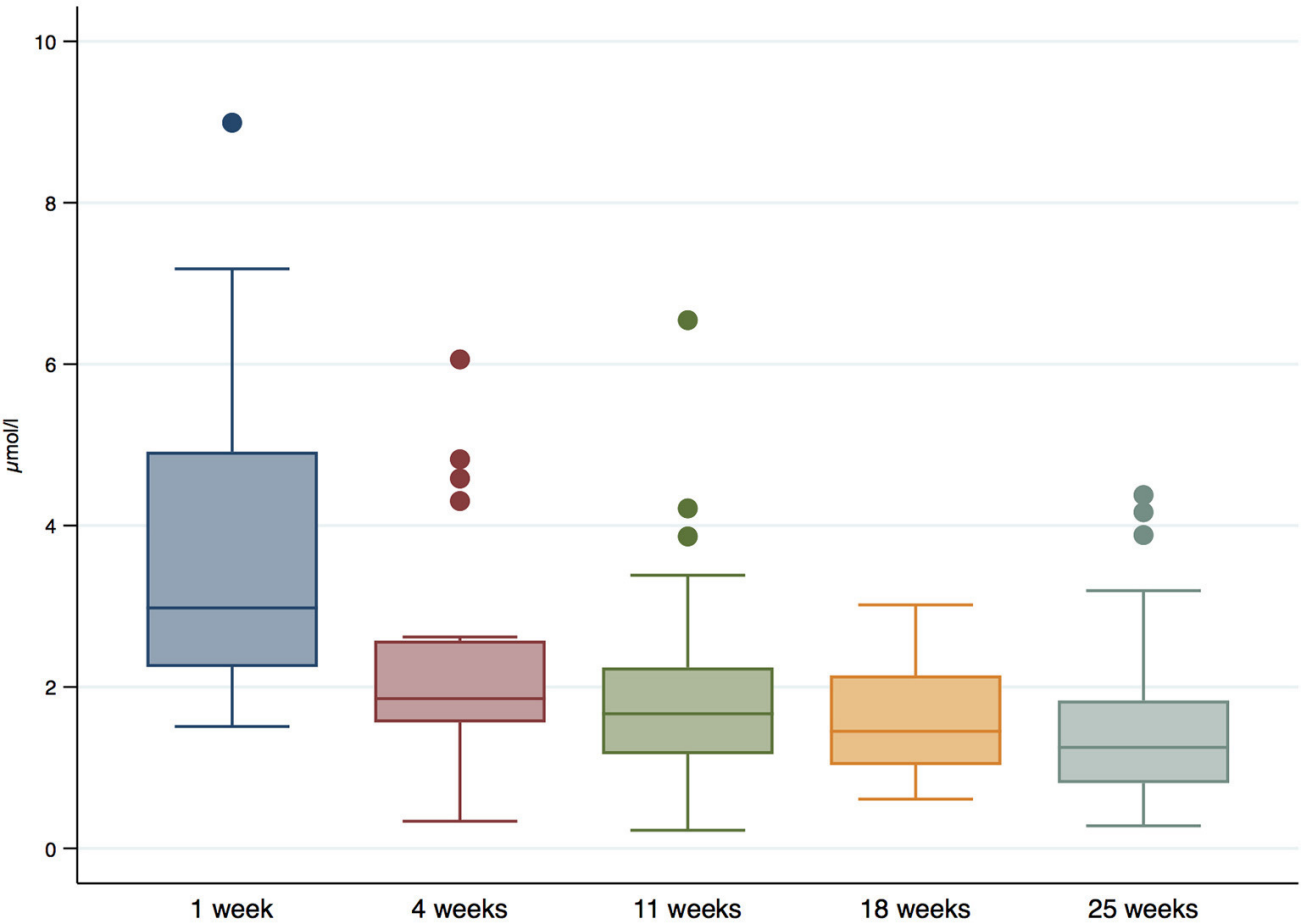
Retinol levels (μmol/l) in HM during follow-up.

The median retinol levels in HM were 1.9 μmol/l (IQR = 1.3 μmol/l, 2.7 μmol/l), and they decreased significantly during follow-up (*P*-value < 0.001). Concretely, the median values decreased from 2.9 μg/ml (IQR = 2.2, 4.4 μg/ml) 1 week after birth, to 1.9 μg/ml (IQR = 1.5, 2.4 μg/ml) at 4 weeks, 1.8 μg/ml (IQR = 1.2, 2.3 μg/ml) at 11 weeks, 1.5 μg/ml (IQR = 0.8, 2.1 μg/ml) at 18 weeks, and 1.3 μg/ml (IQR = 0.9, 1.9 μg/ml) at 25 weeks. The distribution of retinol in HM during follow-up is shown in Figure 4.

## Discussion

The present study displays meaningful characteristics in the nutritional composition of HM among Sub-Saharan African mothers, an understudied population, where health and nutritional challenges are very large. First, HMOs, fatty acids, and amino acid levels differed largely from Western cohorts. Second, there was a rare predominance of HM-type III among women in the MITICA cohort, with 44% of women with HM-type III followed by 28% to HM-type I. This is quite different from human milk type distributions found e.g., across Europe, China and in Brazil where HM-type I is by far the most abundant type (39, 44–46). Third, significant differences in HMOs, fatty acids, and amino acids (total and free) were observed across lactational periods among the different HM-types.

While the preponderance of HM-type III among Central-African women is rare, the rate of Secretor + women is similar to the ones found in Kenya (72%) (47) or Malawi, and slightly higher than the ones found in South Africa or the Gambia (48). A review including 57 peer-reviewed articles gathering evidence from 31 countries worldwide reported that 70% of the women of these studies displayed the HM-type I phenotype, 20% the HM-type II phenotype, 9% the HM-type III phenotype, and 1% the HM-type IV phenotype (49). There is evidence that Secretor + HM is associated with reduced incidence and severity of diarrhea caused by *Campylobacter jejuni* and enteropathogenic *Escherichia coli* (50, 51), while Secretor-HM is associated with reduced incidence of norovirus infection (52, 53), and it is enriched in HMOs which bind to *Helicobacter pylori* (54).

In our cohort, HMO levels appeared to be lower compared to HMO levels of Swedish (55), German (39, 56), Italian (57, 58), North-American (59), Brazilian (45), Japanese (60, 61), Samoan (62), Kenyan (47), and Burkinabe (63) women (for Burkinabe women, only 2'-FL and LNFP I were compared). There were few exceptions to these generalized lower HMO levels among Central-African women. In the Swedish study (55), women had lower levels of LNT and 3-FL, compared to our study, but the HM analyzed in this study was colostrum. Women in one of the Italian cohorts (57), and in the Brazilian cohort (45) had similar 3'-SL levels than women in our cohort. Also, compared to our cohort, the Samoan women (62) had lower levels of 2'-FL, and similar levels of LNDFH-I. Compared to Central African women, the women in the Japanese study on colostrum (60) had similar levels of 2'-FL and 3-FL, and women in the other Japanese study, including also colostrum, had lower levels of LNFP III and LNT (61). In the Brazilian and in the North-American studies (45, 59), LNT levels were lower, compared to Central-African women. In the most recent study among German women, 2'-FL, 3'-SL, LNFP I, and LNFP V values were similar to the Central Africans'. Further, LNT and LNnT were lower, compared to our study. In the Kenyan study (47), HM analyses were not performed at concrete time-points but at two different moments, 2 months apart. The comparison of HMOs can be misleading, as the time of lactation was not standardized in the Kenyan study, and HMOs vary significantly across the different lactation stages. In any case, 2'-FL and LNnT absolute and relative levels were higher in our cohort compared to the Kenyan. On the contrary, 3-FL, 3'-SL, 6'-SL, LNFP I, LNFP II, and LNFP III absolute and relative concentrations were lower in our cohort, compared to the Kenyan. While LNT absolute levels were similar in both cohorts, LNT relative levels were higher in our cohort, compared to the Kenyan.

In a recent review gathering evidence from 57 peer-review articles from 31 countries all median HMOs levels were higher, compared to the HMOs found among these Central-African women (49). Only median 3'-SL and LNT values in transitional milk were lower in the review, compared to our cohort. Median 2'-FL concentration in transitional and late HM and median LNT levels in mature and late HM were similar in our cohort than in this review. Very similar results were reported in another recent review on HMO data gathered worldwide between 1999 and 2019 (64). Overall HMO levels were lower in our cohort, compared to HMO concentration in HM from women from Germany (56), Spain (65), China (44, 66, 67), Malaysia (66), Unite Arab Emirates (UAE) (68), Singapore (7), Samoa (69), USA (70, 71), Canada (72), and other countries (73, 74). However, median 2'-FL levels in transitional and late HM were higher in our cohort, compared to 2'-FL HM levels among Samoan (69), American (70), Spanish (65), Chinese (44, 66, 67), UAE (68), and Malaysian (66) women. In parallel, median LNT levels in transitional and mature HM were also higher in our cohort, compared to LNT concentration in HM from American (70, 71), Chinese (44, 67), Spanish (65), Singaporean (7), and other women worldwide (56, 74). Certain studies also showed lower levels of 3'-SL, mainly in transitional HM (44, 66, 69, 70). In conclusion, HMO levels were lower in Central-African women, compared to other cohorts. However, LNT levels were higher than in some other cohorts. We refer here to the different HMO levels individually, as we have not analyzed all existing HMO structures. Indeed, the comparison of total HMO levels in different cohorts is biased by the heterogeneity of the HMOs measured.

Compared to HM-type III (the most abundant HM-type), LNT was significantly more abundant in HM-type II and HM-type IV, which is coherent with previous HM cohort analyses (39, 75). In contrast, we found that LNFP V was higher in HM-type I and HM-type II compared to HM-type III in contrast with other cohorts, where LNFP V was found to be more abundant in HM-type II and IV (39).

As found in other HM cohort analyses, total HMO levels decreased significantly during the first 6 months of lactation (48, 49, 56, 64, 75–77). 3-FL was the only HMO found to significantly increase during the first 6 month after birth, which is in line with findings in European and Asian HM cohorts (39, 66, 67, 75). In contrast with most HMOs which declined, DFL, 4'-GL, and LNDFH II + LNnDFH II remained stable during the 6 months post-partum in the MITICA study. DFL was also found to remain constant over lactation up to 6 month in a European cohort, such as in the German Ulm SPATZ Health study (39). In contrast to Europe (39, 75) and Asia (67), we observed that 3'-SL was declining during the first 6 months after birth. In a previous study conducted in Gambia, 3'-SL relative concentration was found to be positively associated with infant growth at 20 weeks (measured by weight-for-age *Z*-score) (78). Further research is needed to assess whether levels of individual or total HMOs may be associated to health outcomes in children. Indeed, the low levels of HMOs in this Central-African cohort, alongside its different evolution across lactation period might entail meaningful variations in the microbiota colonization process. Also, HMOs have important immunomodulatory, anti-biofilm and antiadhesive properties. The consequences of low HMO levels on the infant immunity and infections in the context of a high infectious diseases burden need further analyses.

In contrast to HMOs, lactose levels were not significantly different in our cohort, compared to other studies (39, 56). In parallel, lactose concentrations increased significantly over lactation, as seen in other HM cohorts where lactose peaks at 6 months and then decreases again until 12 months (39).

The lipid fraction of HM represents the main source of energy intake for the exclusively breastfed infant during the first 6 months of life, i.e., 44% of energy supply on average (9). With the exception of two studies of HM in European women (9, 79) and the 6th week post-partum results in another study (80), total fatty acid concentration in our cohort was lower, compared to German (80, 81), Italian (82), Chinese (83) HM donors. In a recent American study on HM samples taken 6–18 months after delivery among 54 women, absolute levels of MUFA, PUFA, ARA, and DHA levels were also lower than in HM of the Central-African women taken 6 months after delivery (84). However, SFA levels were similar in both cohorts. The relative amounts of SFAs and MUFAs in HM of Central-African women also differed notably from Italian (85), Icelander (86), and Chinese women (83, 87), in addition to other pooled analyses (9, 88). More precisely, the percentage of SFAs in our cohort was higher, compared to other cohorts. In parallel, relative levels of MUFAs and PUFAs were lower—MUFAs much lower -, compared to other cohorts. A recent cross-sectional Chinese study gathering samples of 64 women 40–100 days after delivery displayed similar MUFA and DHA relative levels in HM, compared to our cohort. In parallel to other cohorts, SFA relative levels were also higher in our cohort and PUFAs, LA, ARA, Omega-3, and ALA were lower, compared to these Chinese women. Indeed, an important recent review including 186 different HM studies showed that HM from Western countries had significantly higher concentrations of MUFAs and lower concentrations of PUFAs, total Omega-6 PUFA, compared with those from non-Western countries (89). In this review median SFA relative levels are also lower, and median PUFA relative levels were also higher than in our cohort of Central-African women. This might be explained by a difference in maternal fatty-acid intake between Central-African and other countries (43). Lipid composition in HM is substantially influenced by maternal diet (9), and in our study, the consumption of meat, poultry, and fish was significantly associated with higher levels of fatty acids, while a high food insecurity index was significantly correlated with lower levels of fatty acids (43).

In European cohorts, total lipid content in HM is increasing over lactation, which is attributed to a higher energy requirement during infant growth (80, 81). In the MITICA cohort, we observed that total fatty acid level in HM remained constant over time during the first 6 months of life, which is in contrast to European HM composition. Whether the lack of total fatty acids increase induces a default of energy intake for the infant and then could have negative repercussion on child growth remains to be addressed. DHA and ARA significantly decreased over lactation period in MITICA study, a finding in line with many other cohorts (83, 90), although DHA and ARA in HM were also lower in our cohort (91). DHA and ARA in MITICA study represented each 0.33% of total fatty acids, which is only half of the DHA & ARA relative amounts (0.7%) found in HM of rural Tanzanian women with high fish intake (92). Notably, we observed that the proportion of some fatty acids was different between HM-types, with relative levels of Ω-3 PUFAs being higher in HM-types I and II, compared to HM-type III and IV.

Total amino acid composition in HM was also significantly lower among Central-African mothers, compared to Chinese women (93, 94), and other 3,774 women from 83 studies included in a review (10). As for fatty acids, these differences in TAAs increase in the course of lactation. Precisely, both protein-bound amino acids and free amino acids, strongly decreased from week 1 to 3 months of lactation in the MITICA study with much smaller decline until 6 months. This TAA decline, which was not amino acid specific, has been observed consistently in HM cohorts from other geographies and correlates with the protein content decrease in HM during lactation stage (10, 95). The TAA and protein content decrease in HM has been shown to correspond to the protein requirement of the growing infant and might prevent overconsumption of proteins when the infant starts to drink increased HM volumes (96). Notably, a systematic review previously found that total tyrosine mean value was much higher in HM from Africa (86 mg/100 ml) than in HM from Asia, Europe, or North America (close to 55 mg/100 ml) (10). In MITICA, we found the opposite result, as total tyrosine global mean value was 30 mg/100 ml, so almost half less than the mean world value and three time less than the previous African data (10). This might be linked to the fact that those African data came from much older studies (1979 in Ivory Coast and 1977 in Ethiopia) using different analytical methods. Total arginine and isoleucine in HM from MITICA were also lower in absolute values than the values obtained in the systematic review (10). Interestingly, HM-type I contained lower levels of TAA. This could be explained by the lower proportion of girls in the HM-type I group, as higher TAA content has previously been associated with female gender in a Dutch cohort (64). Whether these differences might translate into differential infant growth and development requires further attention.

In addition to protein-associated amino acids, free amino acids represent 5–10% of TAA (10, 95). Growing interest in HM FAAs is due to the fluctuation of FAA levels over lactation in an amino acid specific manner which suggests the existence of a secretory regulation mechanism. Indeed, FAAs might have a functional role in infant development (97). FAAs are more rapidly digested and appear sooner in the circulation than protein-associated amino acids and are recognized by specific receptors at the surface of numerous cells. The most abundant FAAs found in MITICA HM were glutamic acid, representing 50% of all FAAs globally, followed by glutamine (13%), taurine (7%), and alanine (6%). These findings are consistent with others HM cohorts (10, 95). As found in another study, free glutamic acid, glutamine, aspartic acid, and serine most strongly increased between months 1 and 3 of lactation (95). Interestingly, glutamine increased both in absolute and in relative values over lactation, from 7% at week 1 until 16% at 6 months. Free glutamic acid and free glutamine have been extensively studied, and it is well-established that they provide energy to intestinal epithelial cells, inducing their proliferation and reinforcing intestinal barrier function (98). Free glutamic acid and glutamine might act on immune cells, promoting TH1 vs. TH2 responses and exerting anti-inflammatory effects.

This is part of the mechanisms by which breastfeeding has a protective effect against neonatal allergies and infections (98). Interestingly, as seen with TAA, FAAs differed depending on HM-type. Concretely, HM-type II contained significantly less specific FAA levels than HM-type III, including glutamic acid, glutamine, serine, and tyrosine. The consequences of lower amounts of those FAAs on infant development are not clear yet.

Finally, we should consider the setting in which the MITICA study was conducted. The main objective of the study was to analyze the mechanisms of mother-infant transmission of gut dysbiosis associated with environmental enteric dysfunction. Around 35% of the women were defined as undernourished, over 60% had vitamin A deficiency, and almost 80% vitamin C hypovitaminosis at inclusion. These nutritional deficiencies might reflect dietary shortages or malabsorption conditions resulting in impaired nutritional status of the mothers with possible consequences for HM composition. Indeed, food insecurity and maternal diet had a significant influence on the HM composition of this cohort (43). Precisely, a high index of food insecurity was associated with lower levels of fatty-acids, amino acids, retinol, and the majority of the HMO measured in HM. This is in agreement with the results found by Vinjamuri et al. (48), who found that HM from mothers living in countries with a low GDP per capita had lower HMO levels in 2,000 HM samples from over 1,000 mothers in geographically diverse countries.

The moderate number of women included in the cohort constitutes an important limitation of this study. The strengths of this study rely on the follow-up from birth to 6 months, with HM samples taken very regularly. Also, the HM samples taken at 1 week after birth allow the comparison between transient milk (at 1 week) with mature milk. Recent data on HM composition from African countries is scarce (47, 99), and our study displays similarities with results from other geographic locations, but also clear differences, such as the predominance of HM-type III. Significant correlations were found between HM nutritional composition and specific HM-type, which are novel and warrant further studies with more HM samples to analyze the association of HM-type distribution and their nutritional pattern, with infant development. HM composition data from Africa remain too rare and are critical to better characterize the impact of HM on infant health and development in the region with the highest burden of infections and undernutrition.

## Conclusion

In the MITICA study, human milk of Central-African women displayed very specific nutrient patterns, partly deviating from Western cohorts. Furthermore, food insecurity had a significant effect on the HM nutrient concentration. This suggests that the effect of food insecurity might be more important than previously postulated. For example, we revealed decreased HMO levels which might translate into differences in the infant gut bacterial colonization process. This might be an interesting aspect for future research. Furthermore, relative levels of SFAs were higher and MUFA levels were lower compared to other cohorts. Also, total fatty-acid and amino-acid concentrations were comparatively low, and these differences increased with infant age. In contrast to observations in other studies, fatty acid levels did not increase during the course of lactation, and some of them decreased significantly during follow-up. In which manner maternal nutritional status, diet, and food insecurity might be responsible for these differences and effects on infant growth warrants further research. Finally, also the clearly observed HM-type dependent variations in the nutrient profiles deserve further investigation. The rare predominance of HM-type III (44% of the mothers) and its specific nutritional profile (lower levels of Ω-3 PUFAs, LNFP V, LNT, methionine, and higher specific free amino acids) are also interesting characteristics of this cohort. Indeed, in Central-Africa nutritional deficiencies overlap with infectious diseases, also among lactating women. In conclusion, our results on HM composition of Central-African women suggest that food insecurity together with infectious disease burdens in the region might influence HM composition. Further complementary research on the effect of maternal undernourishment on HM composition is essential. Overall, our results plead for fostering breastfeeding in areas with fragile food access in parallel to increasing food security among lactating women.

## Data availability statement

The raw data supporting the conclusions of this article are available on request from the corresponding authors. The data are not publicly available due to privacy or ethical restrictions.

## Ethics statement

MITICA study was approved by the Ethics Committee of the Faculty of Sciences of Bangui (Approval number: 9/UB/FACSS/CSVPR/17), the Ministry of Health of the Central African Republic (Approval number: 189/MSP/DIRCAB/DGPGHV/DGEHU), and the Institutional Review Board of the Institut Pasteur in France (Approval number: 2016-09/IRB). Written informed consent to participate in this study was provided by the participants' legal guardian/next of kin.

## Author contributions

VM-A, BS, RB-S, and PS designed the MITICA study protocol and the research analyses. VM-A, J-CK, DM-B, and J-BK conducted the study. YN and GN performed the blood laboratory analyses. MM and BS supervised the Human Milk nutrient composition laboratory analyses. VM-A performed the statistical analyses. VM-A, SE, MM, BS, PS, and RB-S critically analyzed the data and conceptualized the manuscript. VM-A and RB-S drafted the manuscript. All authors have read and agreed to the published version of the manuscript.

## Funding

This project was funded by the PTR (Programmes Transversaux de Recherche) grant 91-17 from the Institut Pasteur Paris and the LabEx IBEID (ANR-16-COV-005). Also, VM-A was supported by this postdoctoral fellowship from the LabEx IBEID.

## Conflict of interest

SE, MM, BS, and RB-S were Danone Nutricia Research employees. The remaining authors declare that the research was conducted in the absence of any commercial or financial relationships that could be construed as a potential conflict of interest.

## Publisher's note

All claims expressed in this article are solely those of the authors and do not necessarily represent those of their affiliated organizations, or those of the publisher, the editors and the reviewers. Any product that may be evaluated in this article, or claim that may be made by its manufacturer, is not guaranteed or endorsed by the publisher.
