# Supplementary material for: Human milk nutritional composition across lactational stages in Central Africa
Source: Front Nutr. 2022 Nov 16;9:1033005. doi: 10.3389/fnut.2022.1033005 (PMC9709887; doi:10.3389/fnut.2022.1033005)
Supplement: Supplementary file 1 [file Data_Sheet_1.docx]

Supplementary Material


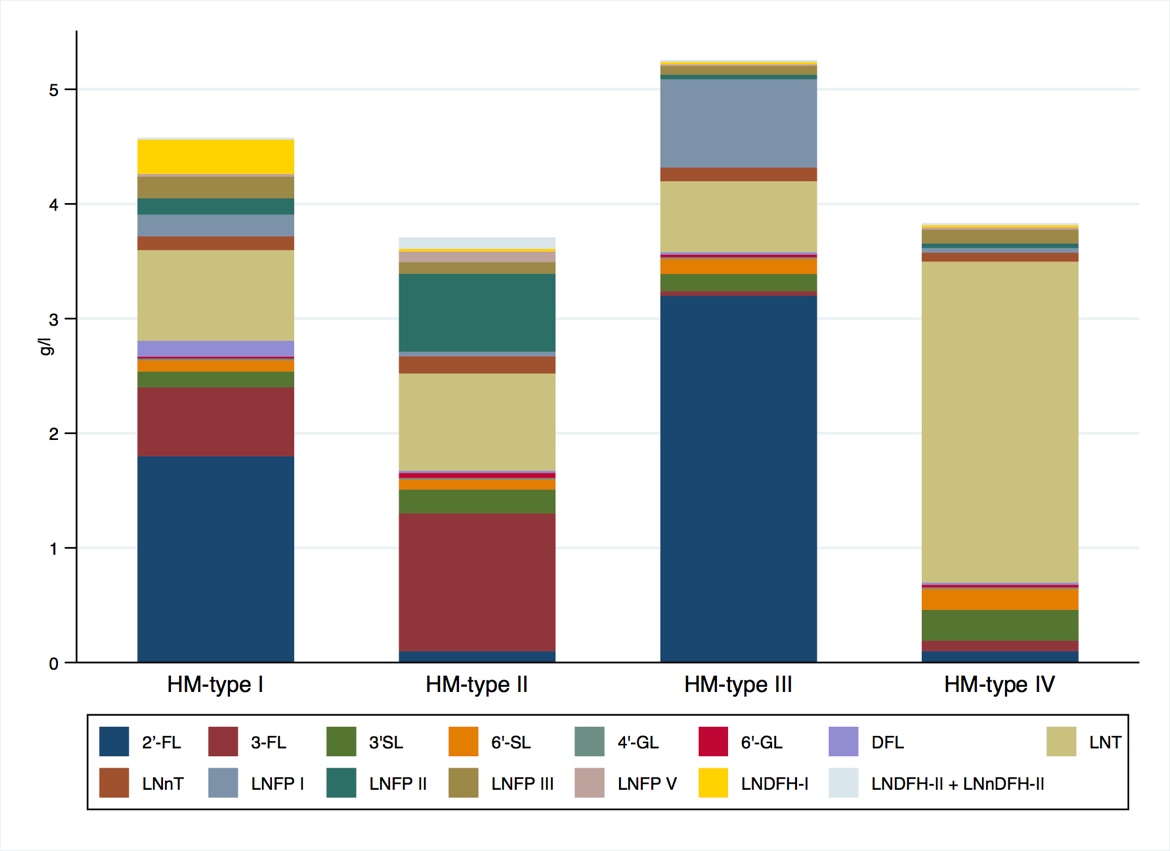
**Figure S1- Distribution of HMOs among HM-types (absolute values in g/l) during all time points of follow-up**

**Figure S2 - Distribution of fatty acids (absolute values) during lactation stages**


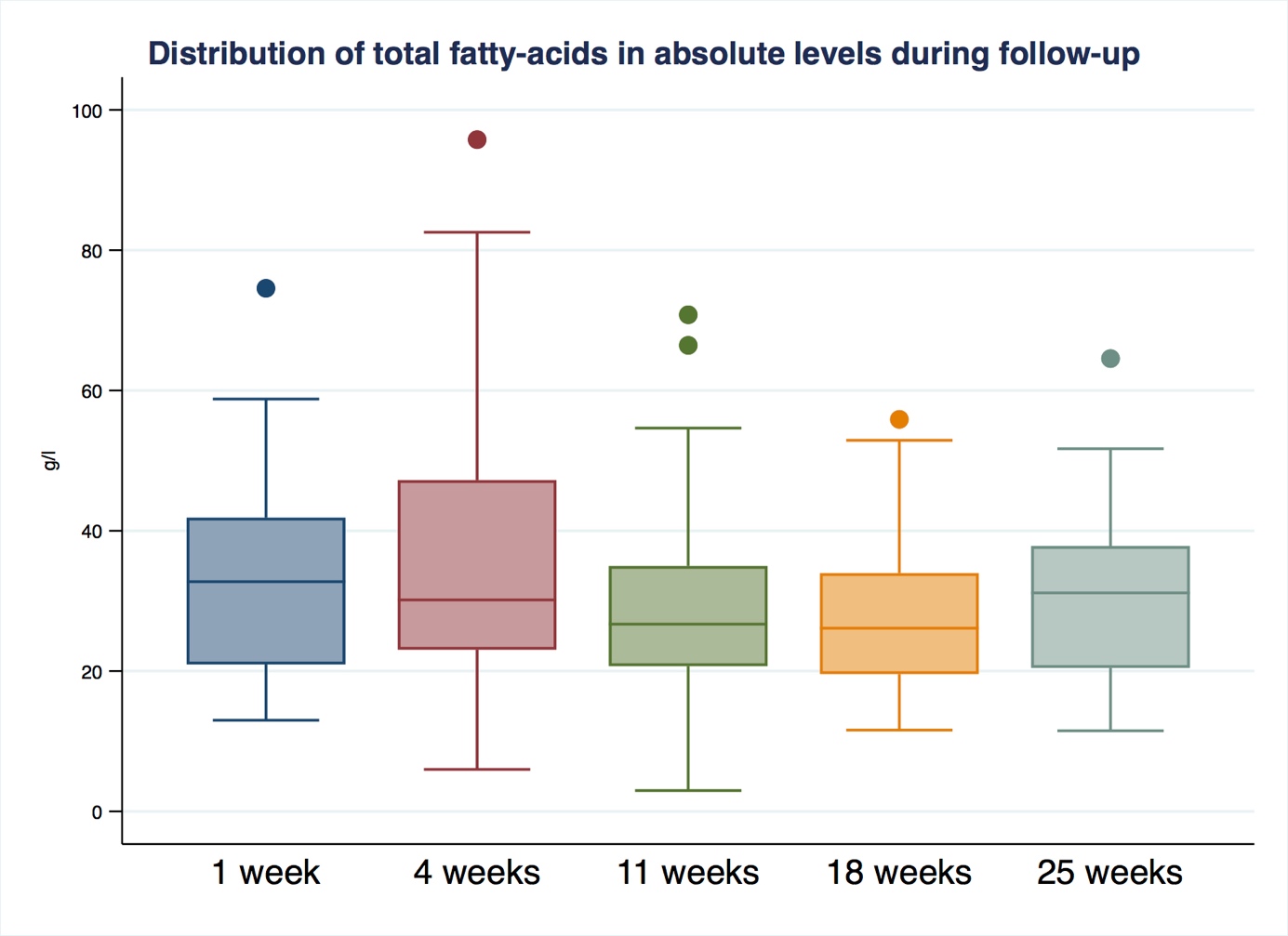


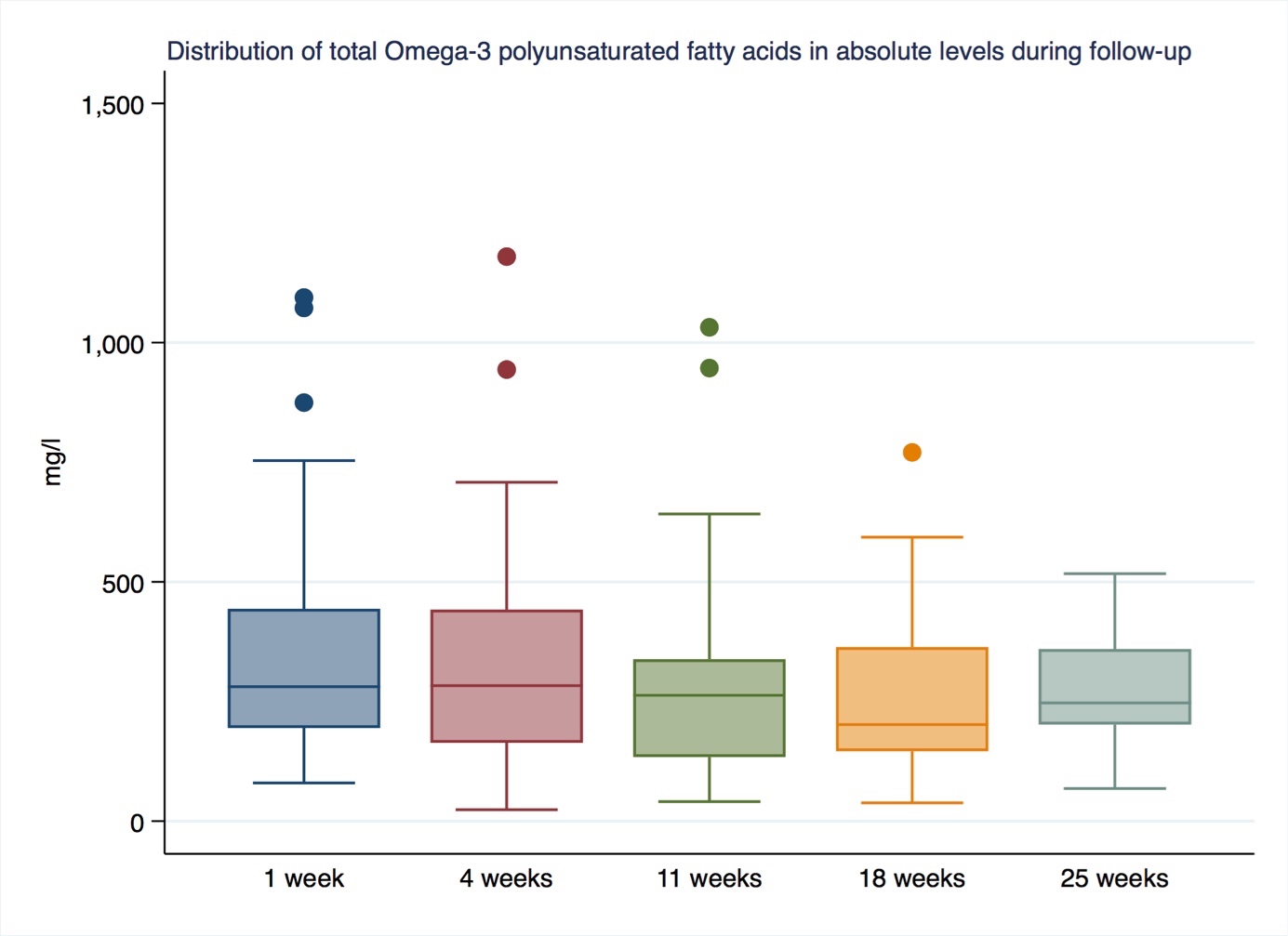

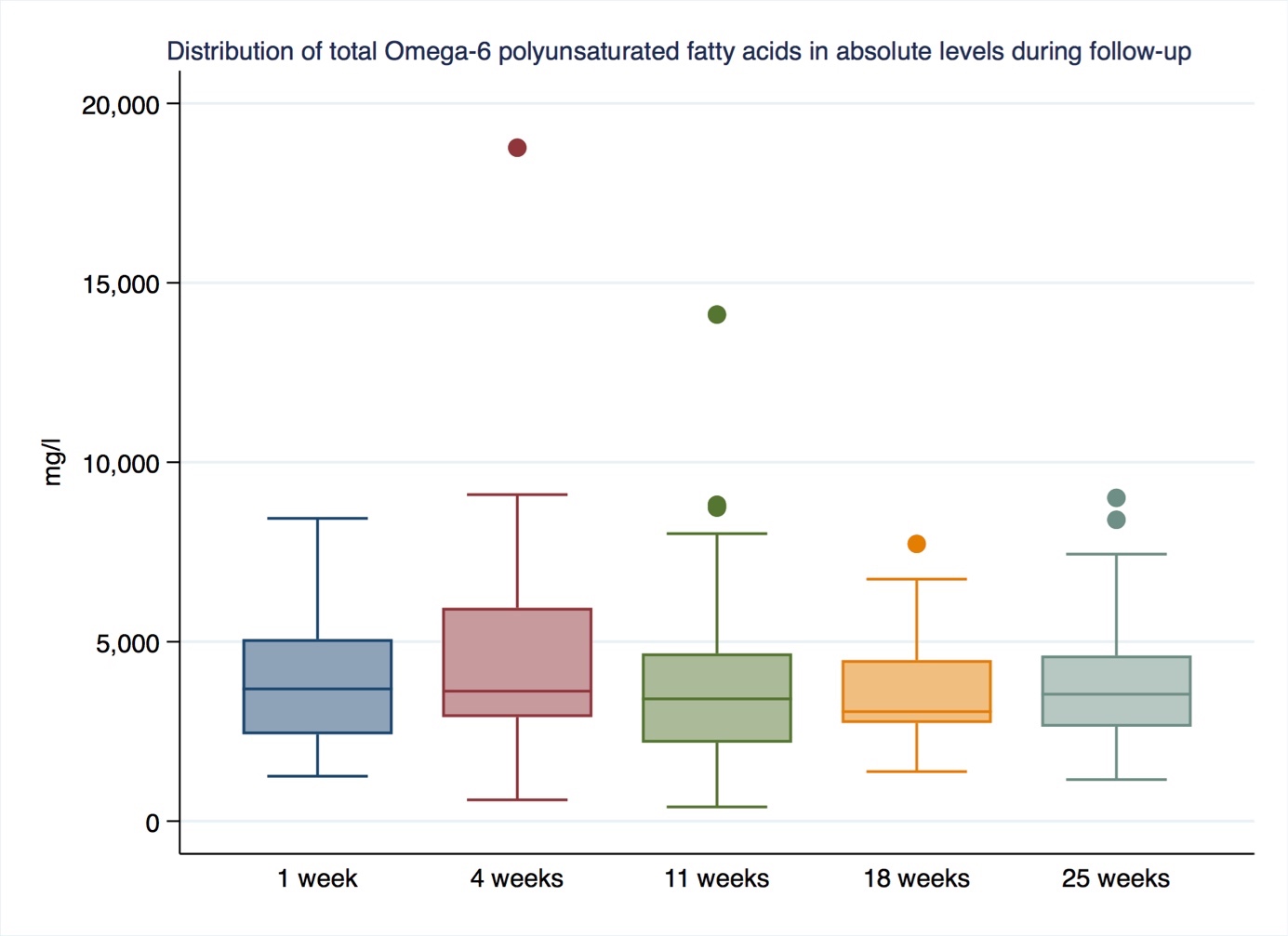

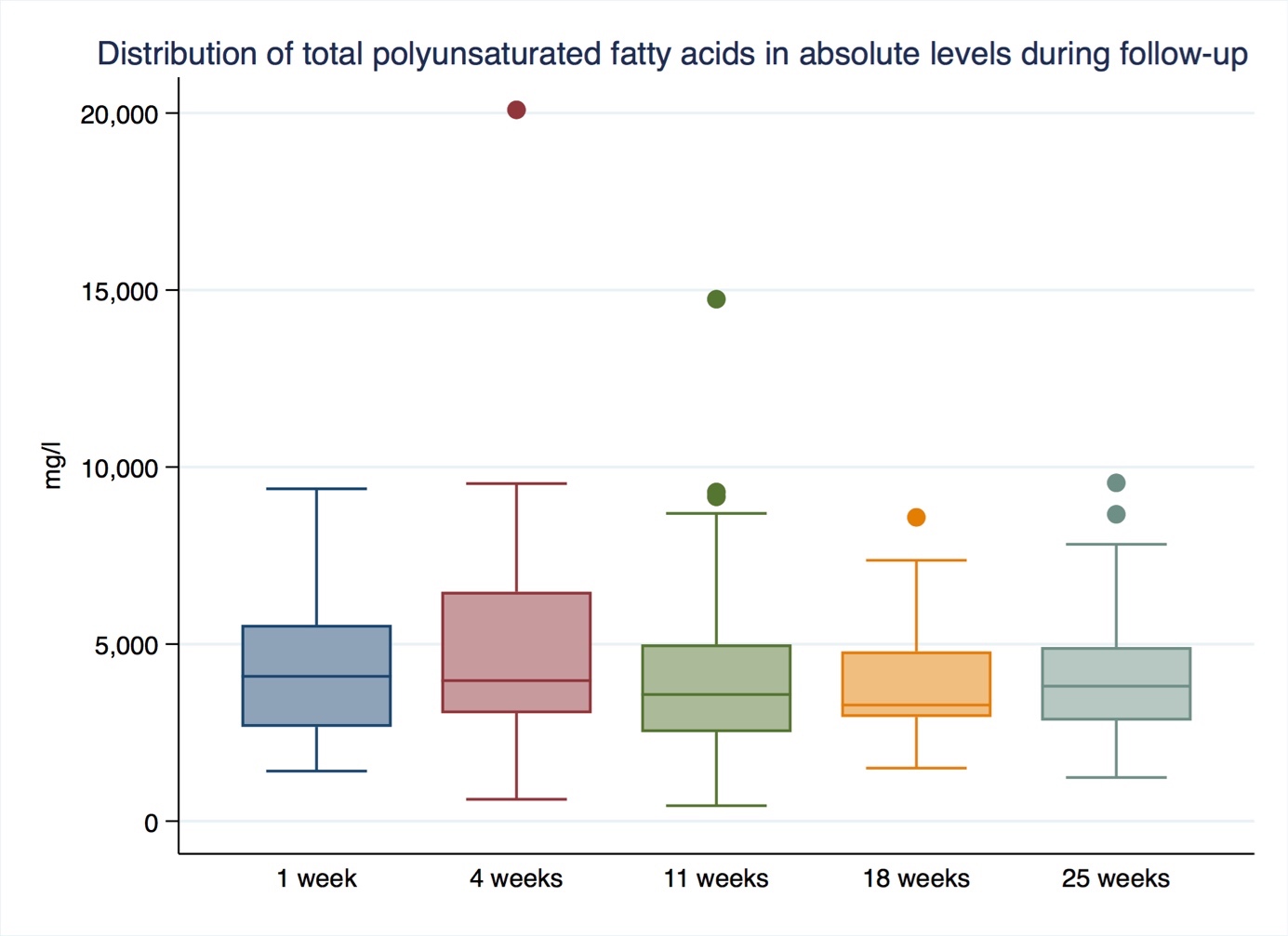


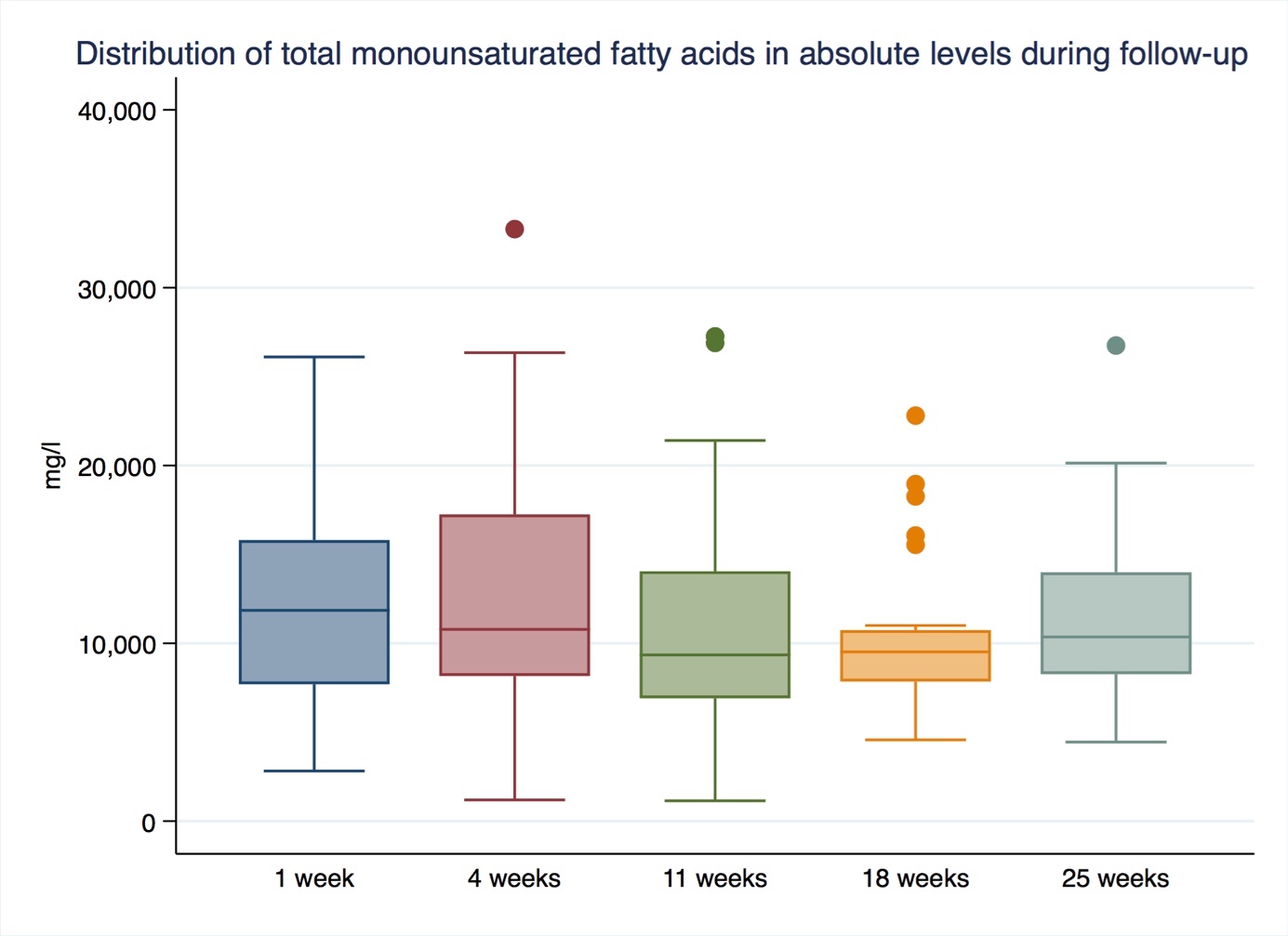


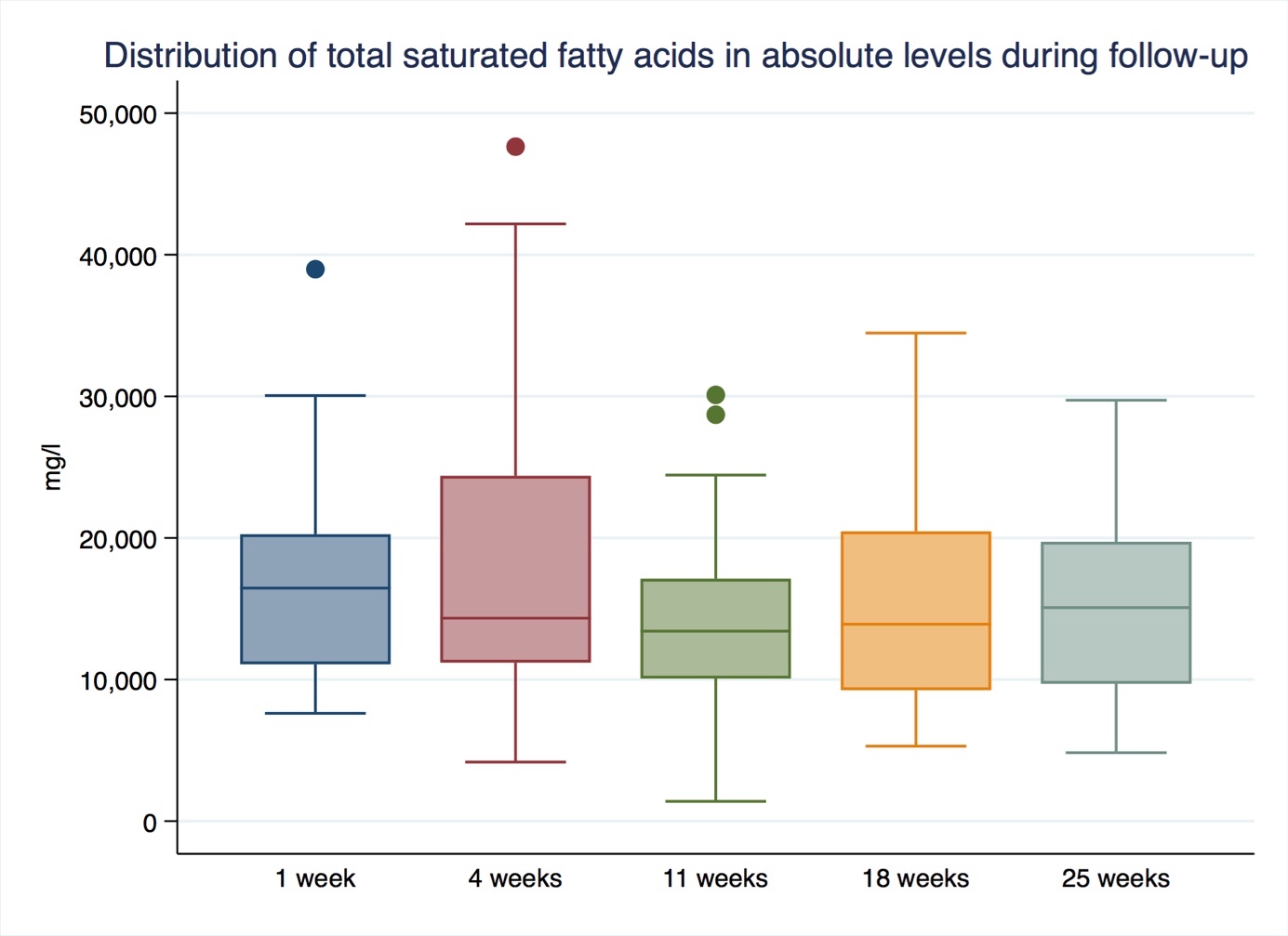

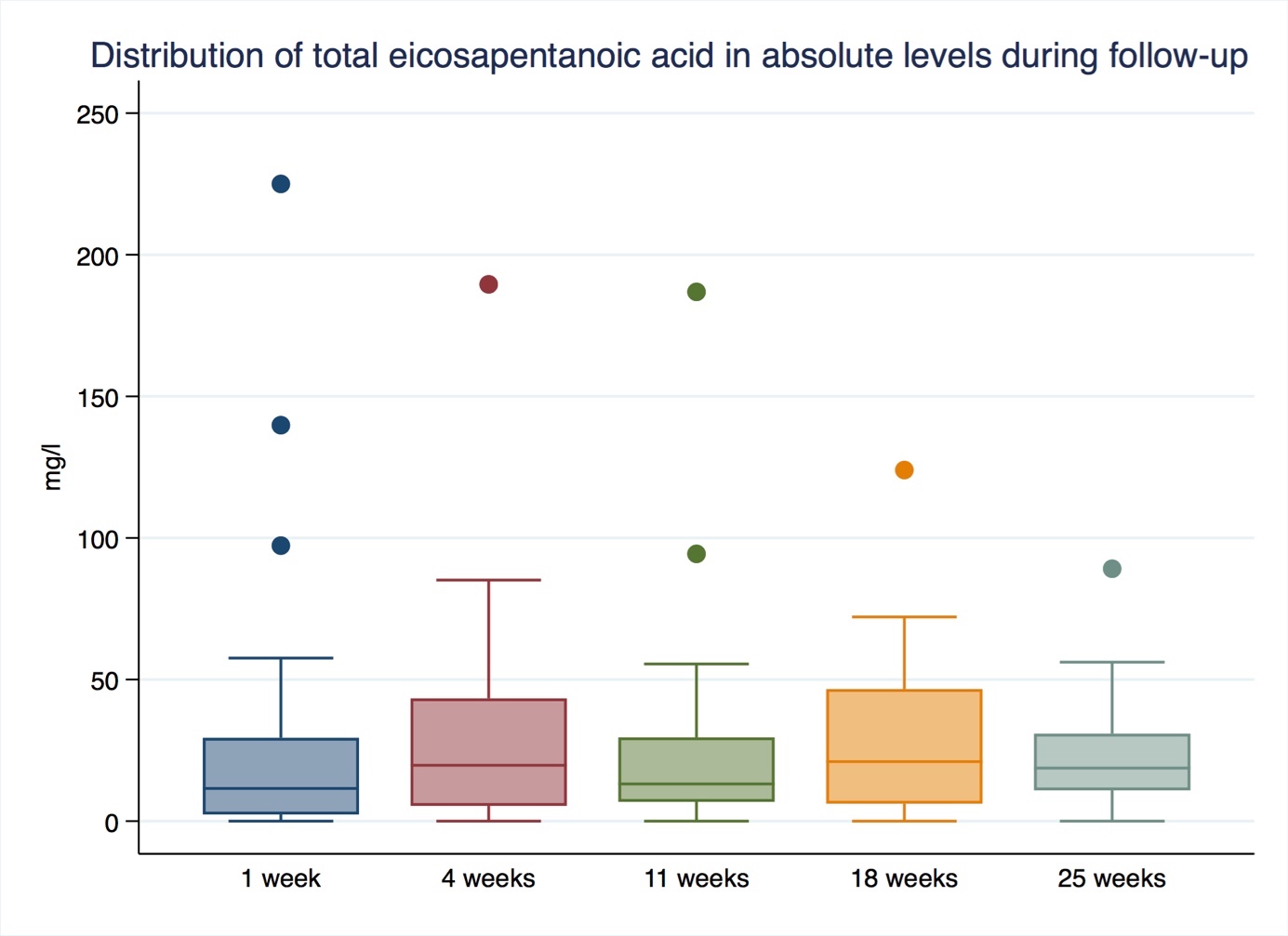

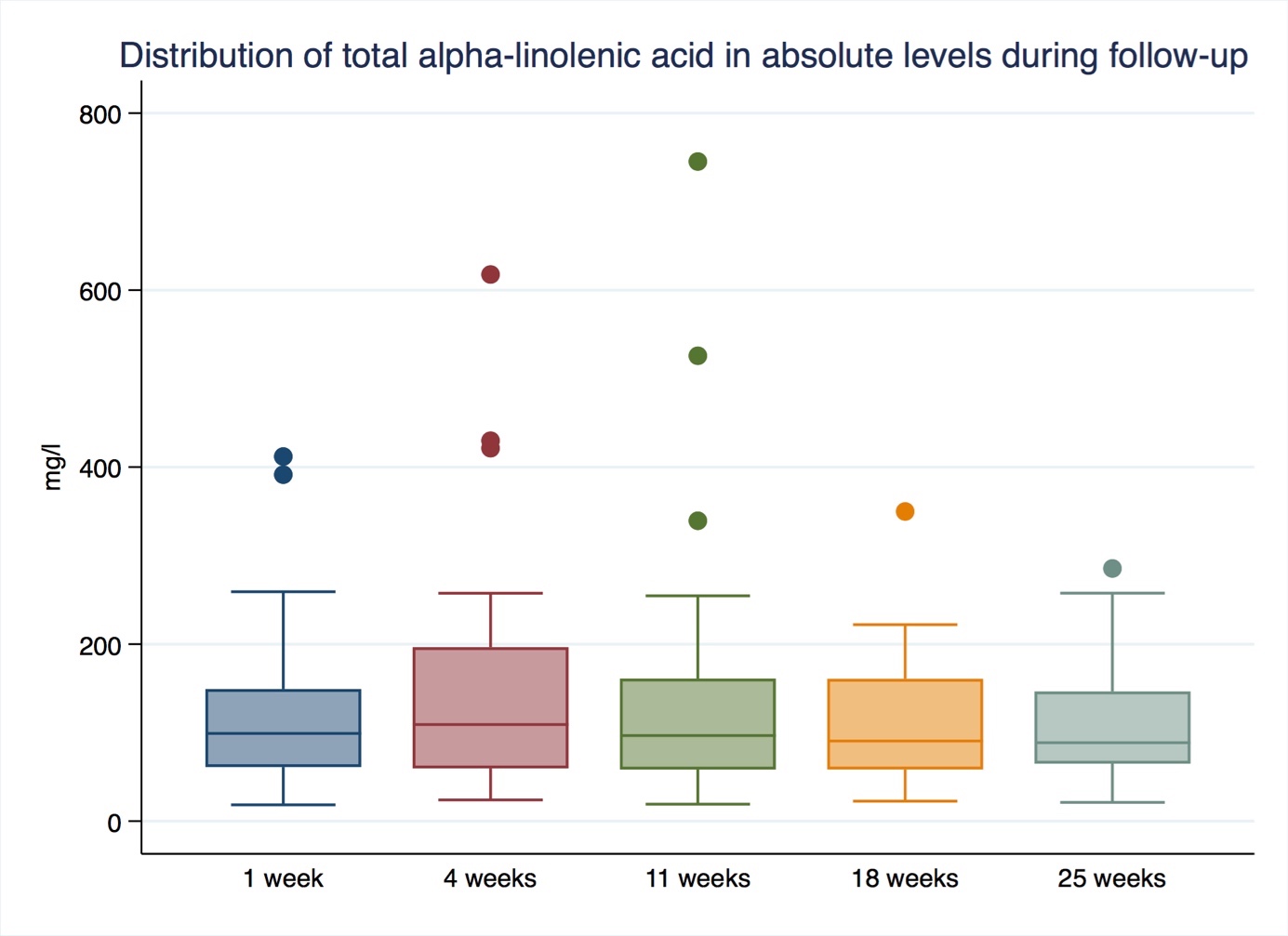

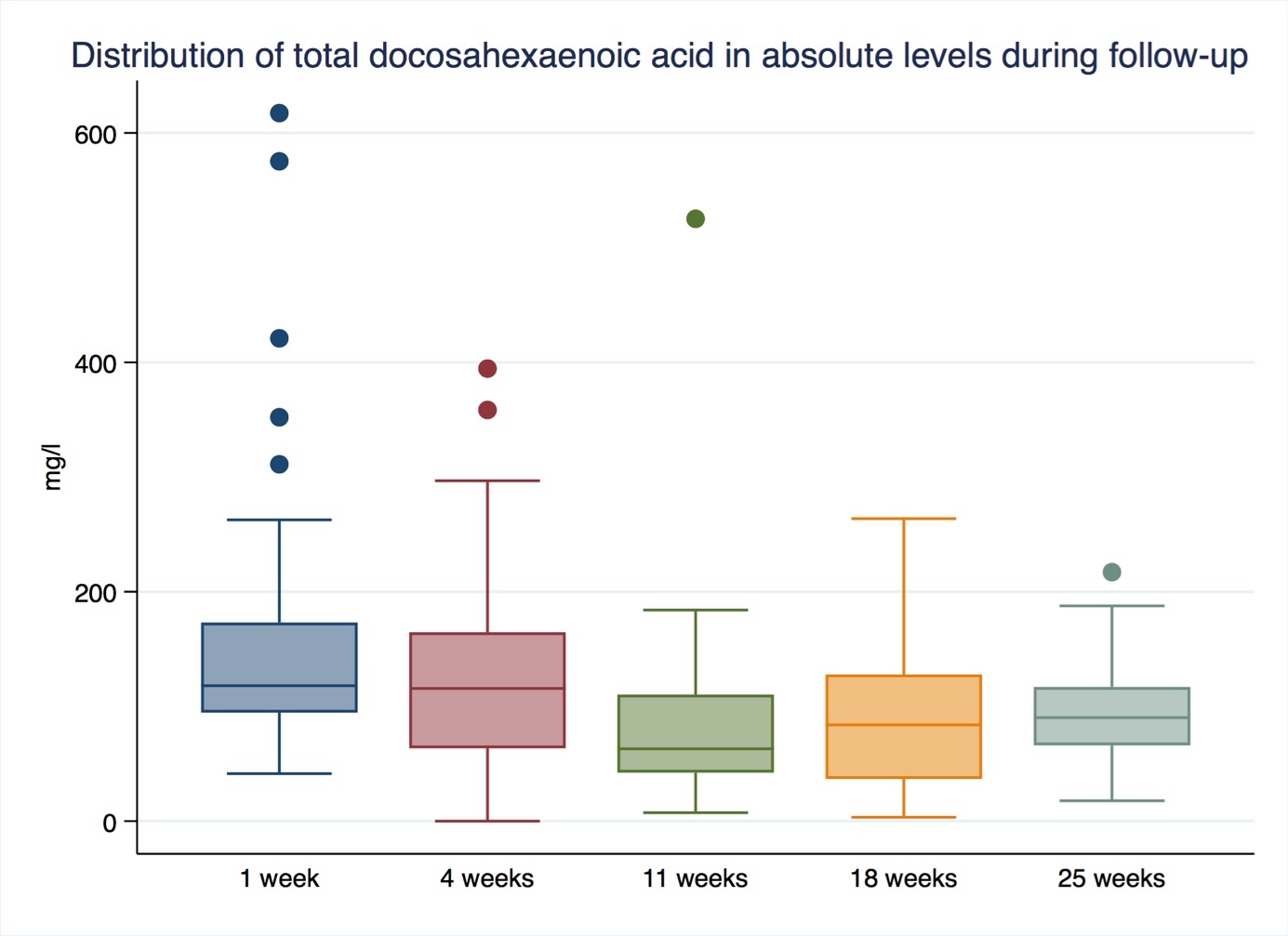

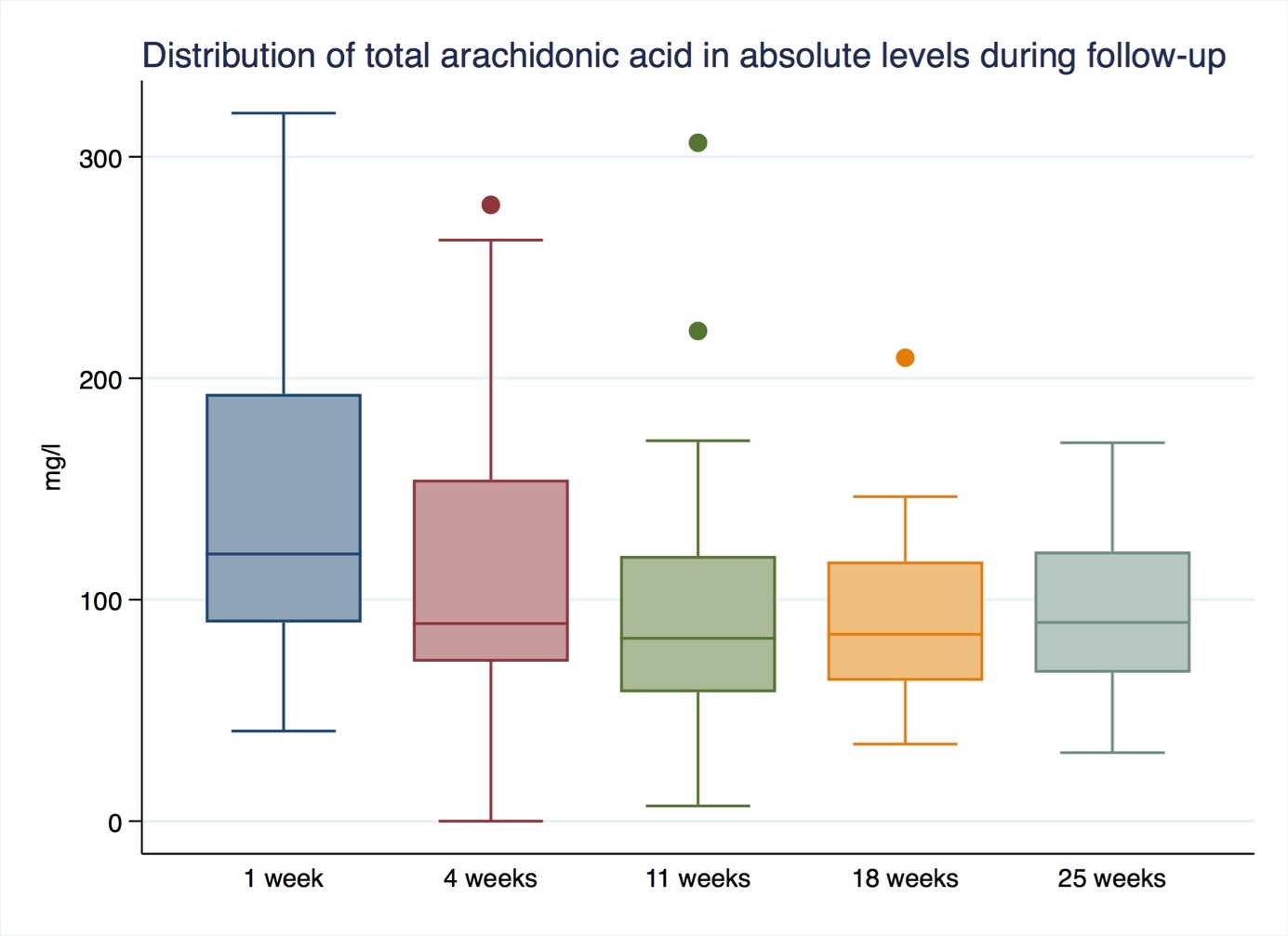

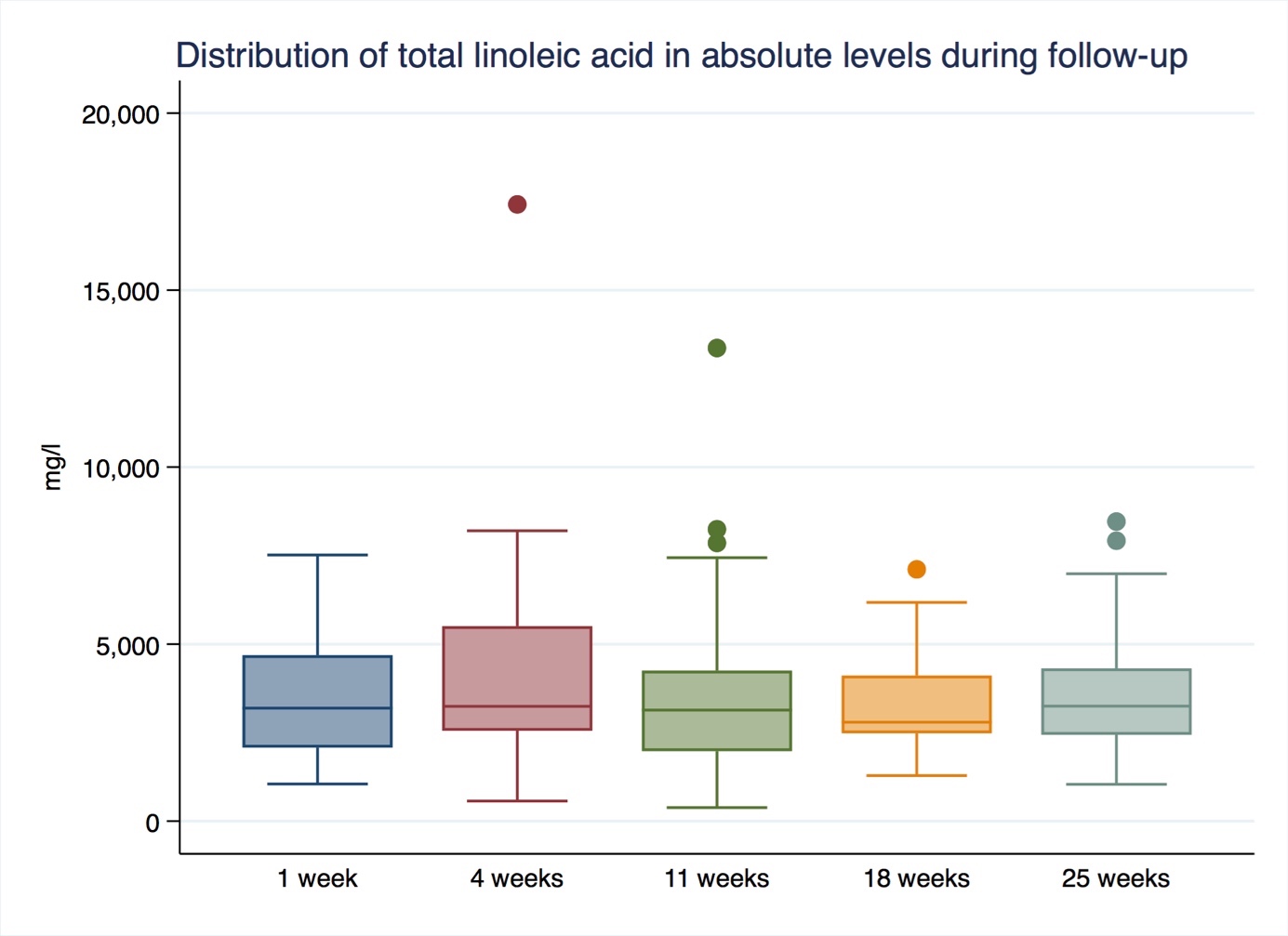


**S3. Box plots of the distribution of total amino acids**


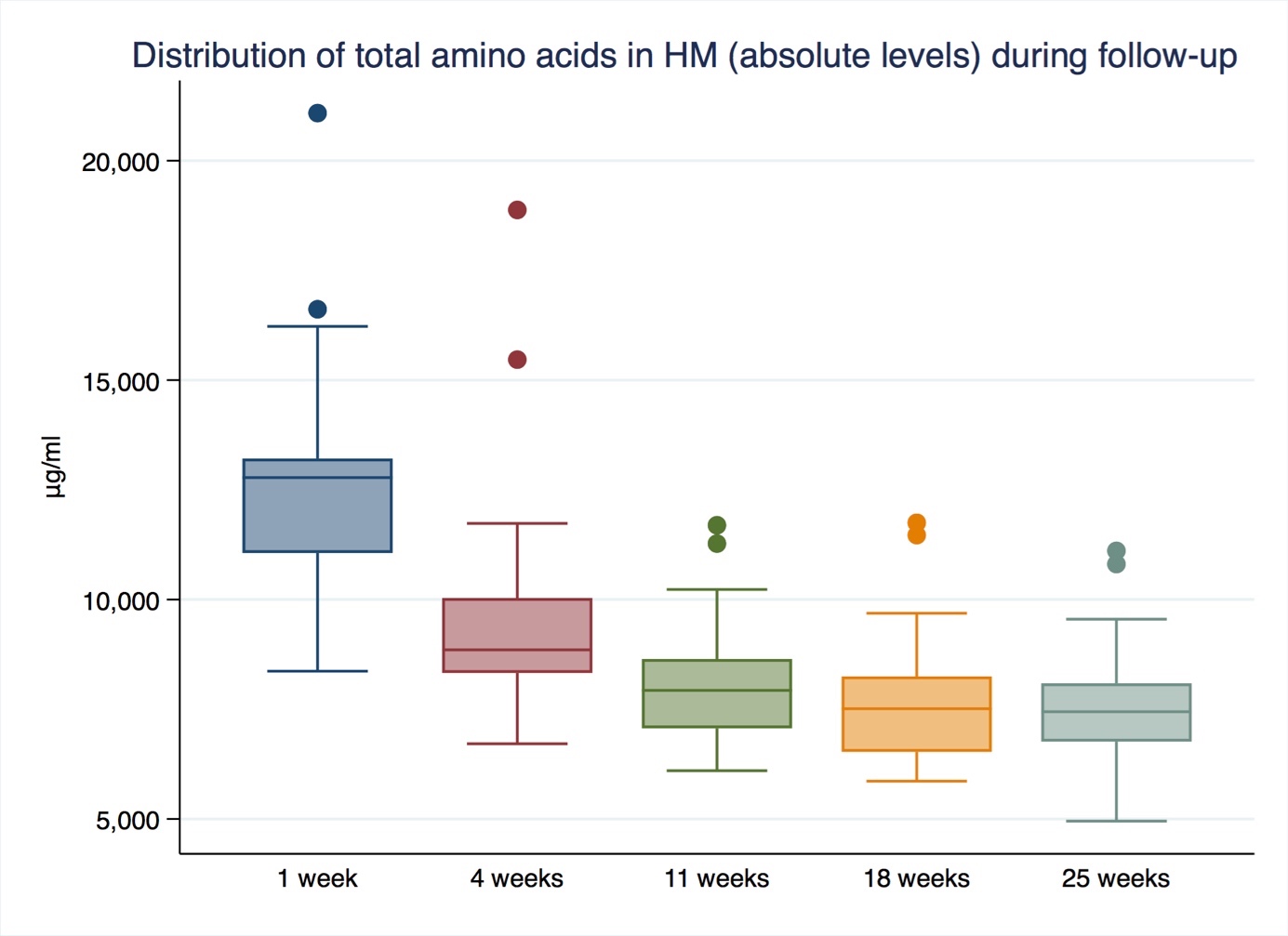


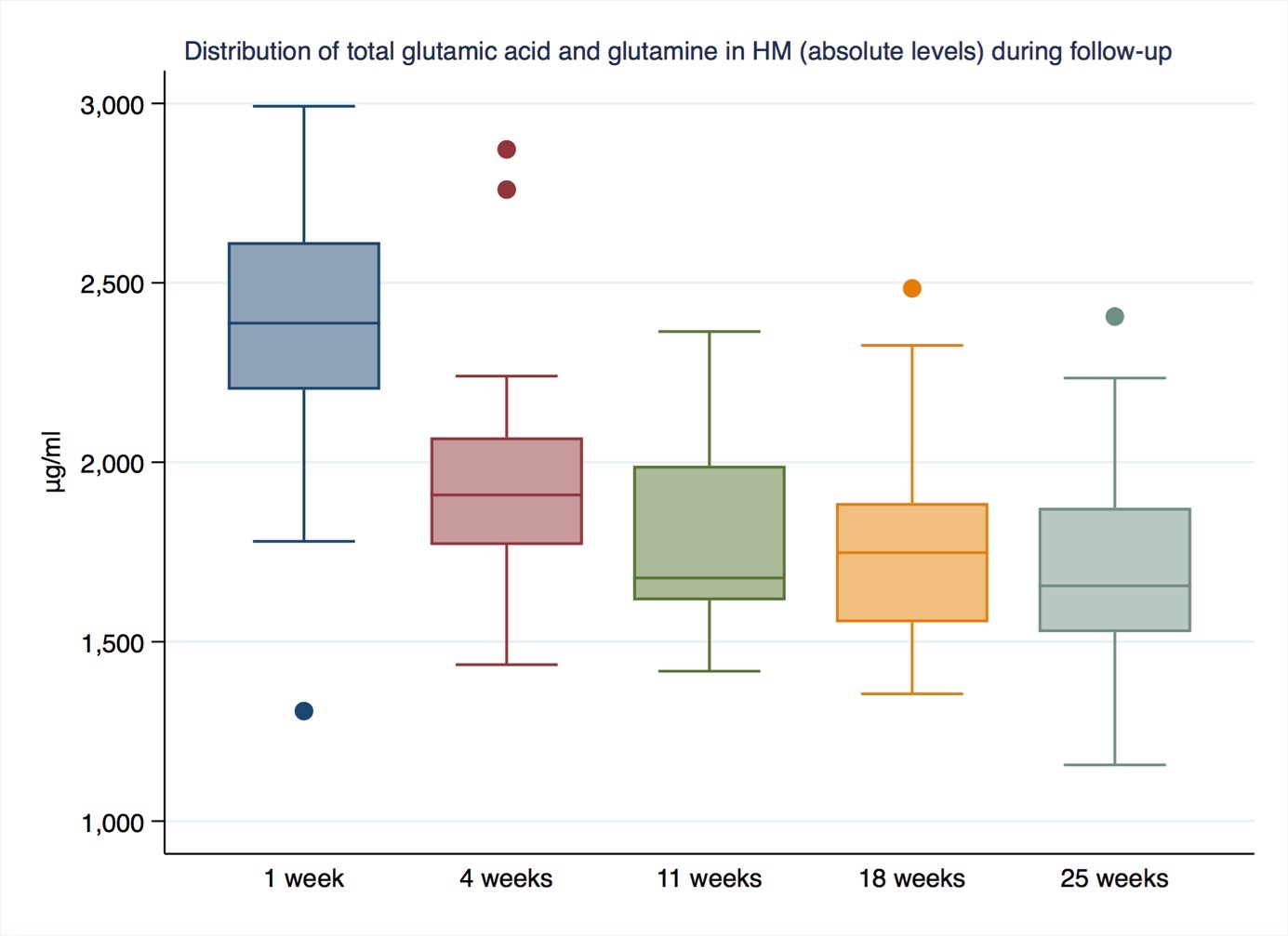


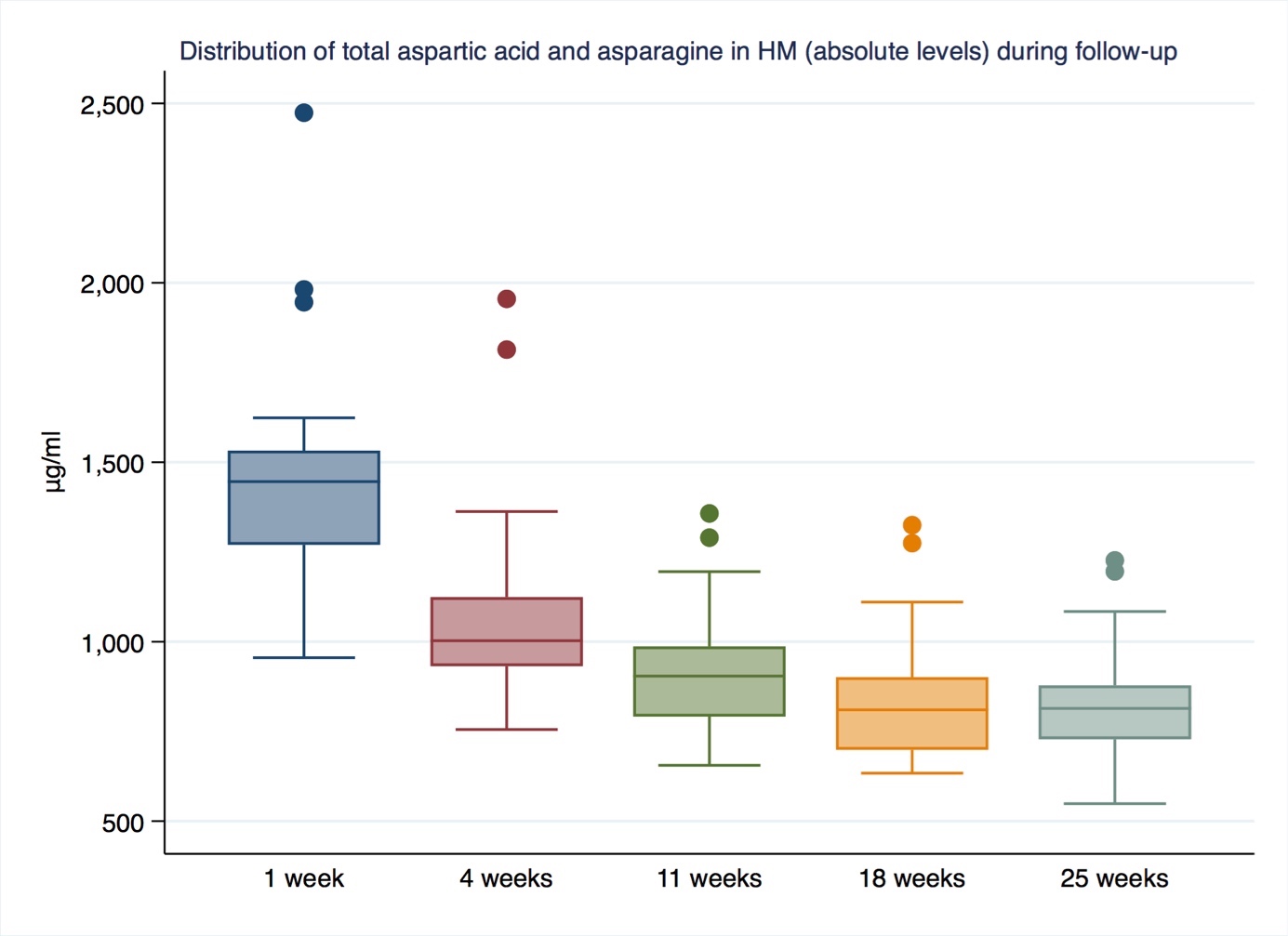


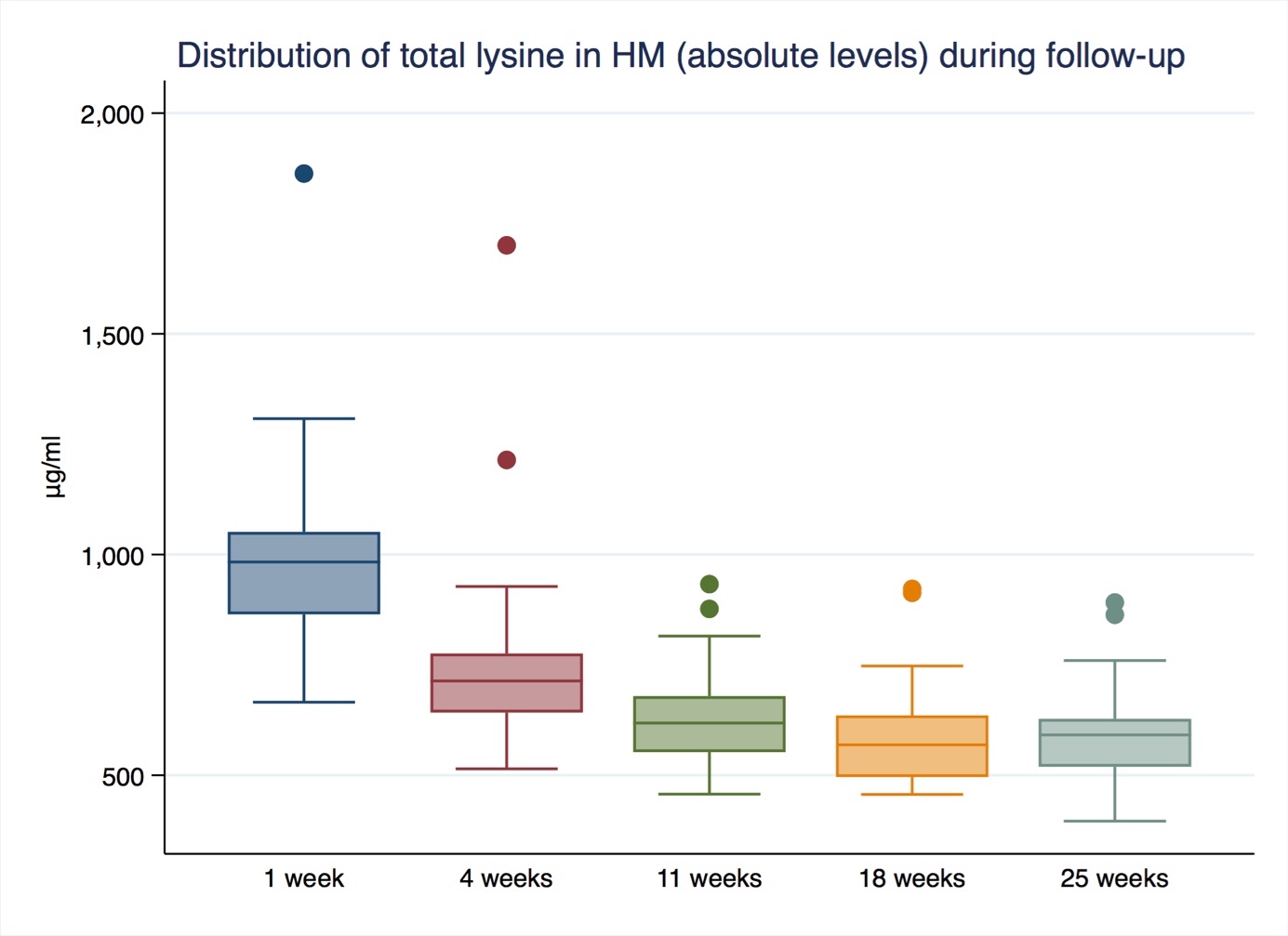


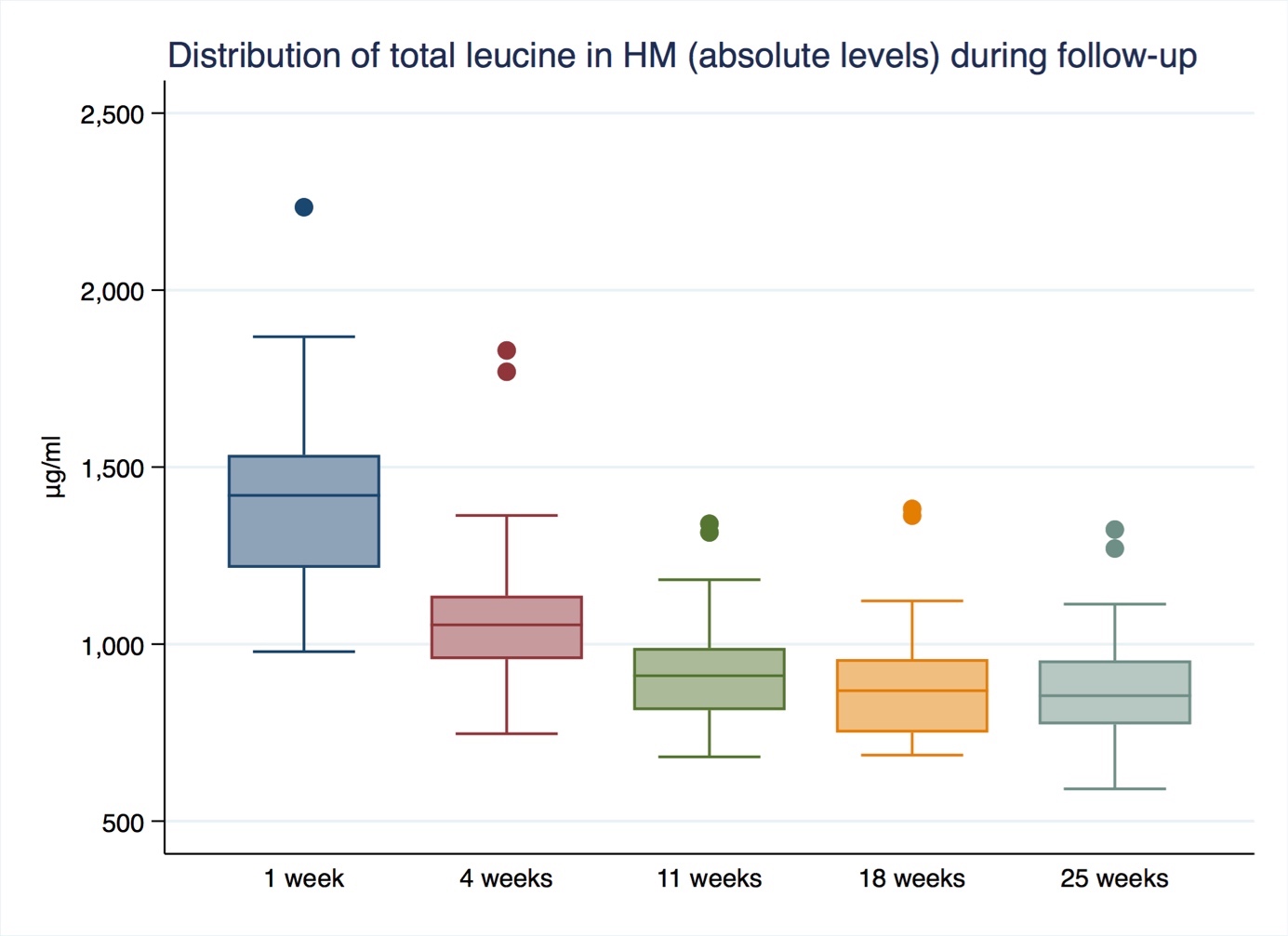


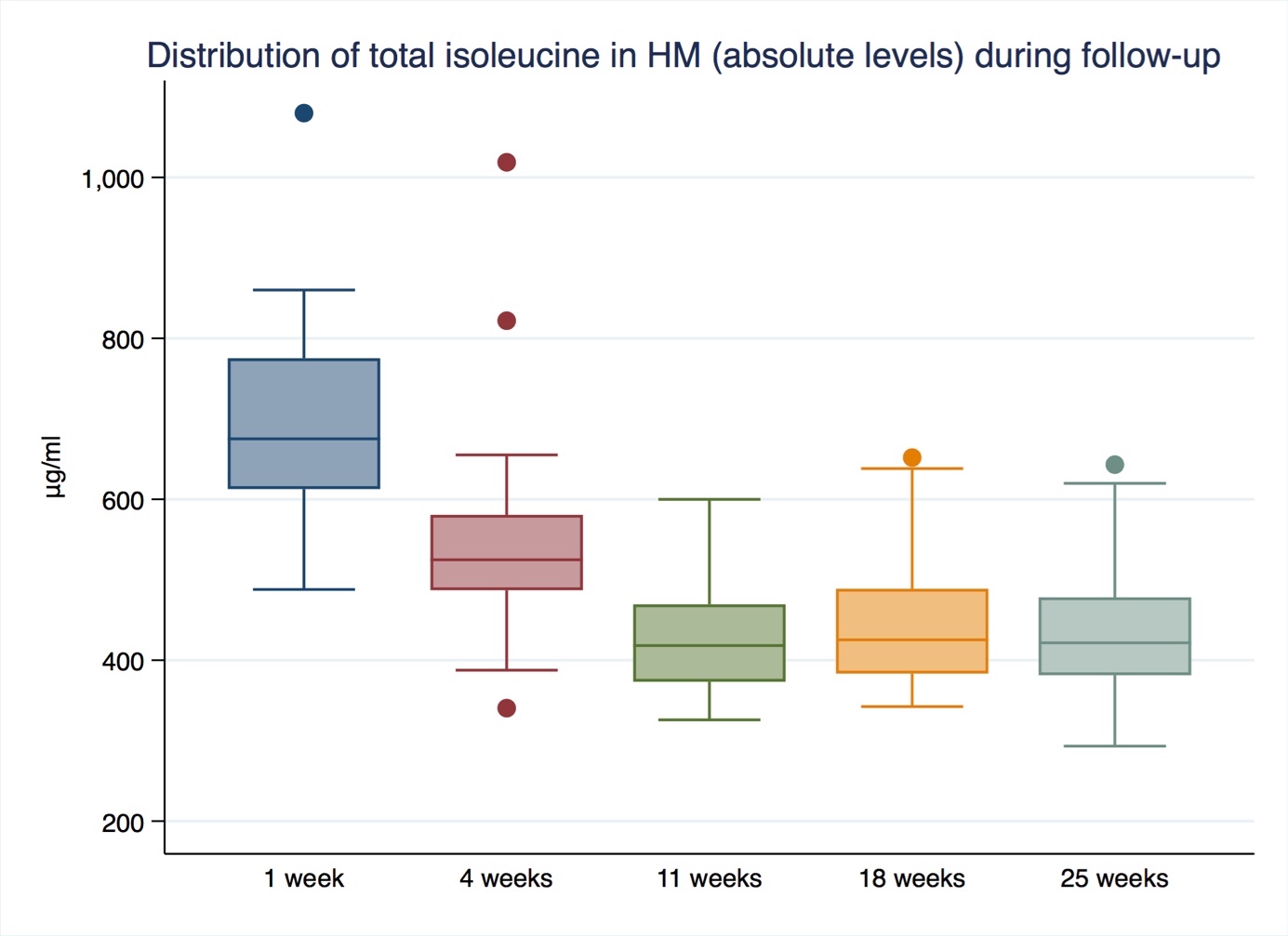


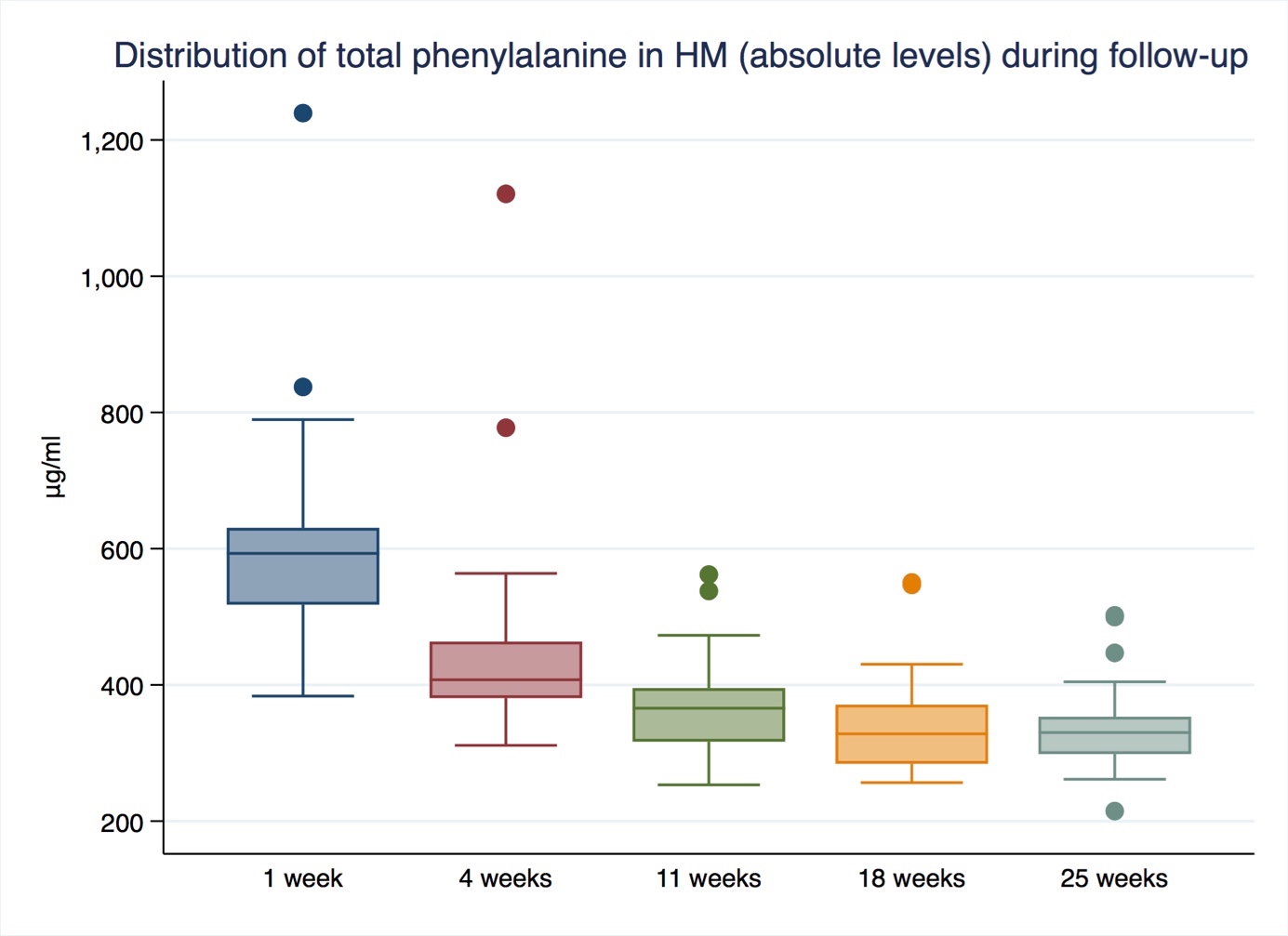


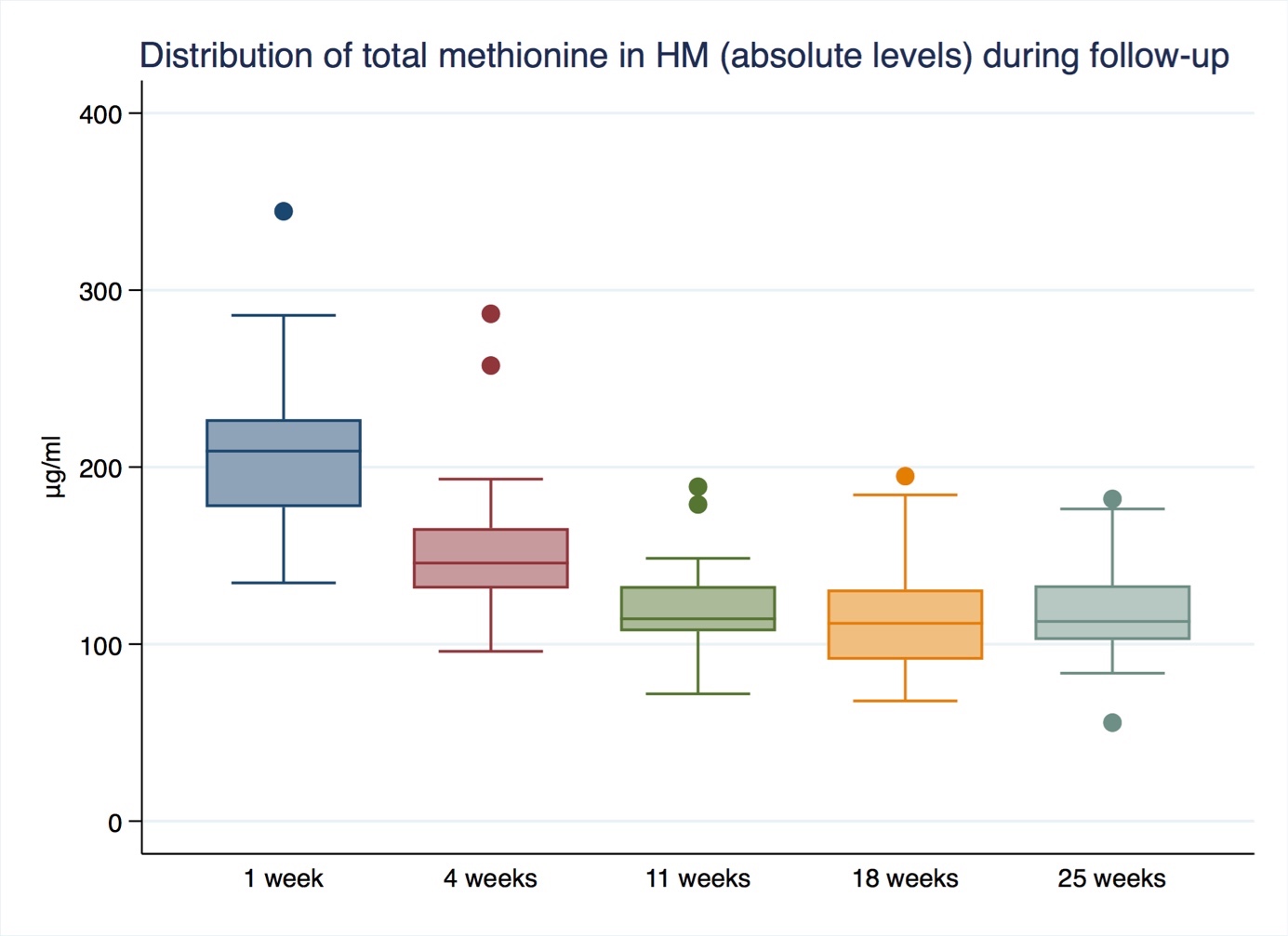


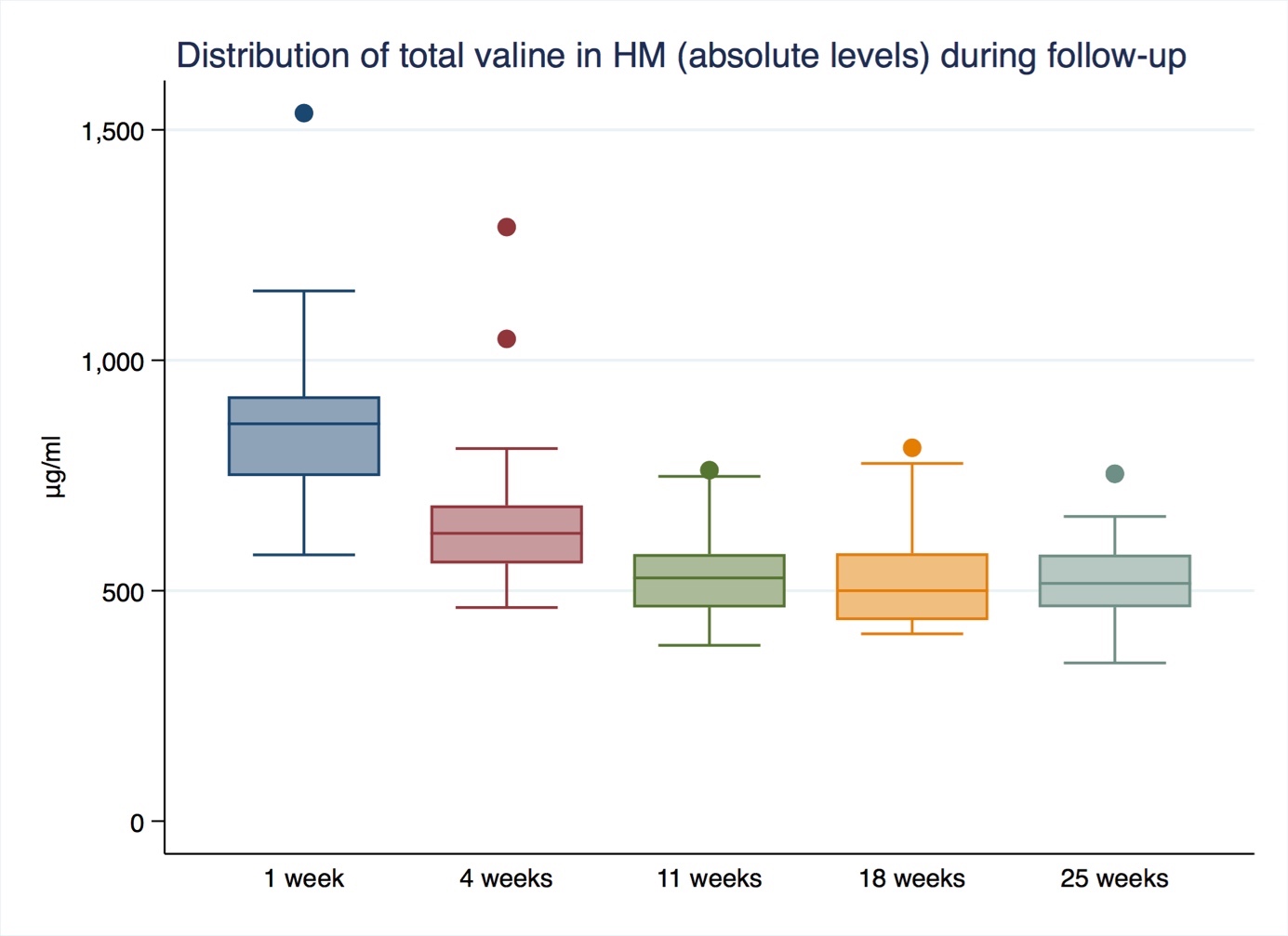


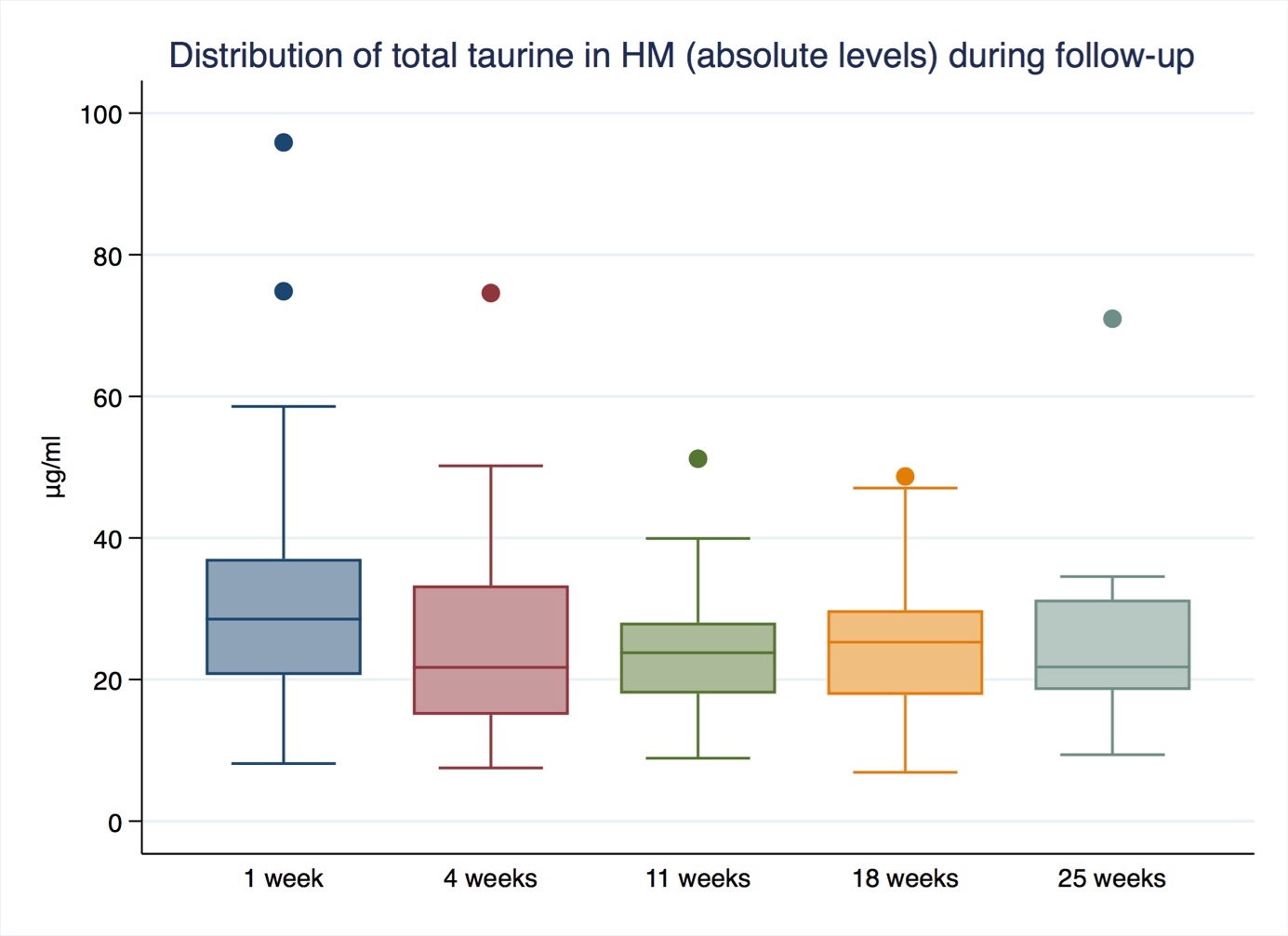


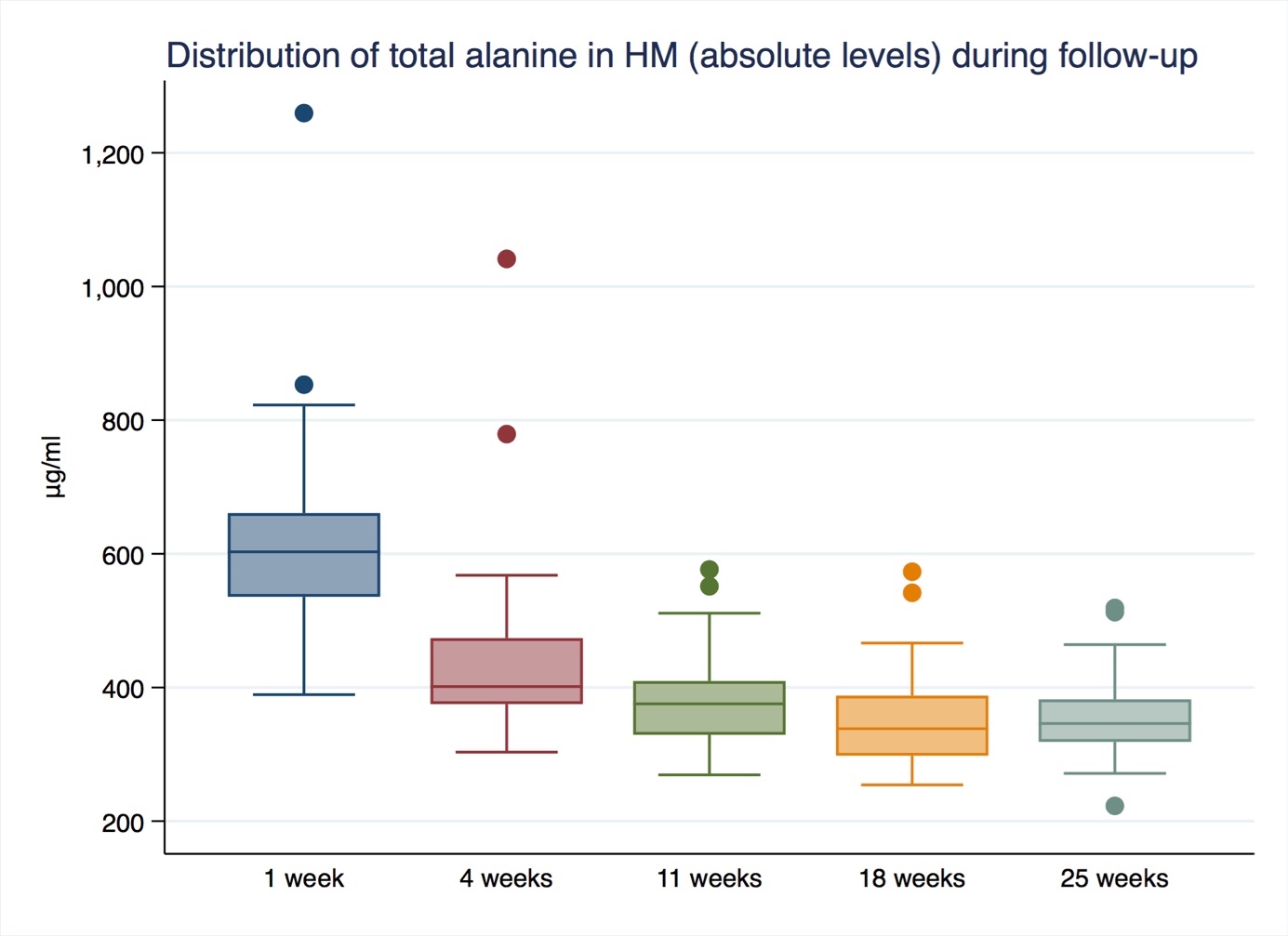


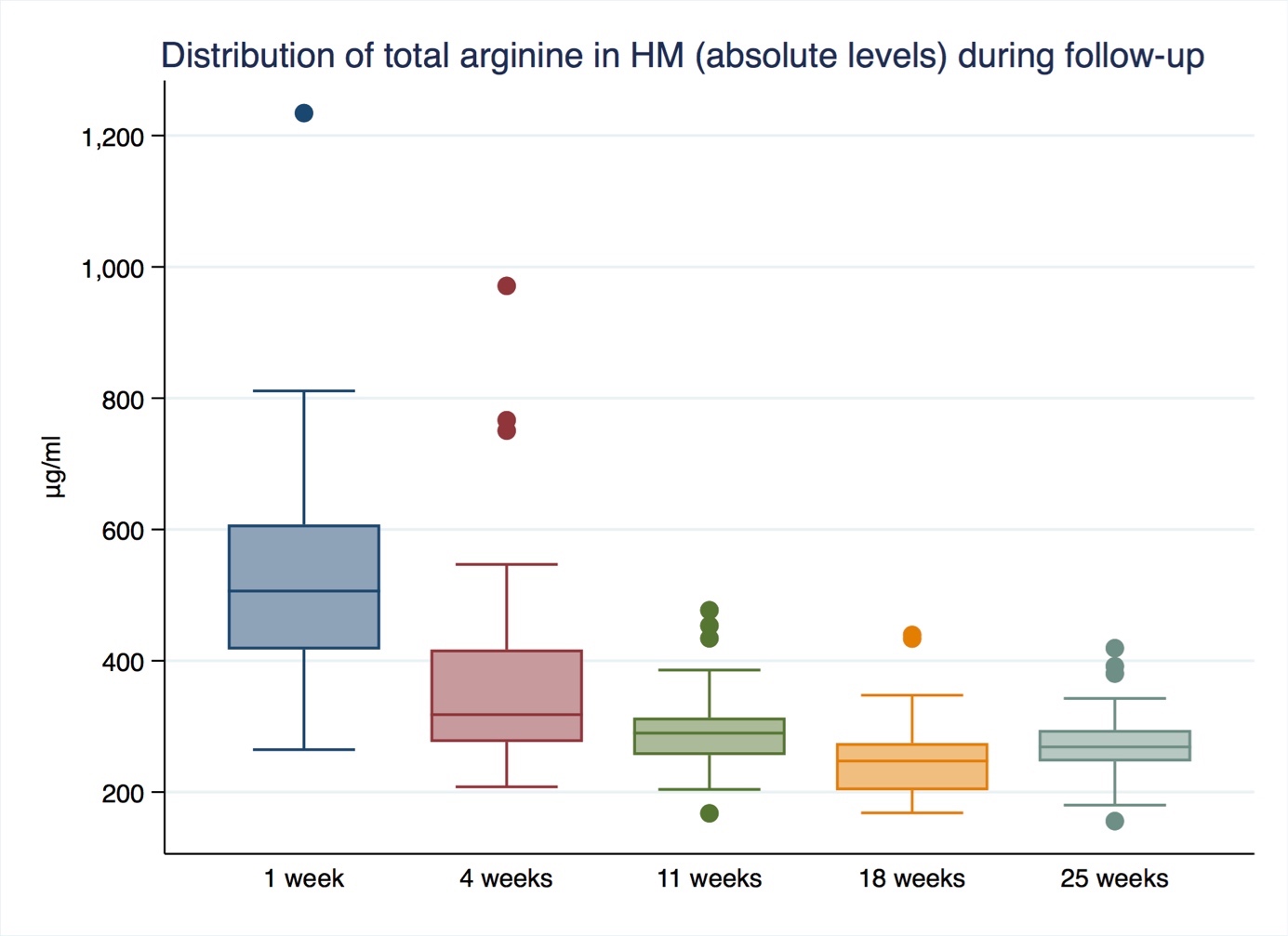


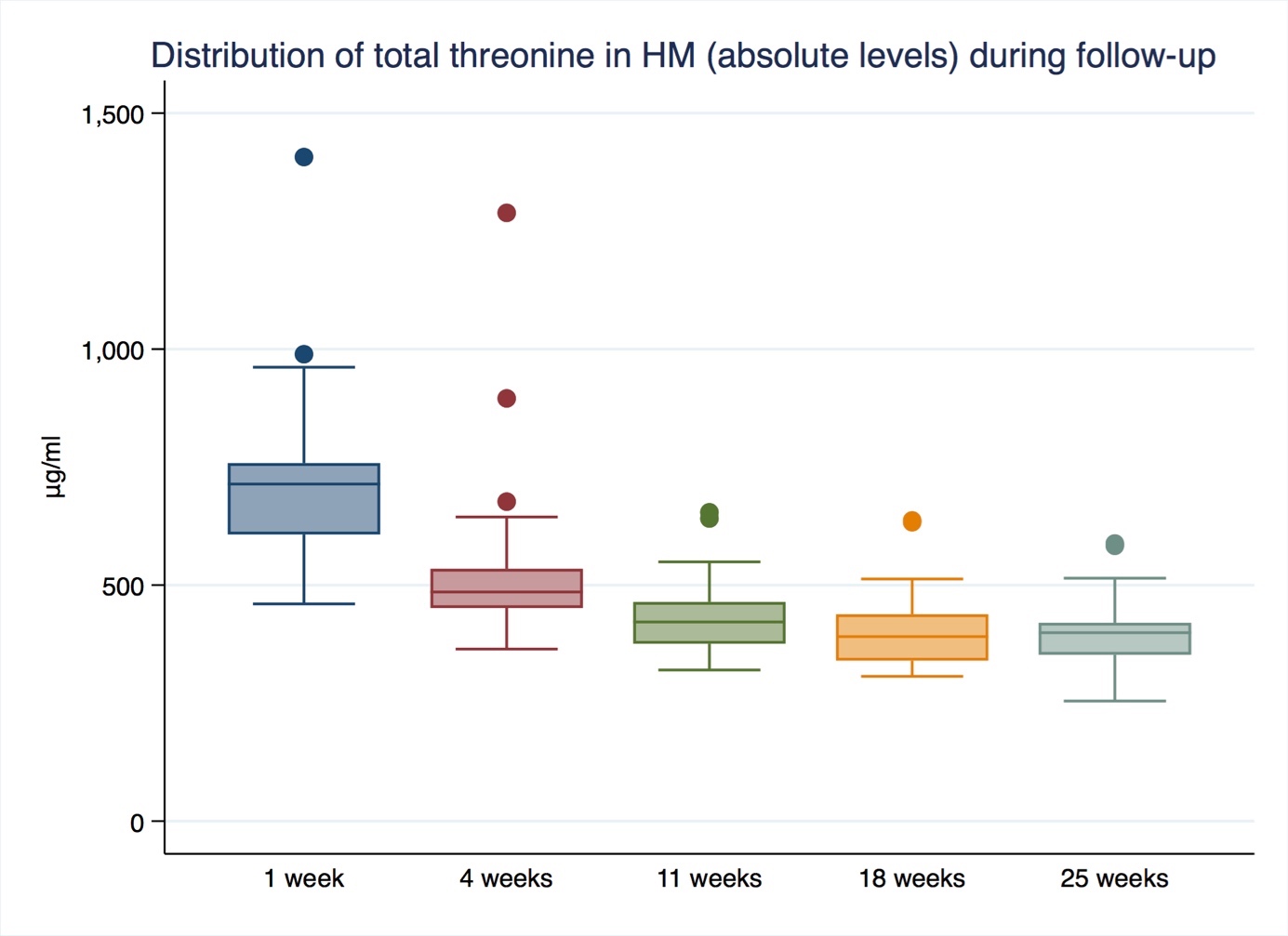


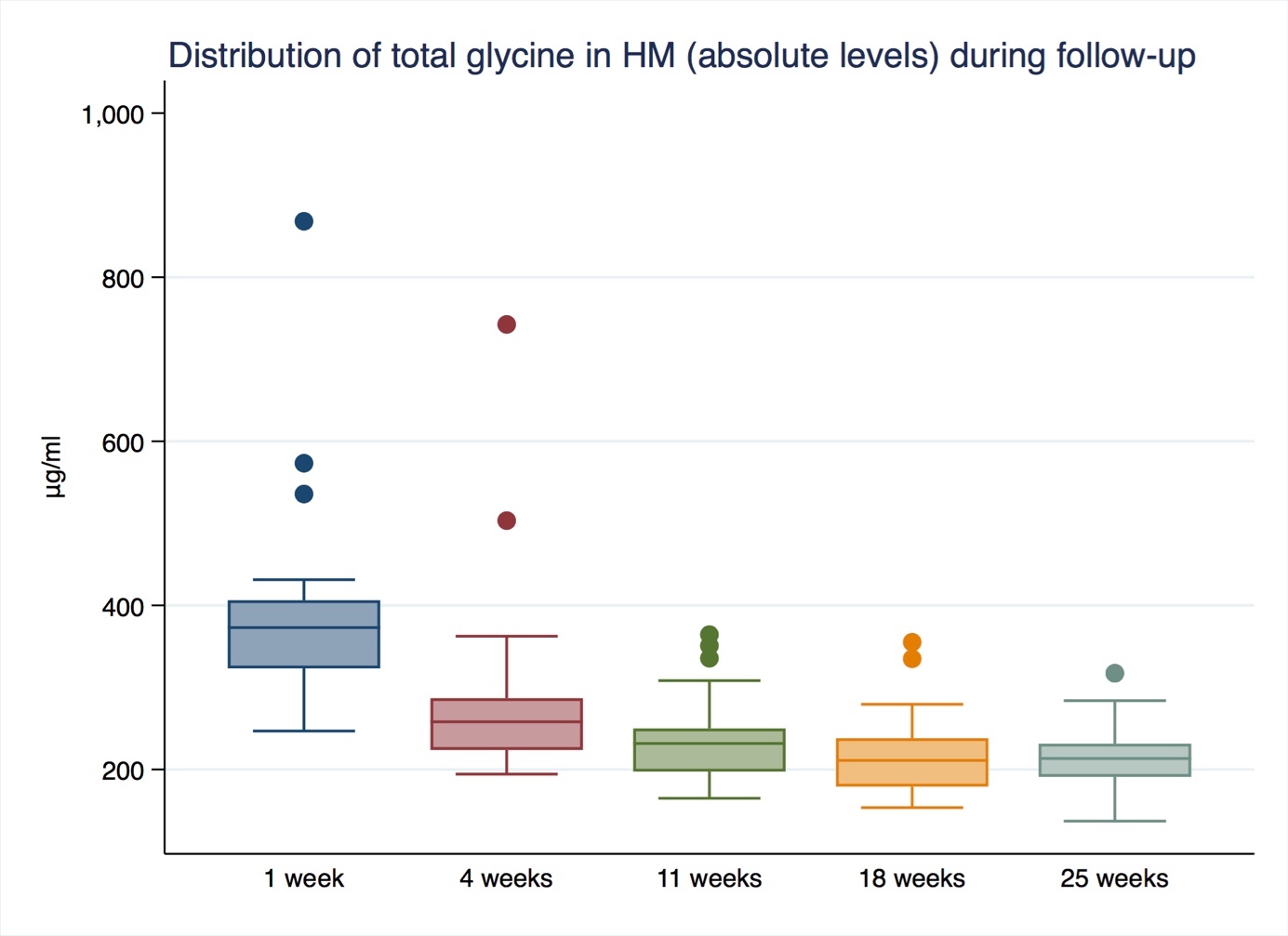


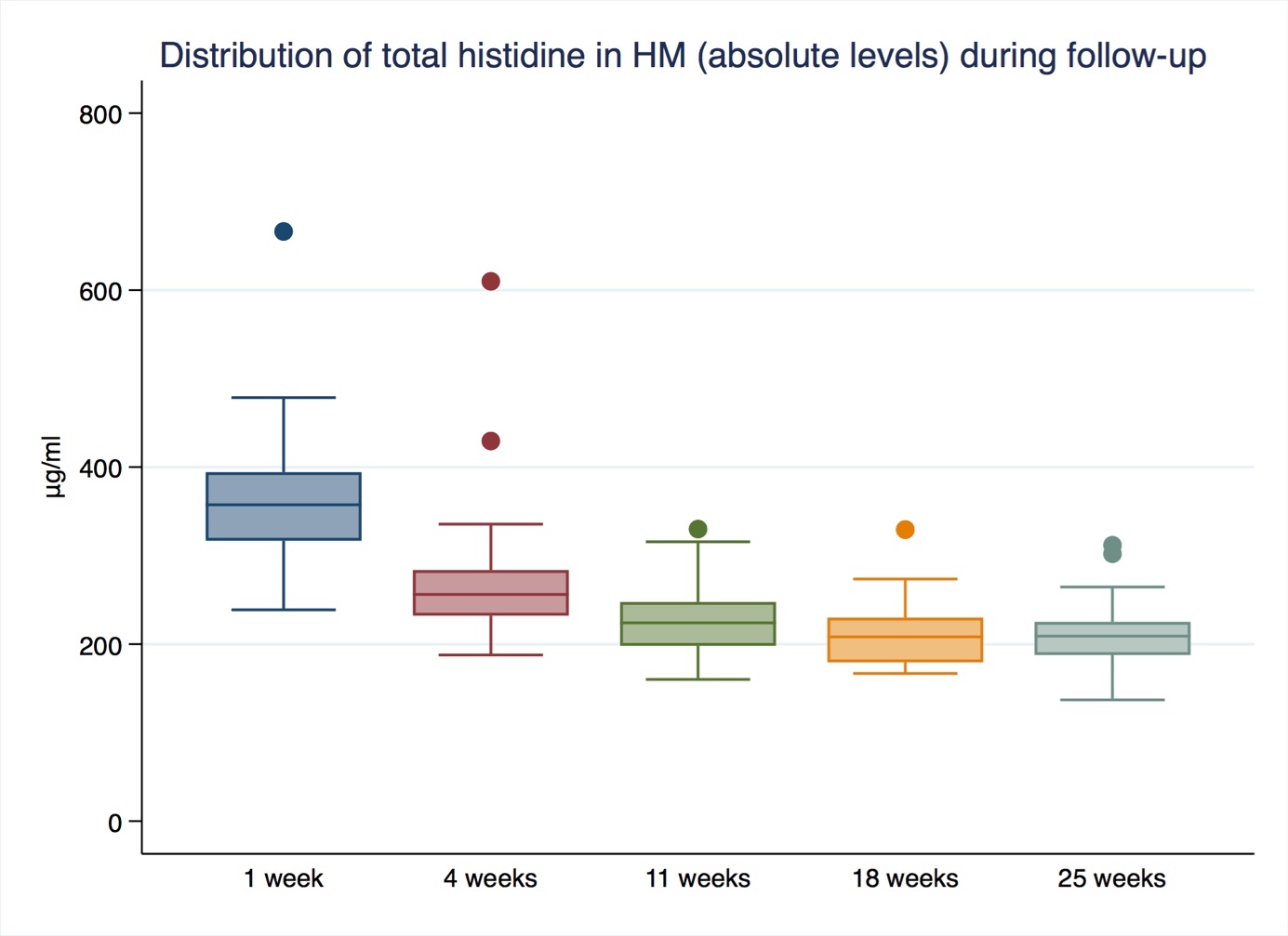


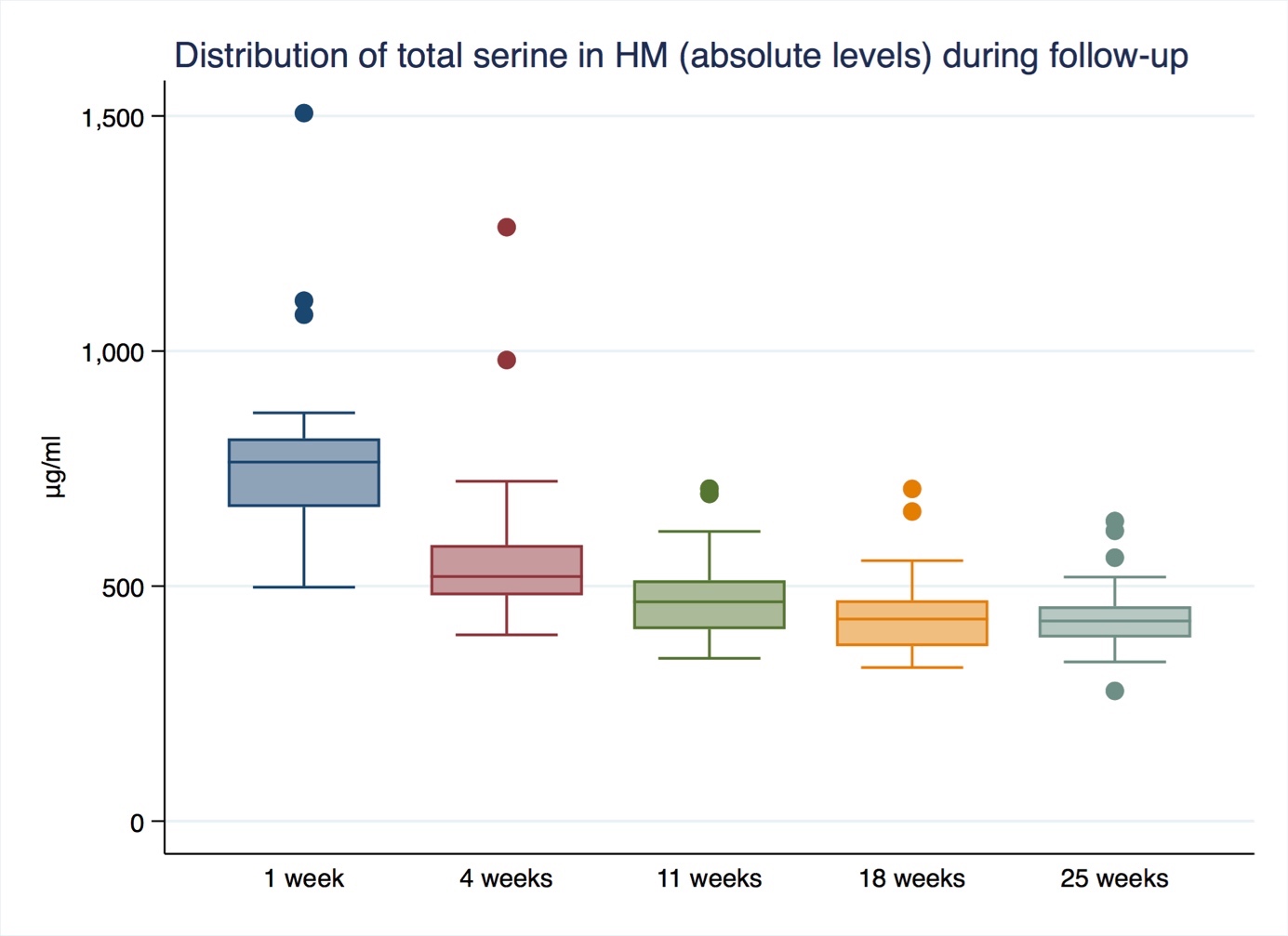


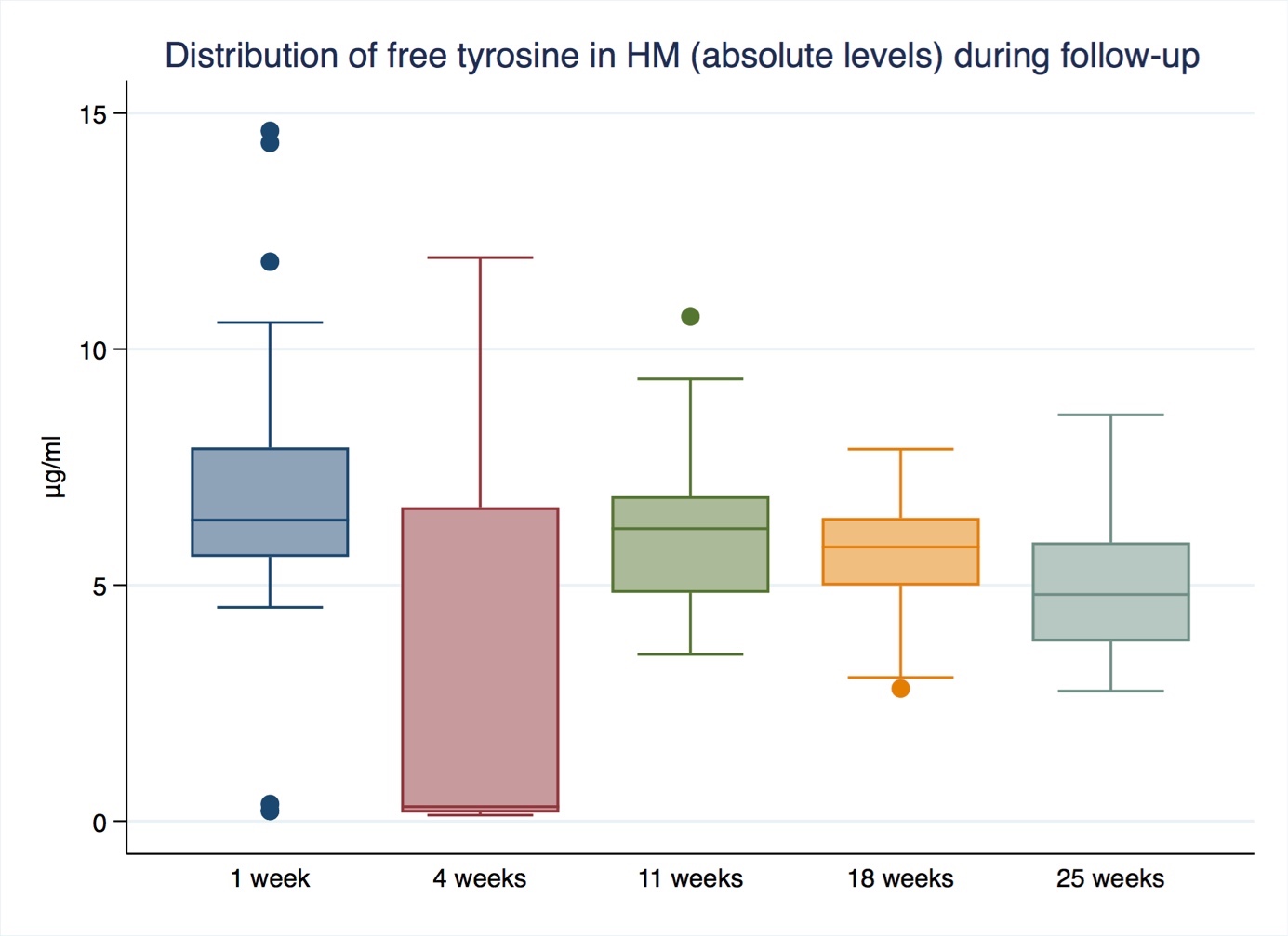


**S4. Evolution of relative levels of amino acids during follow-up**

|  | **Total** | **1 week** | **4 weeks** | **11 weeks** | **18 weeks** | **25 weeks** | ***P* value *** |
| --- | --- | --- | --- | --- | --- | --- | --- |
| **Percentage of total amino acids** |  |  |  |  |  |  |  |
| **Essential amino acids** |  |  |  |  |  |  |  |
| Total histidine/ total amino acids (%) | 2.81 (2.76; 2.87) | 2.89 (2.80; 2.95) | 2.87 (2.82; 2.90) | 2.80 (2.77; 2.83) | 2.76 (2.79; 2.84) | 2.77 (2.75; 2.81) | **<0.001** |
| Total isoleucine/ total amino acids (%) | 5.64 (5.42; 5.85) | 5.50 (5.34; 5.85) | 5.78 (5.52; 5.97) | 5.42 (5.15; 5.62) | 5.71 (5.59; 5.92) | 5.73 (5.62; 5.85) | **<0.001** |
| Total leucine/ total amino acids (%) | 11.54 (11.26; 11.67) | 11.25 (11.14; 11.55) | 11.59 (11.30; 11.74) | 11.50 (11.33; 11.60) | 11.62 (11.51; 11.78) | 11.64 (11.39; 11.79) | **0.006** |
| Total methionine/ total amino acids (%) | 1.59 (1.47; 1.66) | 1.67 (1.61; 1.71) | 1.62 (1.57; 1.66) | 1.50 (1.43; 1.59) | 1.47 (1.38; 1.59) | 1.57 (1.48; 1.64) | **<0.001** |
| Total phenylalanine/ total amino acids (%) | 4.53 (4.42; 4.67) | 4.61 (4.54; 4.79) | 4.66 (4.49; 4.76) | 4.50 (4.44; 4.62) | 4.44 (4.36; 4.59) | 4.44 (4.30; 4.51) | **<0.001** |
| Total threonine/ total amino acids (%) | 5.37 (5.22; 5.51) | 5.54 (5.44; 5.74) | 5.48 (5.38; 5.56) | 5.29 (5.16; 5.42) | 5.29 (5.16; 5.40) | 5.24 (5.14; 5.31) | **<0.001** |
| Total valine/ total amino acids (%) | 6.79 (6.63; 6.93) | 6.81 (6.75; 6.94) | 6.83 (6.70; 6.95) | 6.51 (6.43; 6.72) | 6.89 (6.67; 7.01) | 6.87 (6.76; 6.95) | **<0.001** |
| **Non-essential amino acids** |  |  |  |  |  |  |  |
| Total alanine/ total amino acids (%) | 4.68 (4.52; 4.87) | 4.86 (4.56; 5.13) | 4.64 (4.50; 4.88) | 4.73 (4.50; 4.82) | 4.65 (4.43; 4.80) | 4.66 (4.55; 4.79) | **0.04** |
| Total arginine/ total amino acids (%) | 3.57 (3.27; 3.98) | 4.02 (3.49; 4.68) | 3.55 (3.23; 4.31) | 3.58 (3.34; 3.77) | 3.31 (3.02; 3.51) | 3.52 (3.28; 3.76) | **0.001** |
| Total aspartic acid + asparagine/ total amino acids (%) | 11.16 (10.91; 11.44) | 11.46 (11.23; 11.73) | 11.25 (11.07; 11.50) | 11.28 (10.98; 11.41) | 10.93 (10.65; 11.07) | 10.86 (10.72; 11.08) | **<0.001** |
| Total glutamic acid + glutamine/ total amino acids (%) | 21.66 (20.28; 22.78) | 19.39 (18.55; 20.47) | 20.85 (20.16; 21.69) | 22.50 (21.66; 23.23) | 22.86 (21.88; 24.00) | 22.35 (21.72; 23.37) | **<0.001** |
| Total glycine/ total amino acids (%) | 2.89 (2.73; 3.03) | 2.95 (2.75; 3.14) | 2.89 (2.74; 3.07) | 2.83 (2.75; 3.01) | 2.86 (2.69; 2.93) | 2.82 (2.69; 2.97) | 0.09 |
| Total serine/ total amino acids (%) | 5.81 (5.66; 6.02) | 6.06 (5.83; 6.28) | 5.87 (5.68; 6.12) | 5.83 (5.71; 6.02) | 5.75 (5.63; 5.81) | 5.69 (5.60; 5.77) | **<0.001** |
| Total taurine/ total amino acids (%) | 0.27 (0.19; 0.36) | 0.21 (0.17; 0.34) | 0.22 (0.17; 0.37) | 0.28 (0.20; 0.35) | 0.32 (0.25; 0.39) | 0.29 (0.22; 0.39) | **<0.001** |
| Total tyrosine/ total amino acids (%) | 3.62 (3.49; 3.79) | 3.77 (3.68; 3.92) | 3.78 (3.62; 3.88) | 3.6 1(3.48; 3.76) | 3.49 (3.42; 3.60) | 3.51 (3.37; 3.59) | **<0.001** |
| Total lysine/ total amino acids (%) | 7.77 (7.65; 7.89) | 7.78 (7.64; 7.79) | 7.79 (7.67; 7.98) | 7.80 (7.69; 7.88) | 7.66 (7.55; 7.76) | 7.83 (7.71; 7.89) | **0.005** |
| **Percentage of free amino acids** |  |  |  |  |  |  |  |
| **Essential amino acids** |  |  |  |  |  |  |  |
| Free histidine/ sum of free amino acids (%) | 1.01 (0.78; 1.31) | 1.57 (1.22; 1.83) | 1.15 (0.94; 1.37) | 1.01 (0.84; 1.12) | 0.79 (0.69; 0.88) | 0.80 (0.66; 0.89) | **<0.001** |
| Free isoleucine/ sum of free amino acids (%) | 0.53 (0.38; 0.70) | 0.54 (0.32; 0.73) | 0.55 (0.42; 0.69) | 0.62 (0.46; 0.76) | 0.44 (0.34; 0.56) | 0.45 (0.36; 0.66) | **0.03** |
| Free leucine/ sum of free amino acids (%) | 0.90 (0.69; 1.22) | 1.21 (0.83; 1.79) | 1.05 (0.81; 1.58) | 0.90 (0.75; 1.20) | 0.86 (0.59; 0.99) | 0.70 (0.57; 0.91) | **<0.001** |
| Free lysine/ sum of free amino acids (%) | 0.63 (0.49; 0.97) | 0.80 (0.56; 1.44) | 0.70 (0.56; 1.18) | 0.66 (0.55; 0.97) | 0.56 (0.38; 0.65) | 0.51 (0.38; 0.79) | **<0.001** |
| Free methionine/ sum of free amino acids (%) | 0.35 (0.28; 0.42) | 0.35 (0.26; 0.47) | 0.34 (0.29; 0.43) | 0.34 (0.25; 0.40) | 0.36 (0.29; 0.40) | 0.36 (0.32; 0.42) | 0.59 |
| Free phenylalanine/ sum of free amino acids (%) | 0.66 (0.58; 0.85) | 0.73 (0.53; 0.89) | 0.72 (0.62; 0.85) | 0.69 (0.58; 0.87) | 0.60 (0.55; 0.69) | 0.65 (0.58; 0.80) | 0.16 |
| Free threonine/ sum of free amino acids (%) | 2.32 (1.72; 2.99) | 2.36 (2.01; 3.40) | 2.50 (1.71; 3.37) | 2.24 (1.66; 2.73) | 2.11 (1.58; 2.75) | 2.39 (1.92; 2.89) | 0.31 |
| Free tryptophan/ sum of free amino acids (%) | 0.17 (0.13; 0.23) | 0.18 (0.13; 0.34) | 0.13 (0.09; 0.25) | 0.15 (0.14; 0.19) | 0.17 (0.15; 0.19) | 0.17 (0.15; 0.21) | 0.35 |
| Free valine/ sum of free amino acids (%) | 4.48 (3.45; 5.87) | 6.05 (4.85; 6.95) | 5.61 (4.55; 7.19) | 4.27 (3.52; 5.13) | 3.70 (2.87; 4.35) | 3.44 (3.01; 4.36) | **<0.001** |
| **Non-essential amino acids** |  |  |  |  |  |  |  |
| Free alanine/ sum of free amino acids (%) | 5.87 (5.22; 6.86) | 5.68 (6.01; 7.30) | 6.00 (5.65; 6.86) | 5.68 (5.14; 6.21) | 5.64 (4.86; 7.07) | 5.95 (5.43; 6.60) | 0.14 |
| Free arginine/ sum of free amino acids (%) | 0.79 (0.58; 1.13) | 0.96 (0.82; 1.73) | 0.97 (0.78; 1.17) | 0.77 (0.58; 0.97) | 0.62 (0.44; 0.77) | 0.68 (0.47; 0.85) | **<0.001** |
| Free asparagine/ sum of free amino acids (%) | 0.75 (0.57; 0.99) | 0.60 (0.44; 0.74) | 0.83 (0.64; 1.21) | 0.84 (0.71; 0.99) | 0.73 (0.60; 0.93) | 0.73 (0.56; 0.94) | **0.005** |
| Free aspartic acid/ sum of free amino acids (%) | 2.05 (1.55; 2.69) | 2.25 (2.00; 2.77) | 1.52 (1.24; 1.94) | 1.99 (1.71; 2.69) | 2.05 (1.56; 2.67) | 2.41 (1.81; 3.36) | **0.002** |
| Free glutamic acid/ sum of free amino acids (%) | 50.17 (47.03; 53.41) | 51.00 (46.51; 55.19) | 49.12 (47.44; 51.62) | 51.52 (47.03; 55.56) | 49.70 (45.65; 53.88) | 51.23 (47.58; 52.77) | 0.91 |
| Free glutamine/ sum of free amino acids (%) | 12.88 (7.71; 17.22) | 6.68 (4.04; 10.34) | 11.74 (5.26; 14.39) | 14.44 (11.40; 17.62) | 17.14 (13.12; 20.07) | 15.83 (12.23; 19.09) | **<0.001** |
| Free glycine/ sum of free amino acids (%) | 2.62 (2.18; 3.11) | 2.61 (2.11; 3.11) | 2.84 (2.62; 3.54) | 2.51 (1.99; 3.01) | 2.39 (2.12; 3.05) | 2.68 (2.33; 3.07) | **0.002** |
| Free serine/ sum of free amino acids (%) | 3.51 (2.97; 4.20) | 2.76 (2.51; 3.10) | 3.57 (3.10; 4.42) | 3.62 (3.22; 4.09) | 3.63 (3.24; 4.42) | 3.61 (3.32; 4.29) | **<0.001** |
| Free taurine/ sum of free amino acids (%) | 6.58 (4.70; 9.15) | 9.20 (7.56; 10.88) | 8.00 (5.17; 10.20) | 6.06 (4.47; 7.24) | 5.55 (4.12; 6.70) | 5.53 (3.74; 8.02) | **<0.001** |
| Free tyrosine/ sum of free amino acids (%) | 1.23 (0.93; 1.69) | 1.75 (1.50; 2.20) | 0.09 (0.06; 1.57) | 1.34 (1.10; 1.64) | 1.15 (0.92; 1.31) | 1.05 (0.93; 1.24) | **<0.001** |
| * *P* value on the evolution of the different items during follow-up.  For continuous variables, median and inter-quartile range are provided. For binary and variables with categories, n and percentages are provided. | | | | | | | |

# S5. Multilevel models of total amino acids

| **Total amino acids** | Multivariate model serine (µg/ml) | *P* value | Multivariate model taurine (µg/ml) | *P* value |
| --- | --- | --- | --- | --- |
| **Maternal diet (food-frequency questionnaire)** |  |  |  |  |
| Grains, white roots and tubers |  |  |  |  |
| Pulses: beans, peas and lentils |  |  |  |  |
| Nuts and seeds |  |  |  |  |
| Milk and milk products |  |  |  |  |
| Meat, poultry, and fish | 442.67 (219.36; 665.98) | <0.001 |  |  |
| Eggs |  |  |  |  |
| Dark green leafy vegetables |  |  |  |  |
| Other vitamin A rich fruits and vegetables |  |  |  |  |
| Other vegetables |  |  |  |  |
| Other fruits |  |  |  |  |
| Insects, small rodents and other small animals |  |  |  |  |
| Red palm oil | 288.85 (139.71; 437.99) | <0.001 |  |  |
| Other oils and fats |  |  |  |  |
| Condiments and seasonings |  |  |  |  |
| Other beverages and foods |  |  |  |  |
| Sweet foods |  |  |  |  |
| Sweet beverages |  |  |  |  |
| **Food security during follow-up** |  |  |  |  |
| Household hunger scale index (HHS) |  |  |  |  |
| Little to no hunger in the household | reference |  | reference |  |
| Moderate hunger in the household | -110.85 (-187.56; -34.14) | 0.008 | -9.37 (-17.58; -1.16) | 0.03 |
| Severe hunger in the household | 35.41 (-213.19; 284.00) | 0.78 | -4.74 (-31.48; 22.00) | 0.73 |
| Undernourished mother at delivery |  |  |  |  |
| Female infant |  |  |  |  |
| **Follow-up visits** |  |  |  |  |
| 1st week | reference |  | reference |  |
| 4 weeks | -234.50 (-292.58; -176.42) | <0.001 | -4.76 (-10.64; 1.13) | 0.11 |
| 11 weeks | -329.40 (-381.93; -276.88) | <0.001 | -9.90 (-15.53; -4.28) | 0.001 |
| 18 weeks | -343.81 (-407.38; -280.24) | <0.001 | -10.17 (-16.98; -3.37) | 0.003 |
| 25 weeks | -372.98 (-440.18; -305.78) | <0.001 | -10.40 (-17.59; -3.21) | 0.005 |
| **Milk-type group** |  |  |  |  |
| Milk-type group I | -73.40 (-147.80; 1.00) | 0.05 | -10.33 (-18.37; -2.28) | 0.01 |
| Milk-type group II | -60.22 (-145.95; 25.51) | 0.17 | -7.15 (-16.28; 1.99) | 0.13 |
| Milk-type group III | reference |  | reference |  |
| Milk-type group IV | 36.41 (-67.19; 140.01) | 0.49 | -0.88 (-12.07; 10.32) | 0.88 |

**S6. Box plots of the distribution of free amino acids**


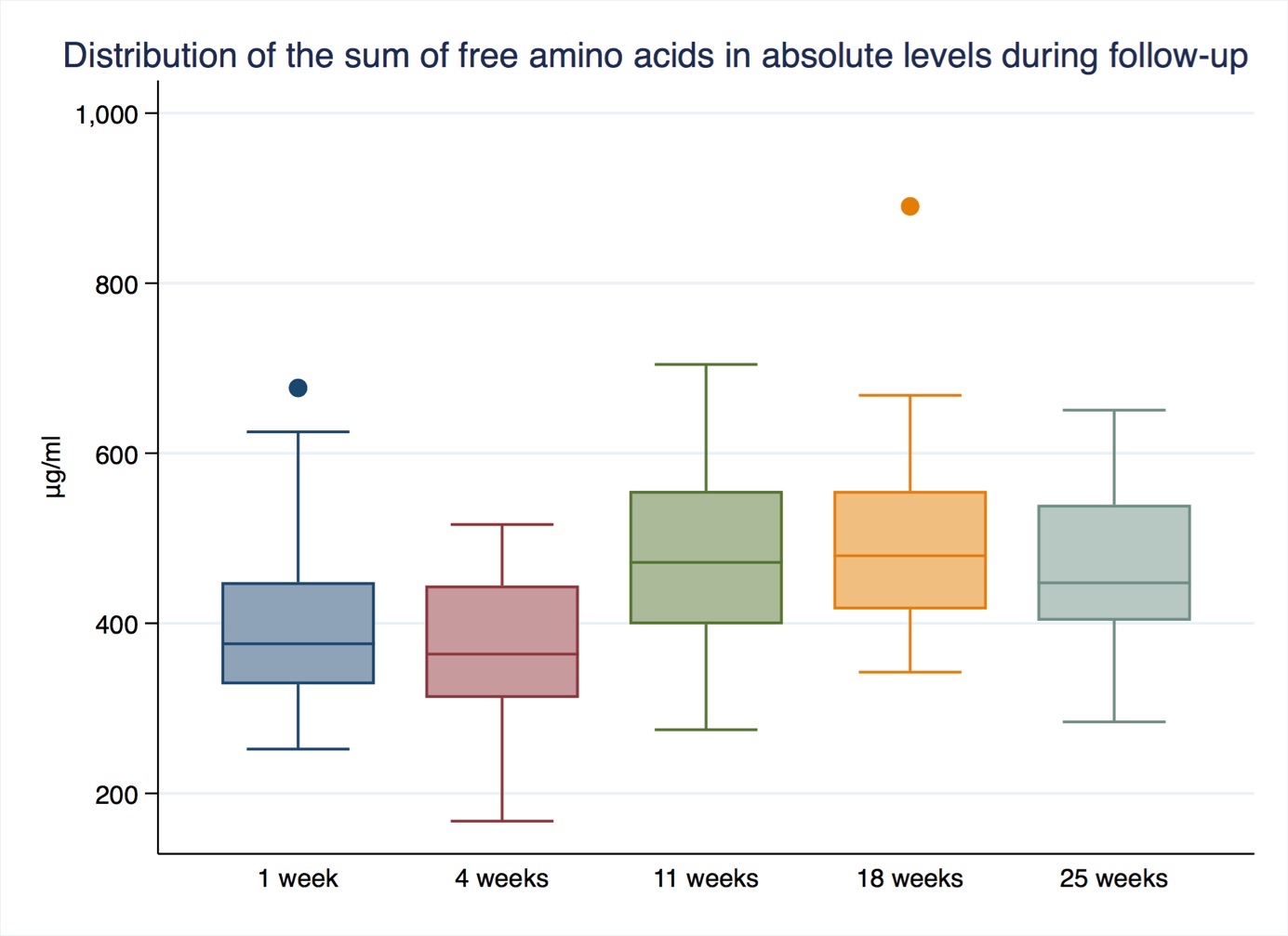


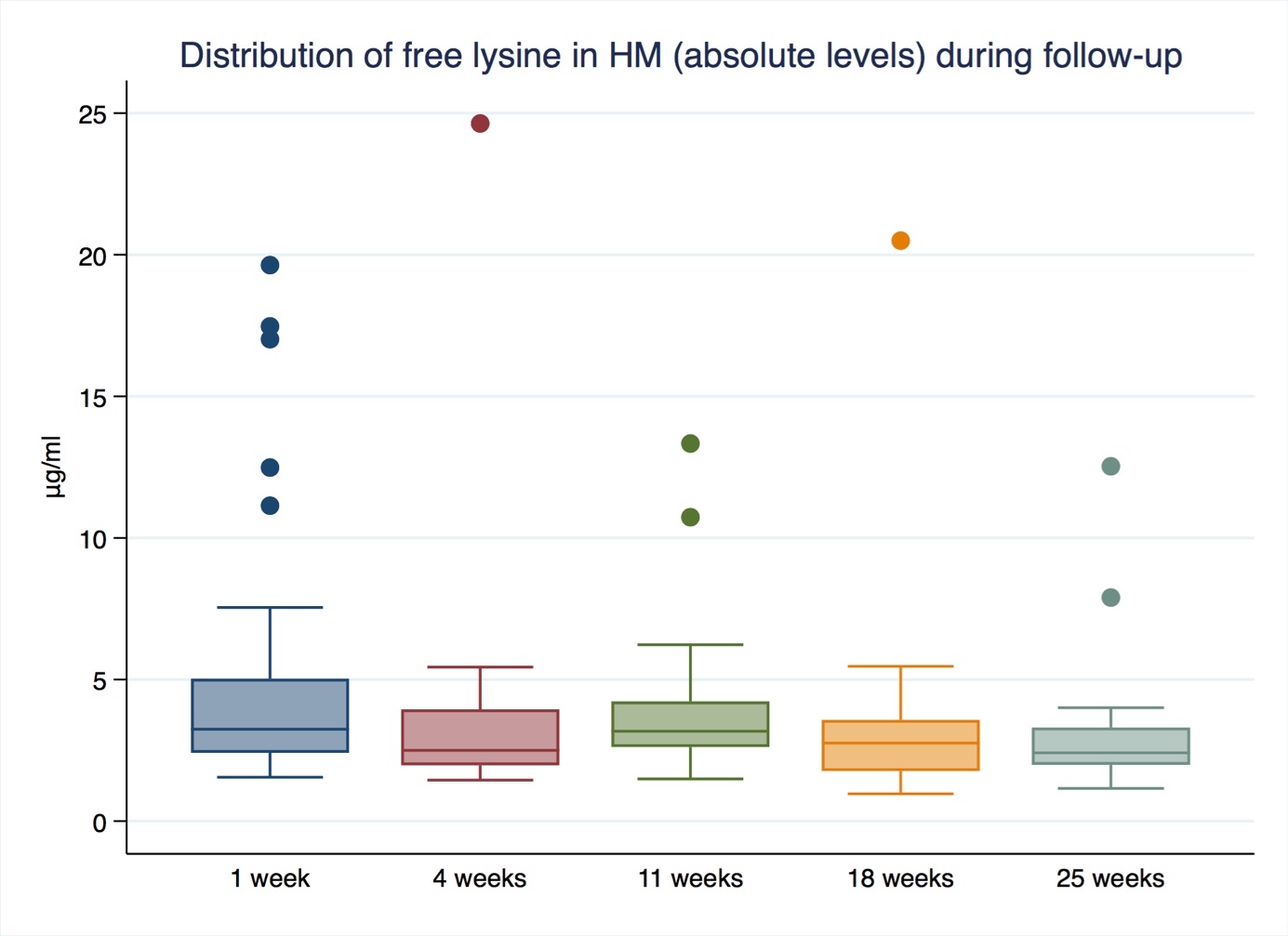


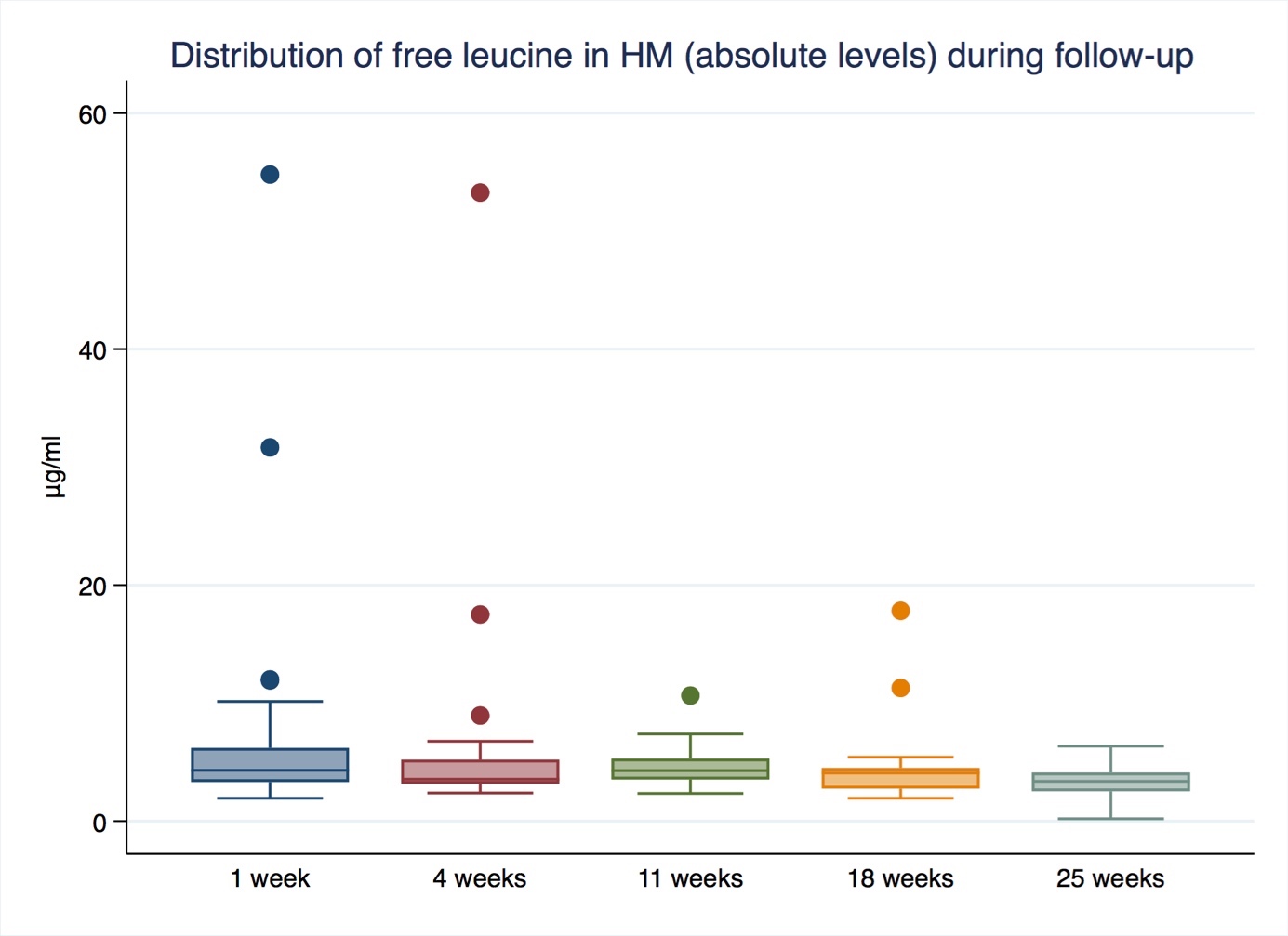

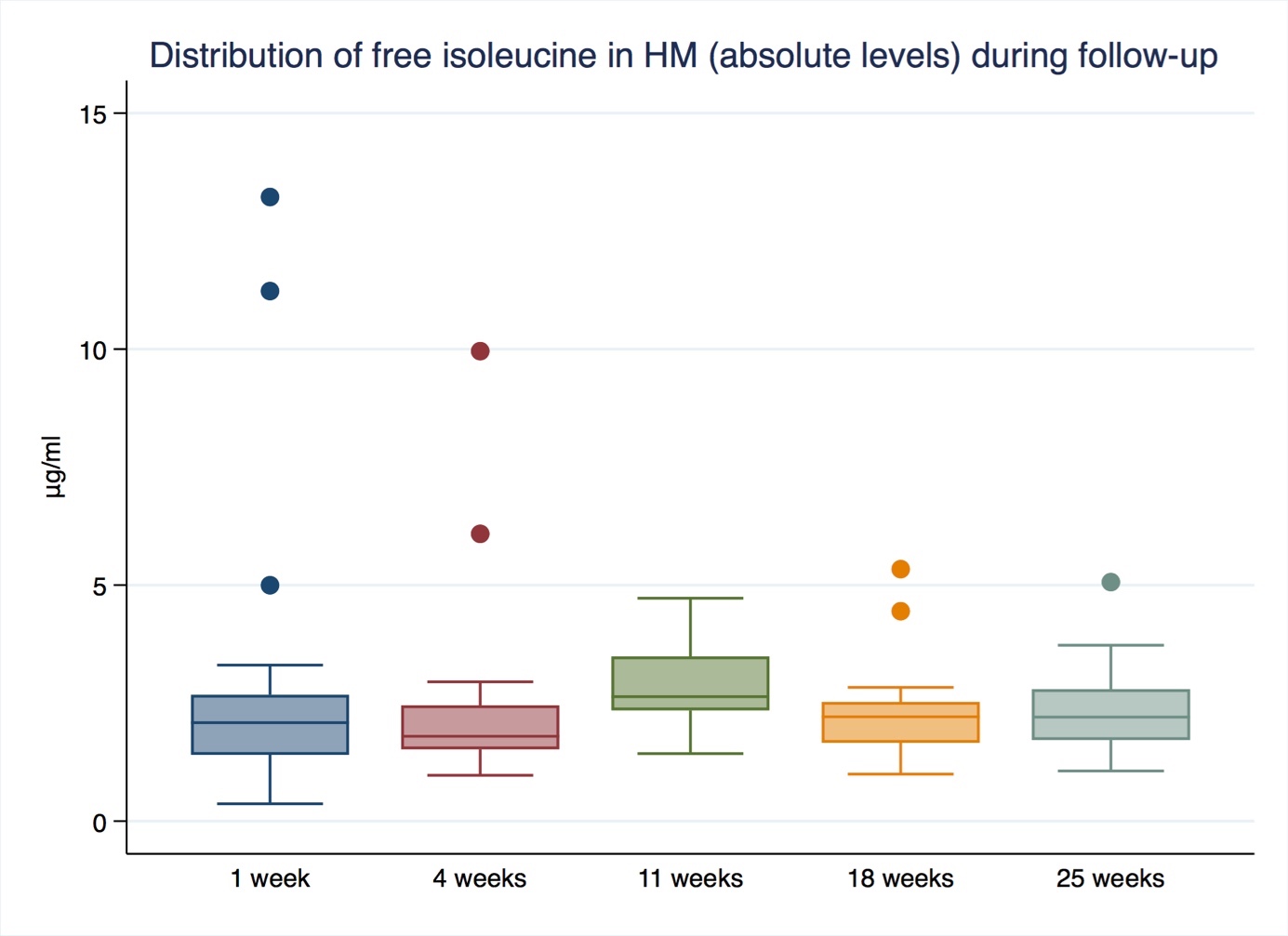

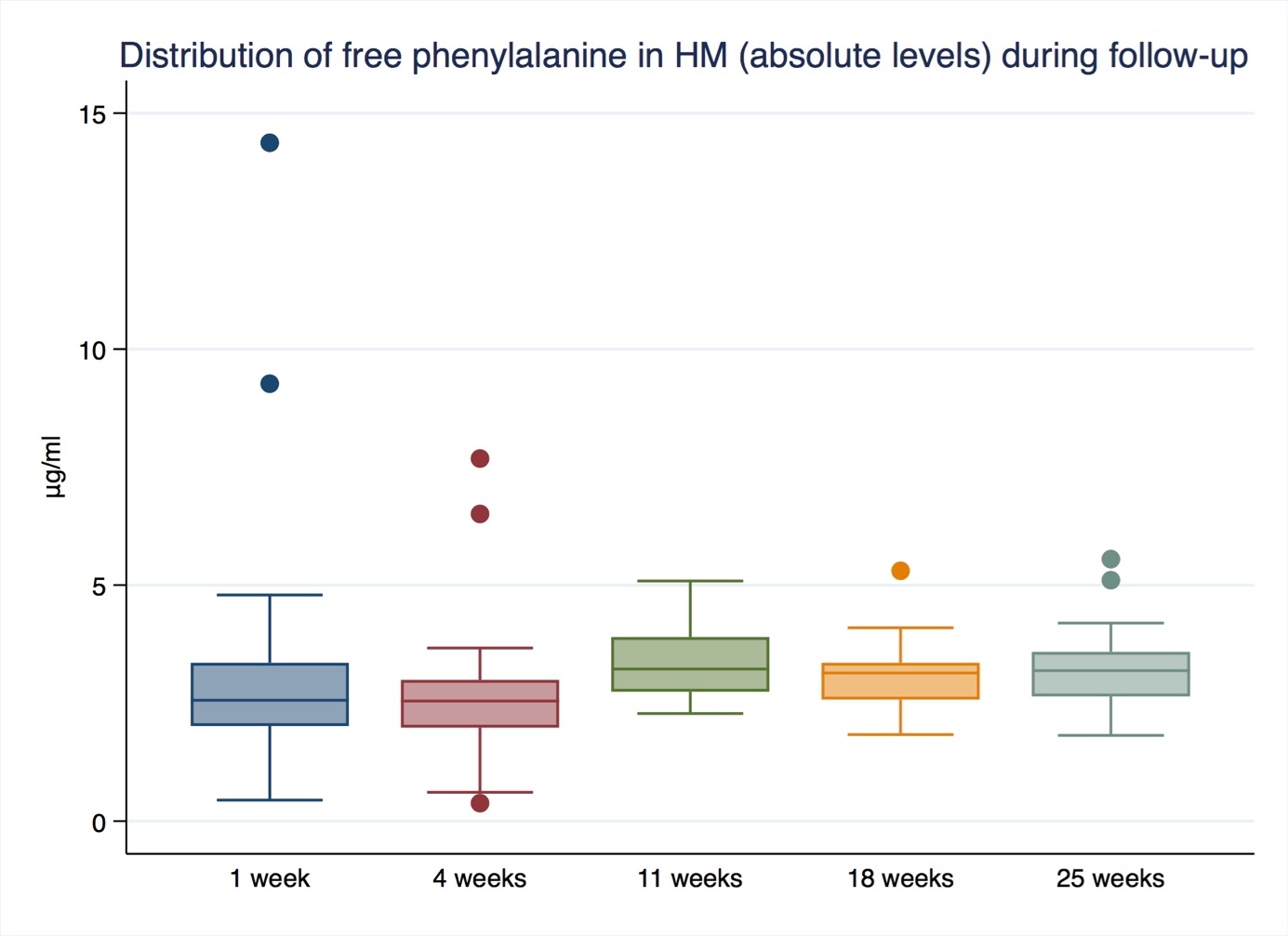

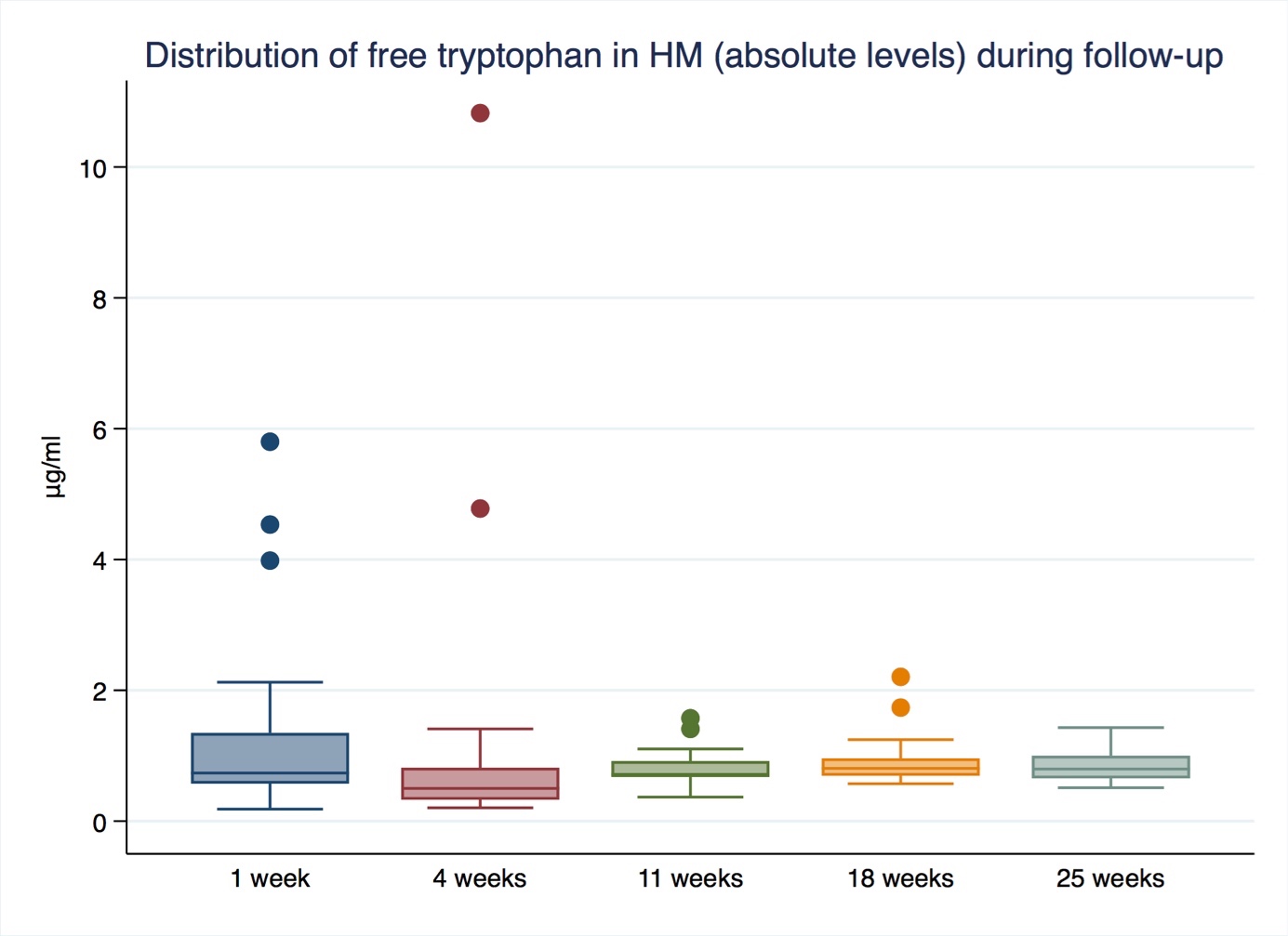

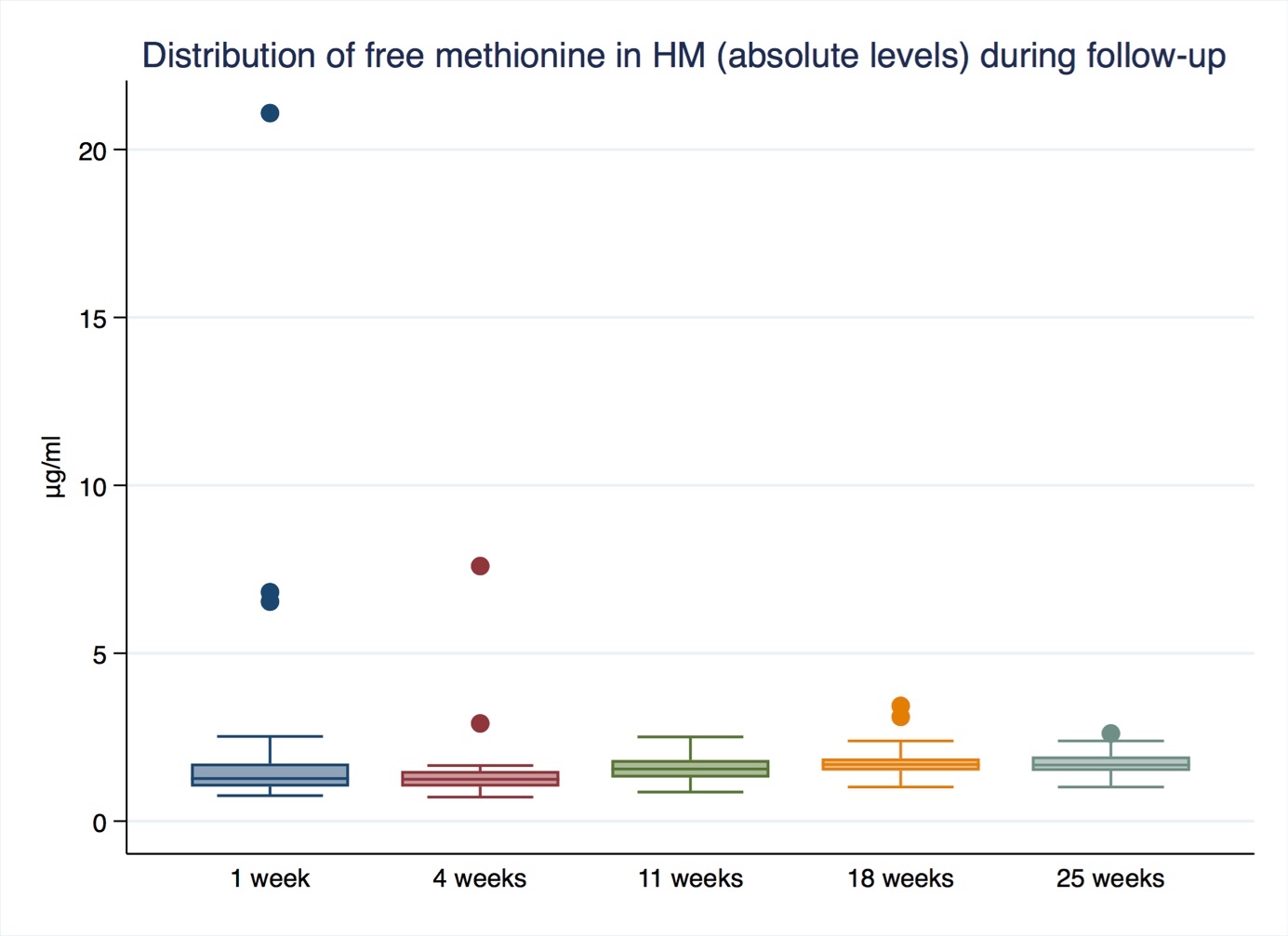

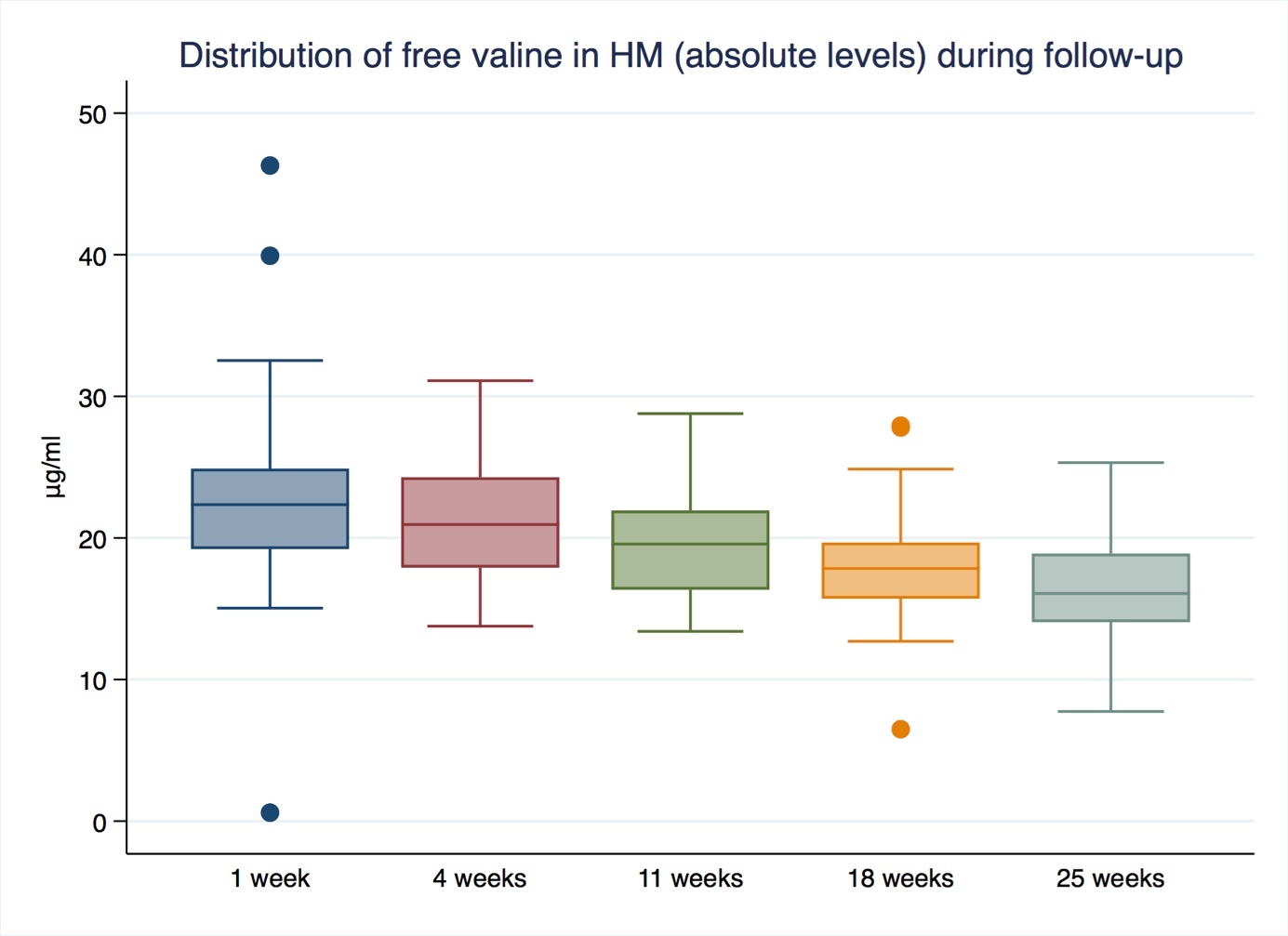

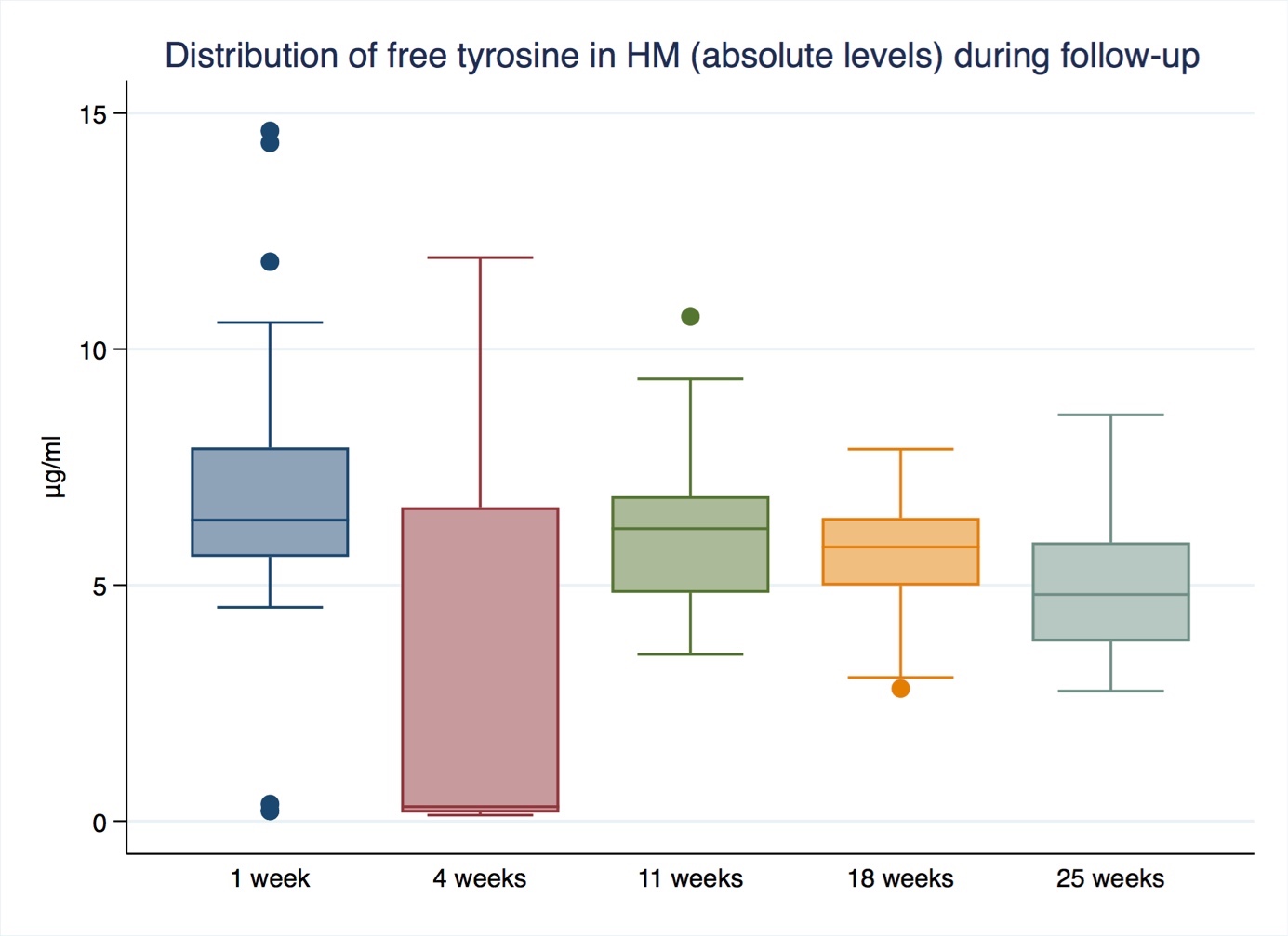

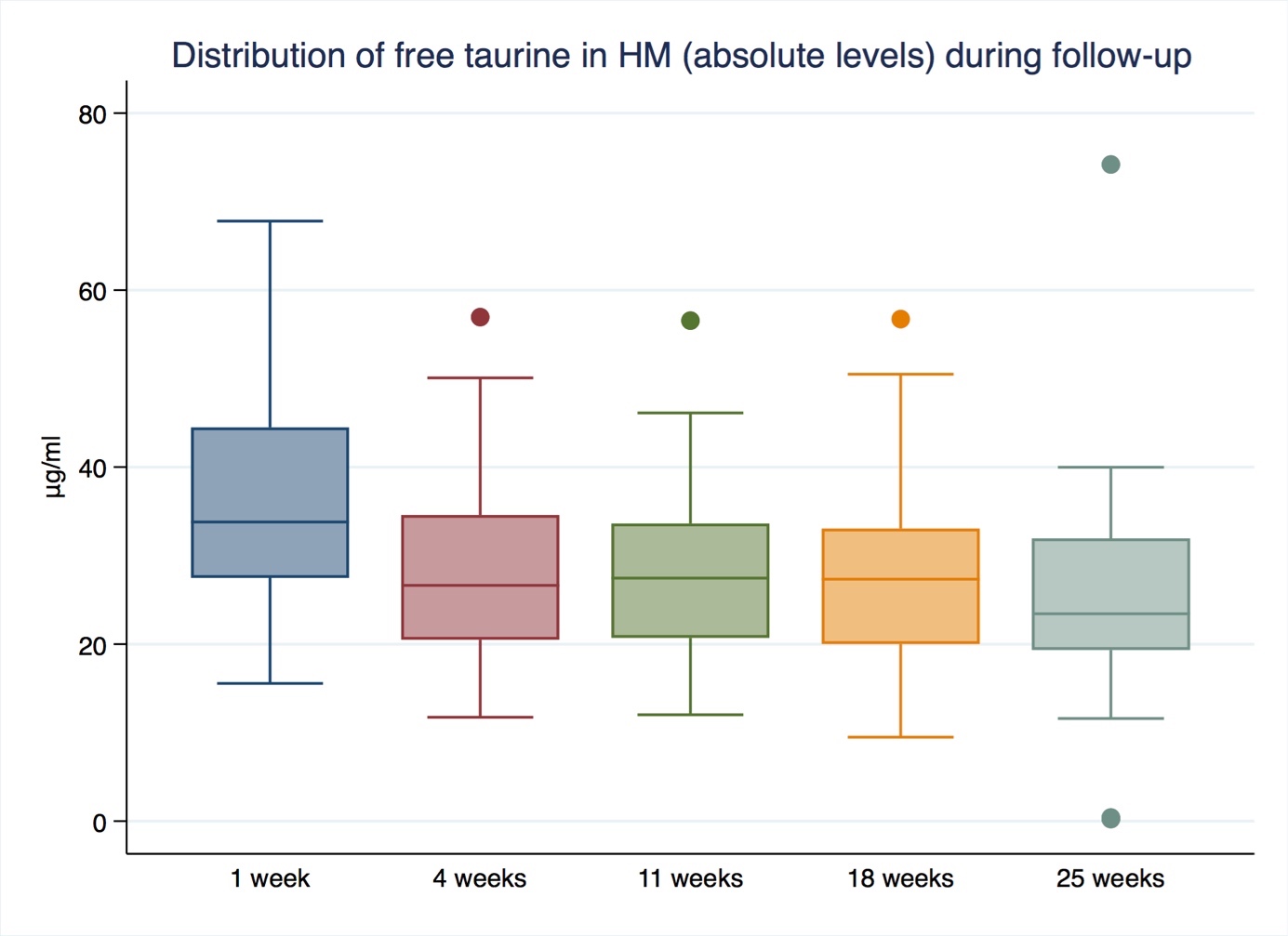

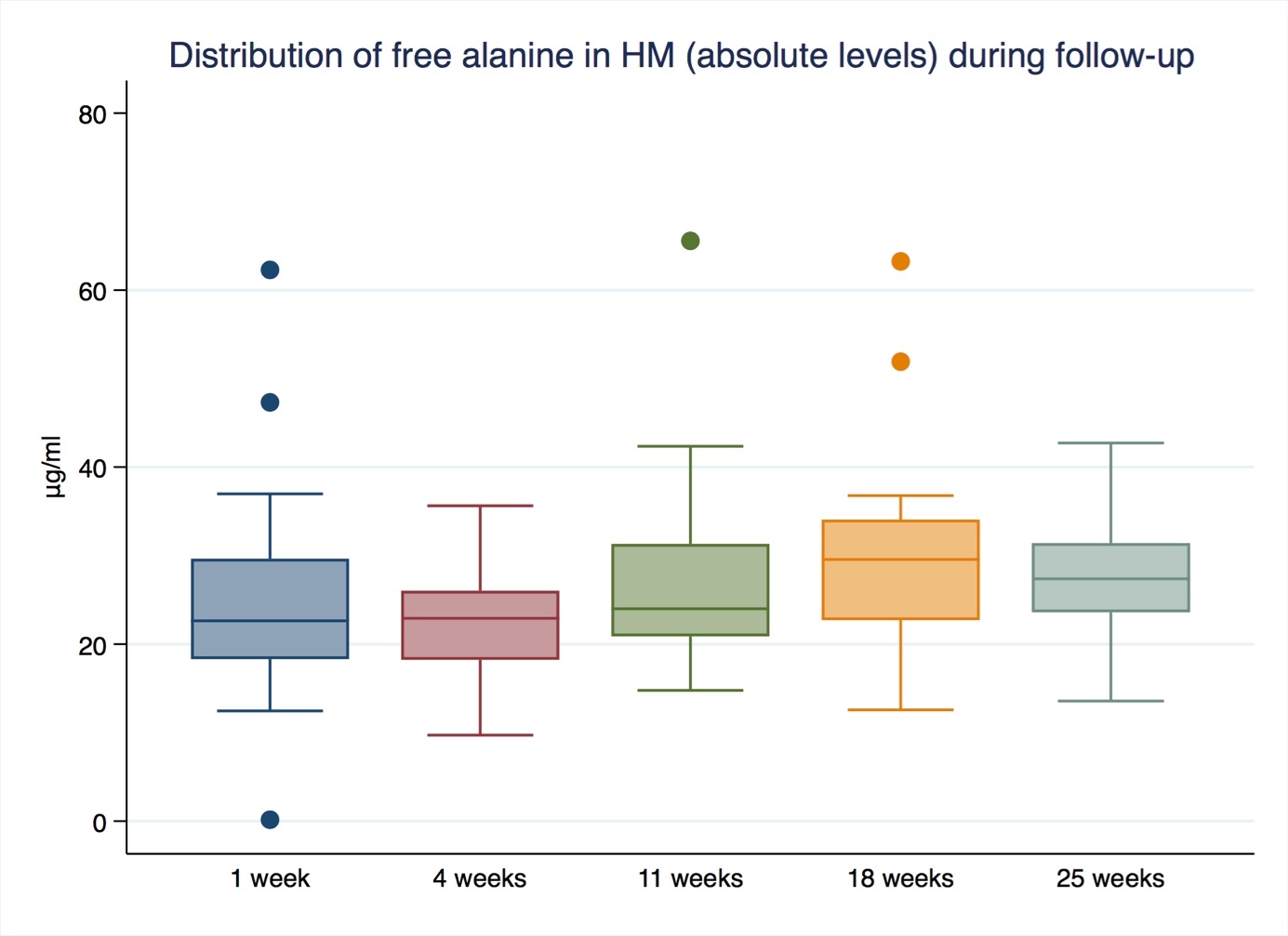

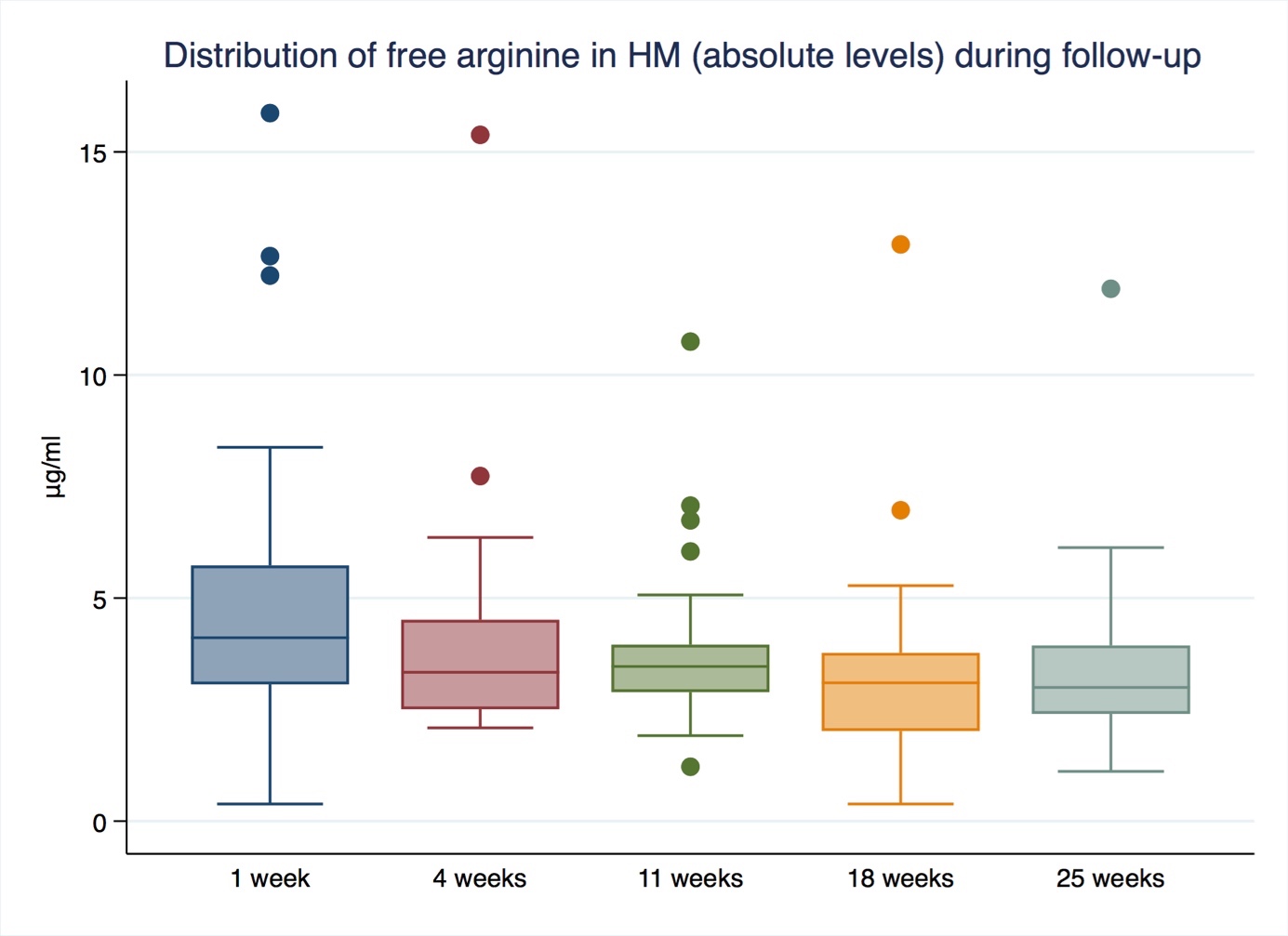

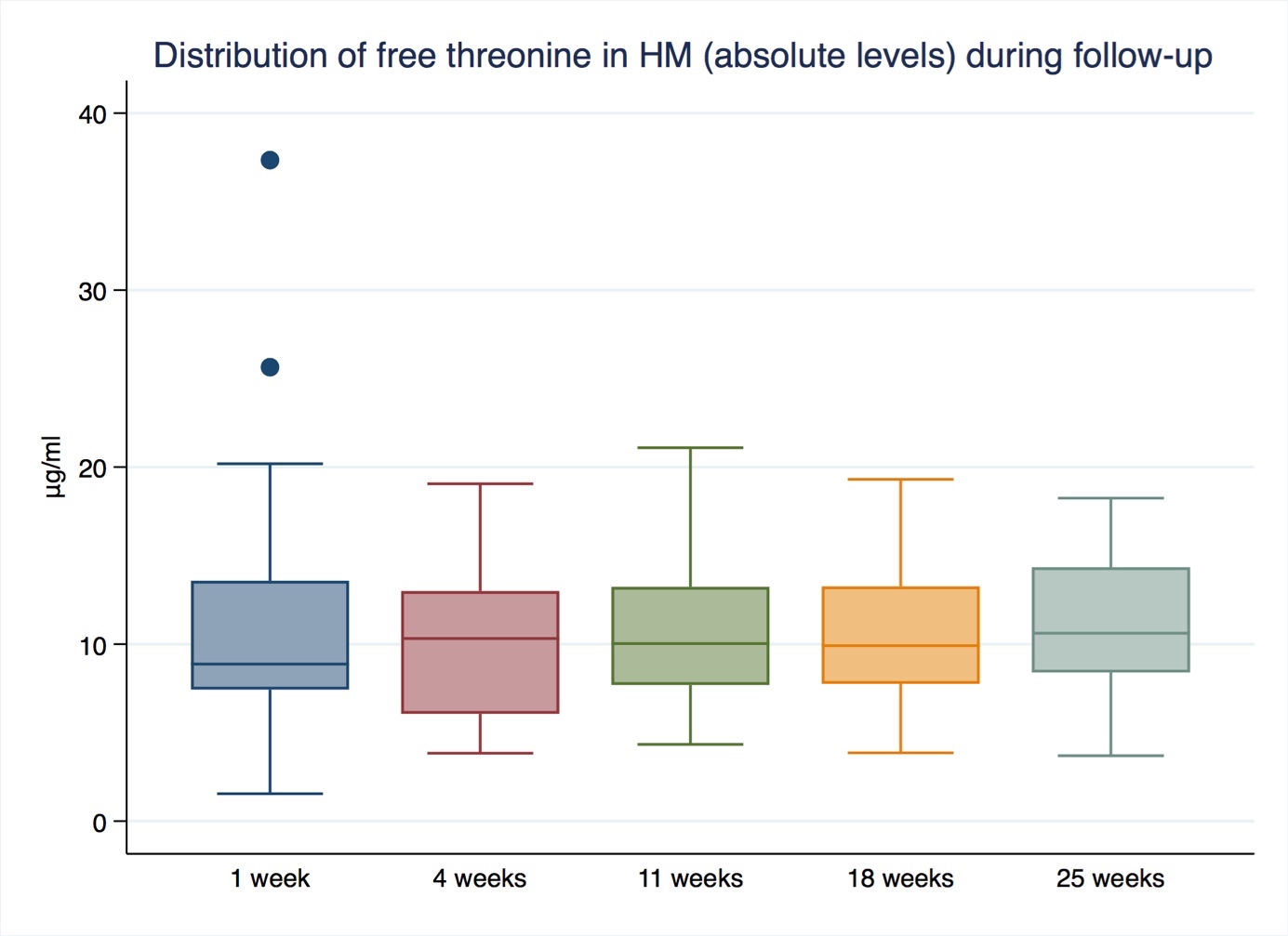

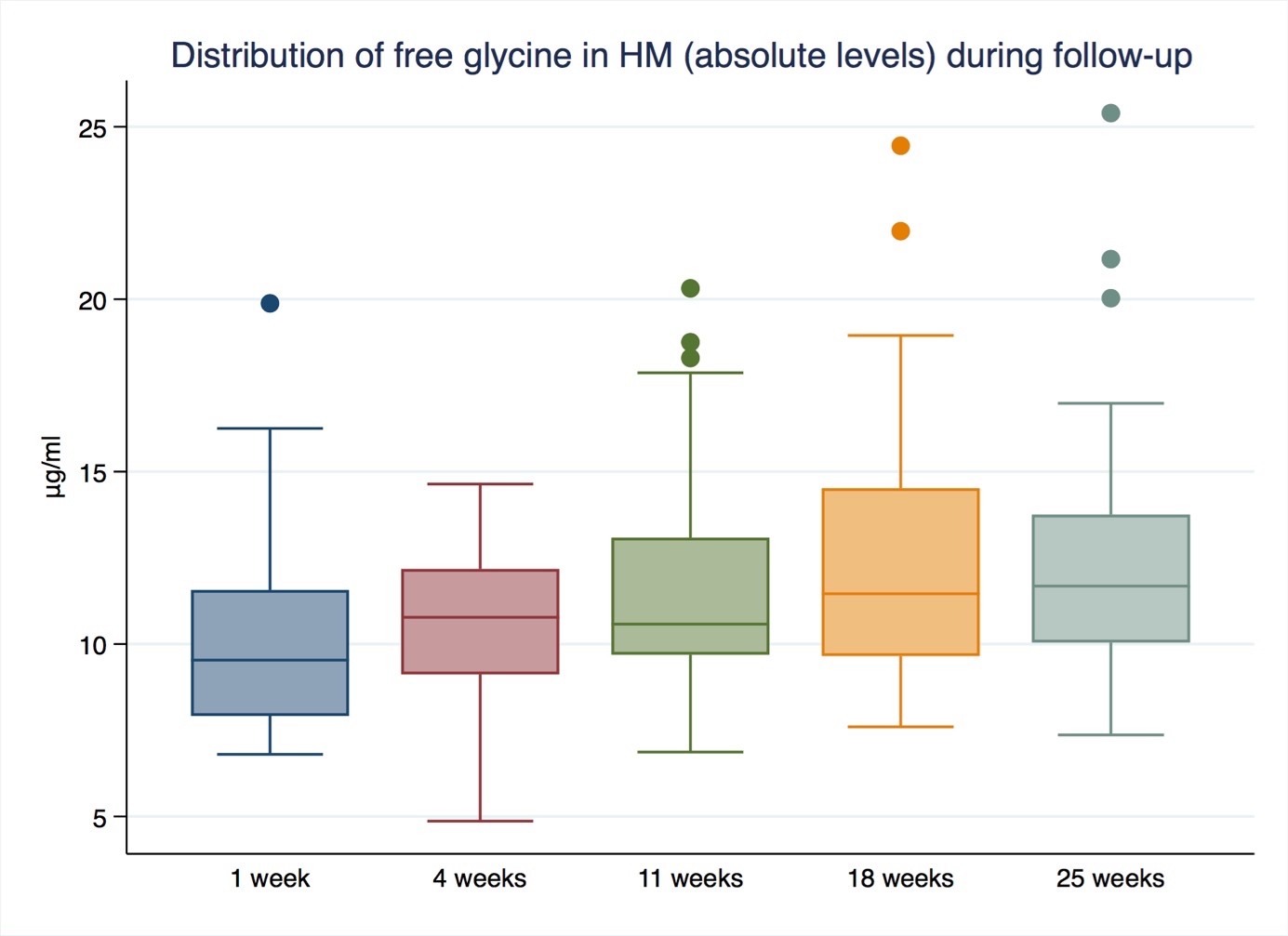

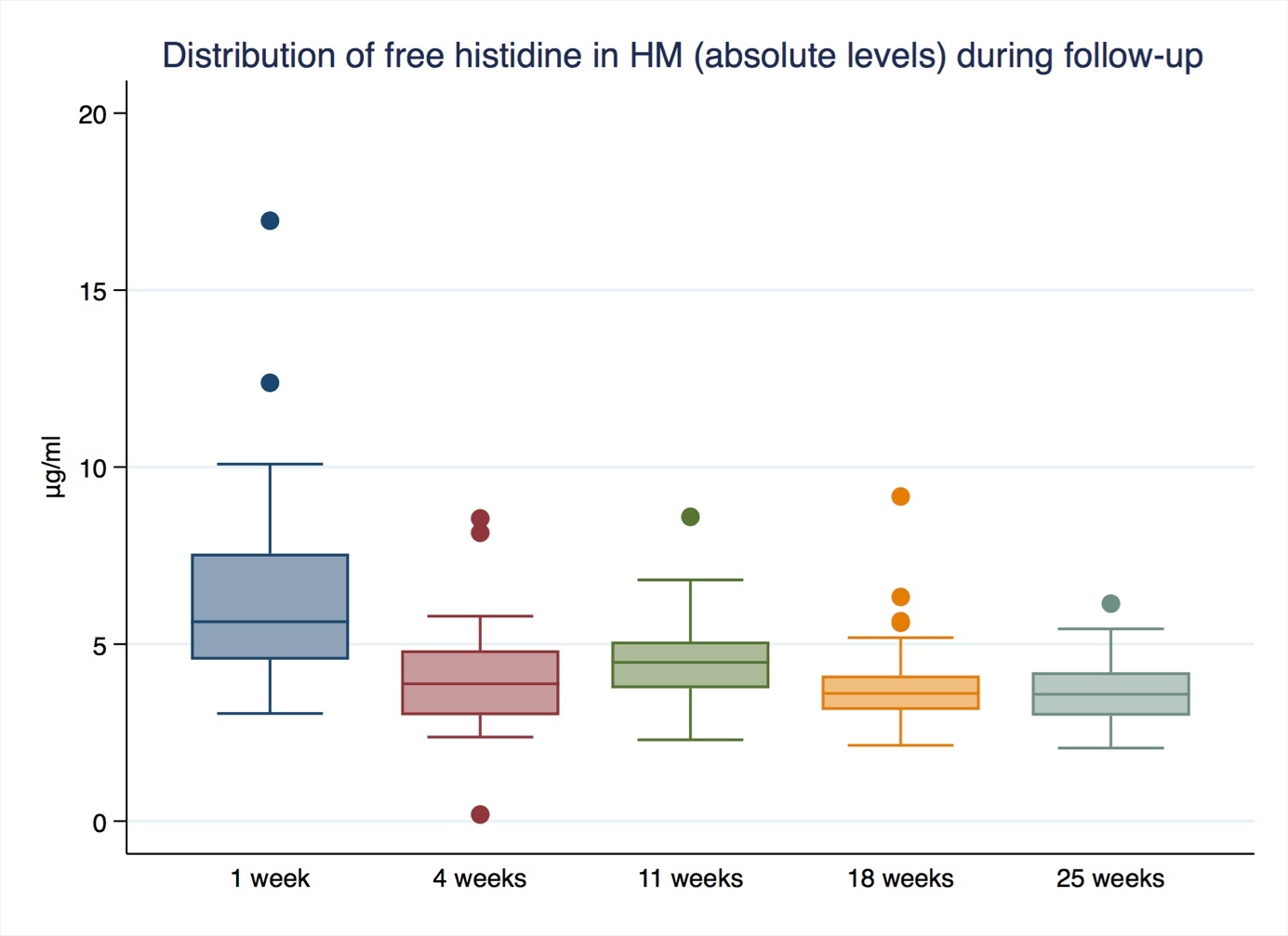

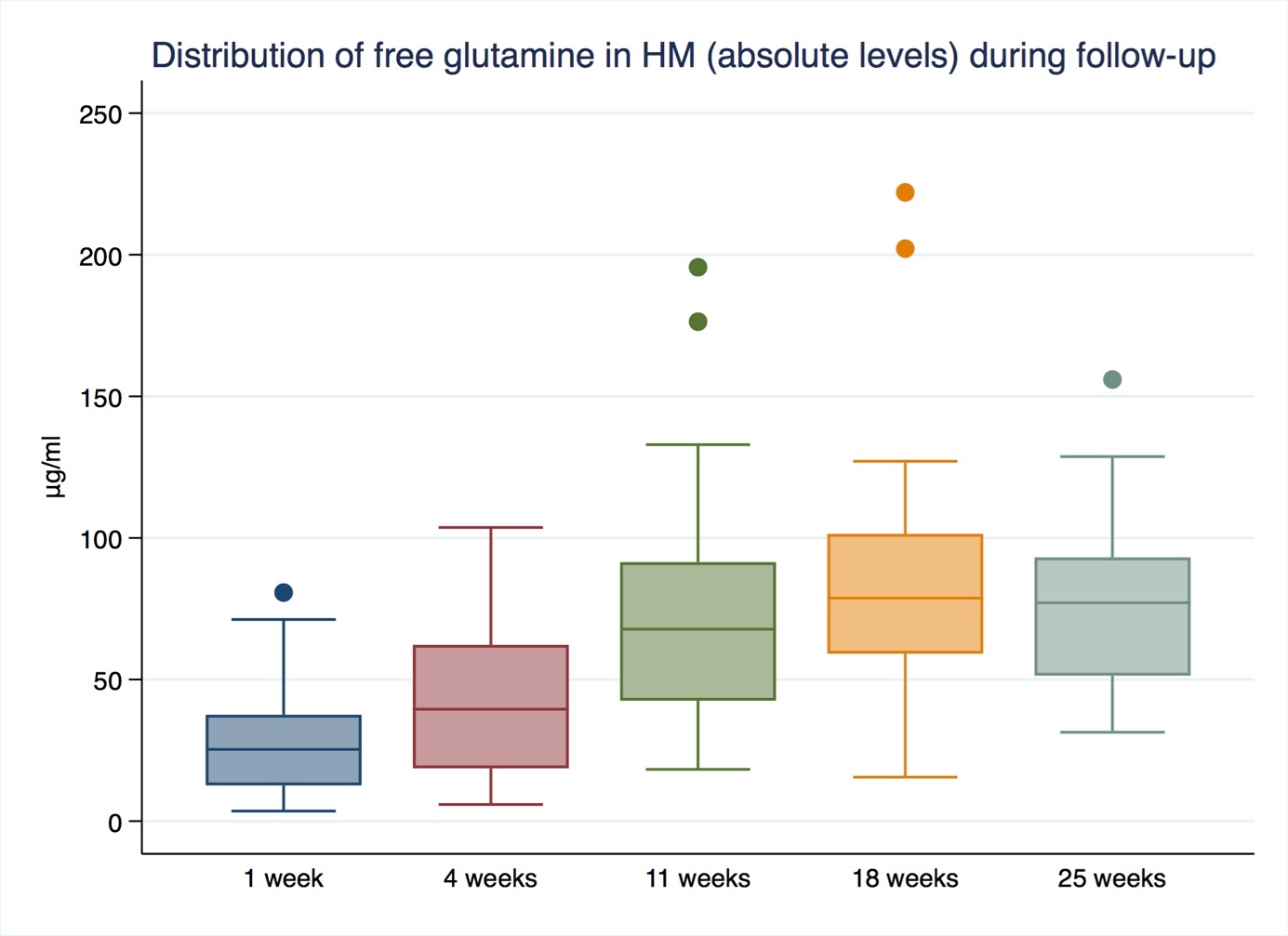

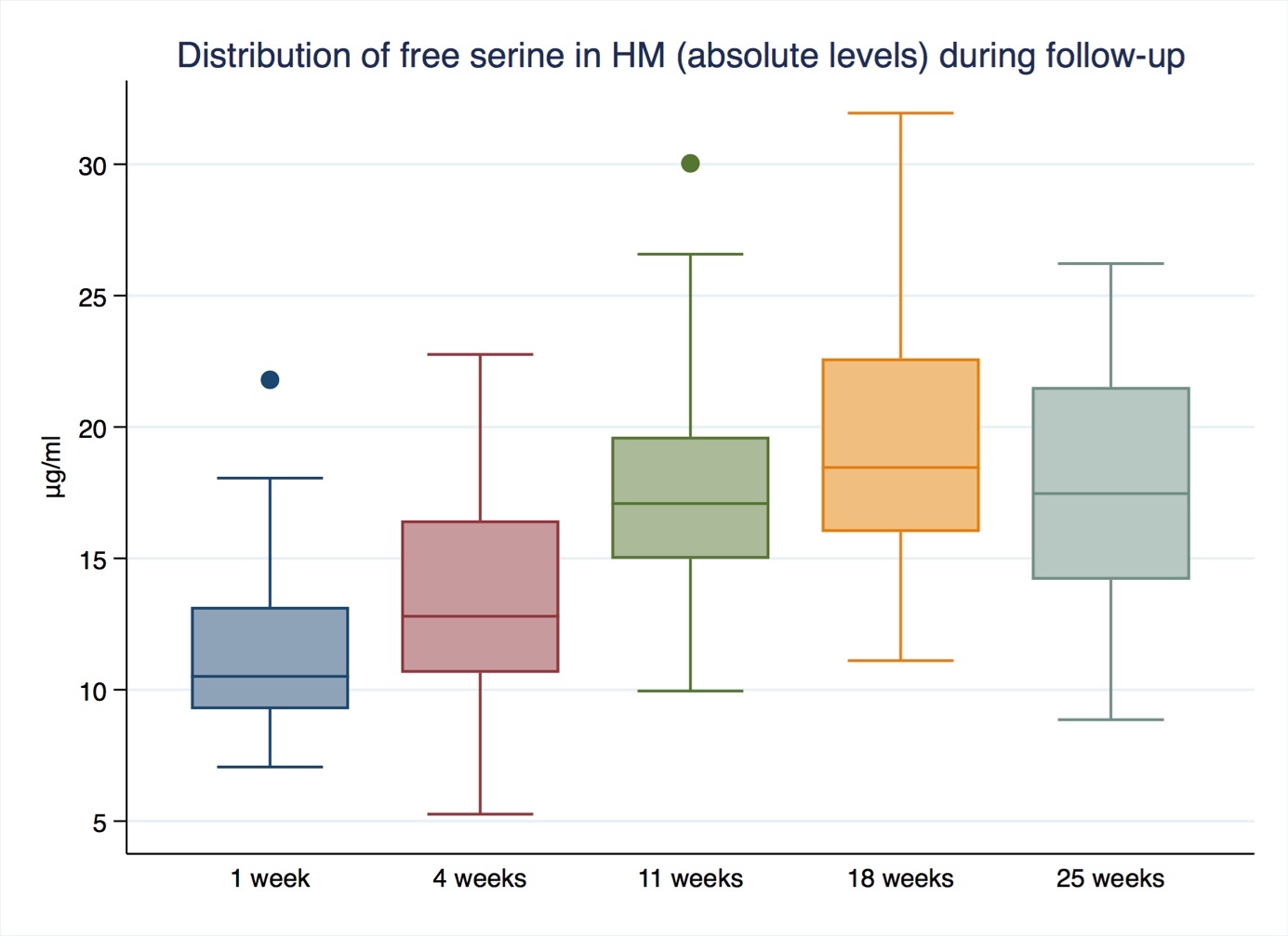

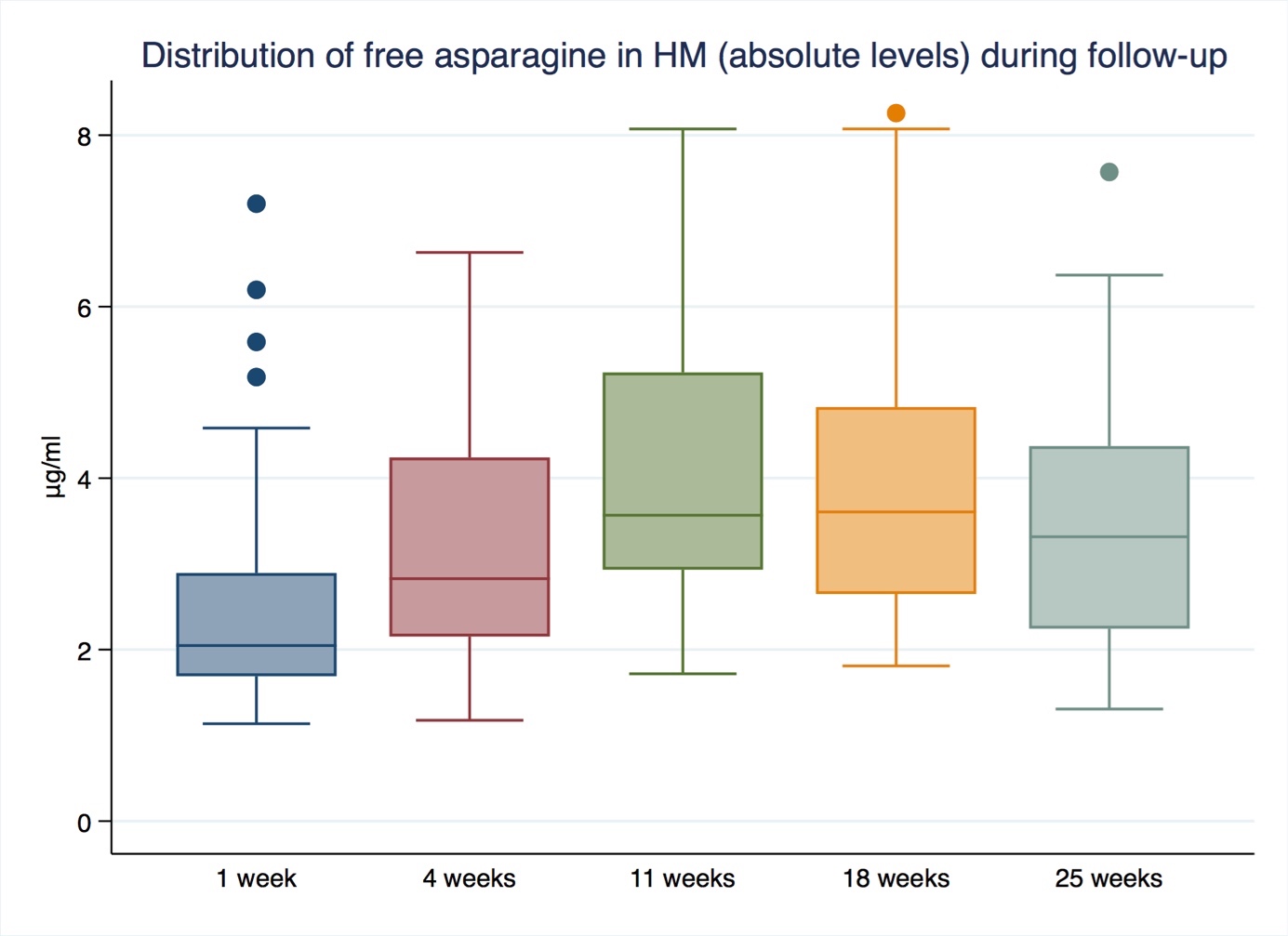

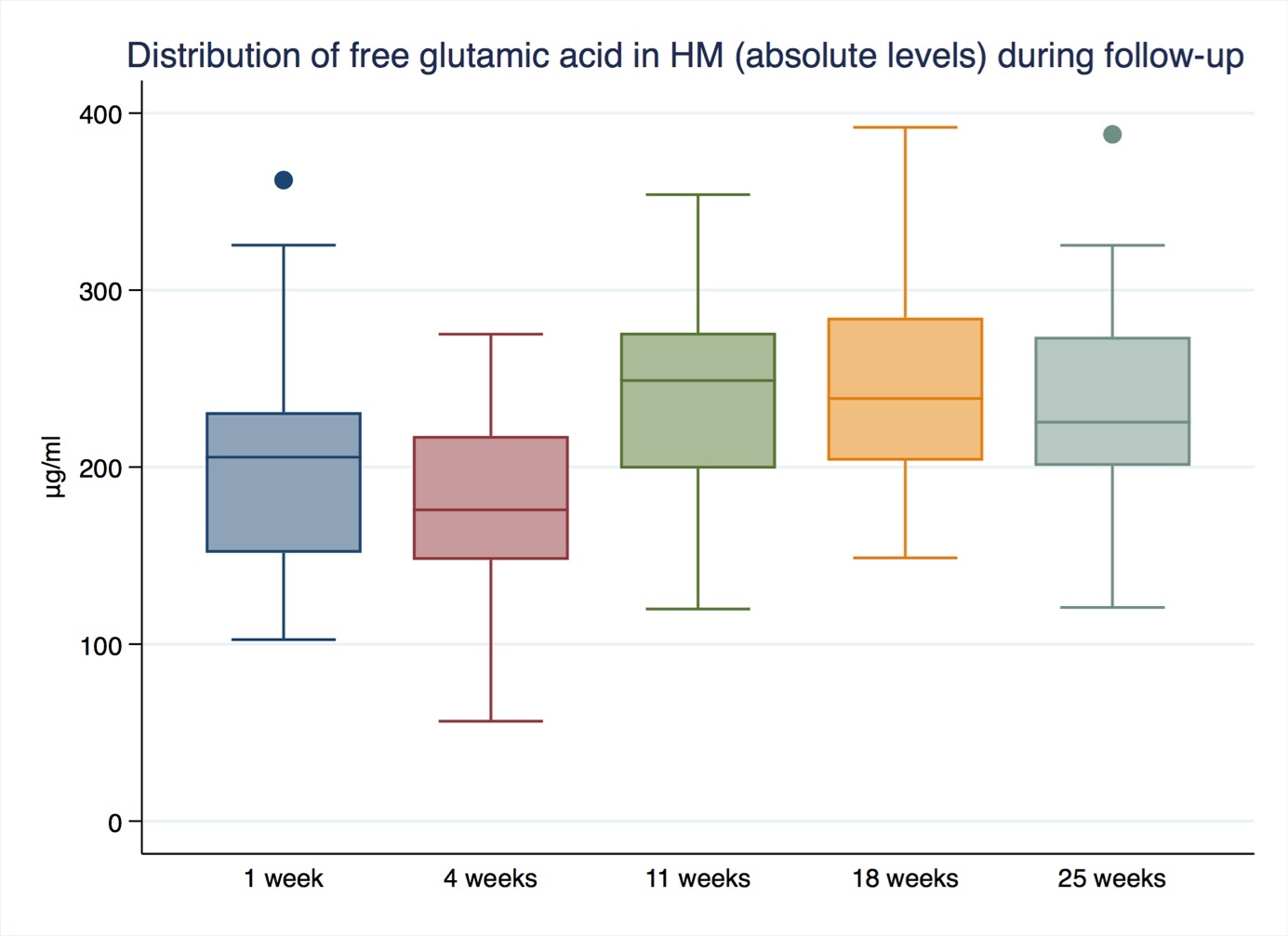

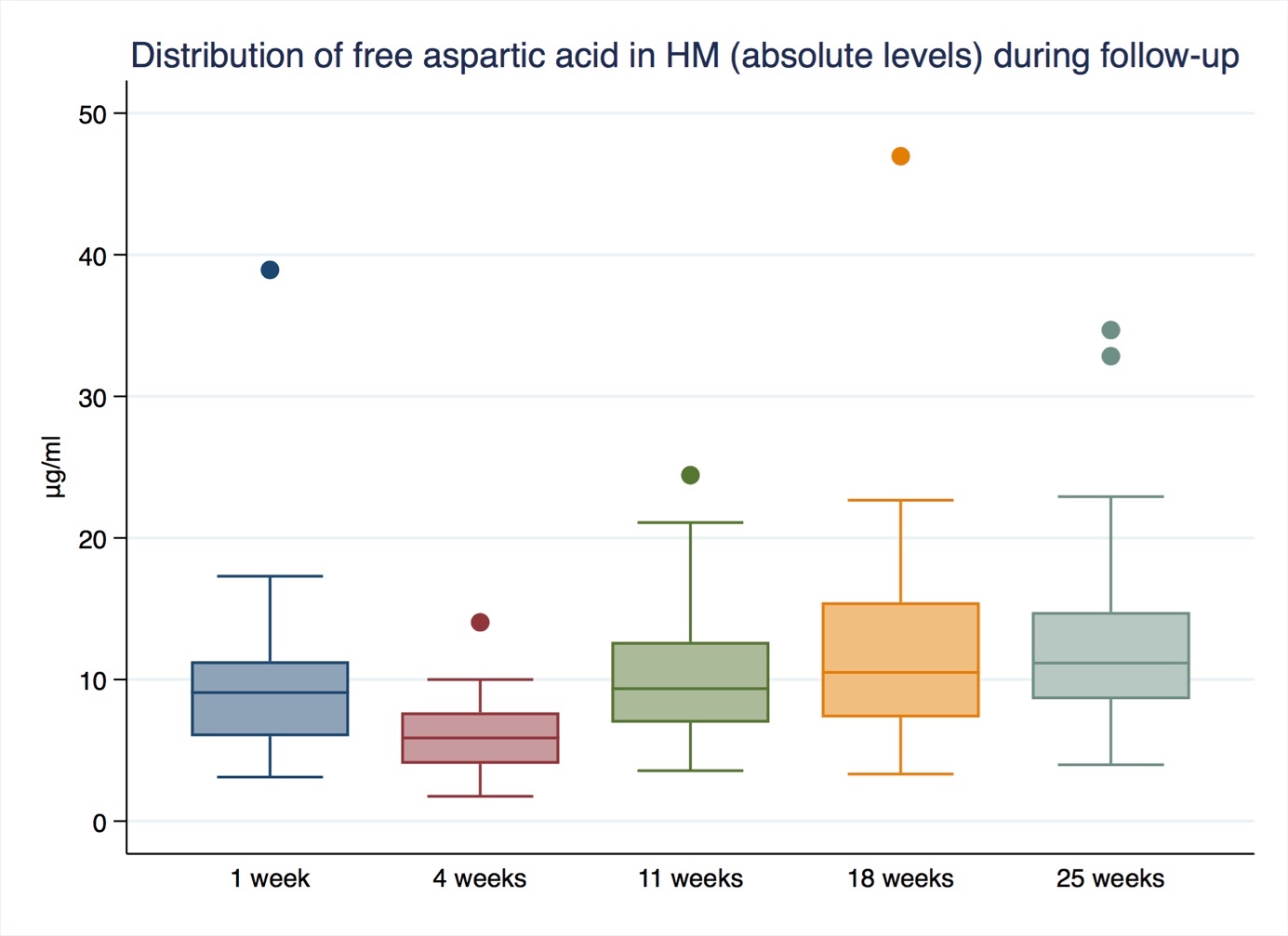


**S7. Multilevel models of free amino acids**

| **Free amino acids** | Multivariate model glutamic acid (µg/ml) | *P* value | Multivariate model glutamine (µg/ml) | *P* value | Multivariate model serine (µg/ml) | *P* value | Multivariate model tyrosine (µg/ml) | *P* value | Multivariate model of the sum of free amino acids (µg/ml) | *P* value |
| --- | --- | --- | --- | --- | --- | --- | --- | --- | --- | --- |
| **Maternal diet (food-frequency questionnaire)** |  |  |  |  |  |  |  |  |  |  |
| Grains, white roots and tubers |  |  |  |  |  |  |  |  | 53.06 (10.30; 95.83) | 0.02 |
| Pulses: beans, peas and lentils |  |  |  |  |  |  |  |  | 116.68 (7.80; 225.56) | 0.04 |
| Nuts and seeds |  |  |  |  |  |  |  |  |  |  |
| Milk and milk products |  |  |  |  |  |  |  |  |  |  |
| Meat, poultry, and fish |  |  |  |  |  |  |  |  |  |  |
| Eggs |  |  |  |  |  |  |  |  |  |  |
| Dark green leafy vegetables |  |  |  |  |  |  |  |  |  |  |
| Other vitamin A rich fruits and vegetables |  |  |  |  |  |  |  |  |  |  |
| Other vegetables |  |  |  |  |  |  |  |  |  |  |
| Other fruits |  |  |  |  |  |  |  |  |  |  |
| Insects, small rodents and other small animals |  |  |  |  |  |  |  |  |  |  |
| Red palm oil | -69.93 (-136.46; -3.41) | 0.04 |  |  |  |  |  |  |  |  |
| Other oils and fats |  |  |  |  |  |  |  |  |  |  |
| Fish | -19.94 (-38.06; -1.83) | 0.03 |  |  |  |  |  |  |  |  |
| **Food security during follow-up** |  |  |  |  |  |  |  |  |  |  |
| Household hunger scale index |  |  |  |  |  |  |  |  |  |  |
| Little to no hunger in the household |  |  |  |  |  |  | reference |  | reference |  |
| Moderate hunger in the household |  |  |  |  |  |  | -1.83 (-3.39; -0.26) | 0.02 | -62.69 (-122.47; -2.91) | 0.04 |
| Severe hunger in the household |  |  |  |  |  |  | -1.51 (-6.24; 3.21) | 0.53 | 31.97 (-146.73; 210.67) | 0.73 |
| Undernourished mother at delivery | 30.14 (10.33; 49.96) | 0.003 |  |  |  |  |  |  |  |  |
| Female infant |  |  |  |  |  |  |  |  |  |  |
| **Follow-up visits** |  |  |  |  |  |  |  |  |  |  |
| 1st week | reference |  |  |  | reference |  | reference |  | reference |  |
| 4 weeks | -23.77 (-50.60; 3.06) | 0.08 | 14.50 (-1.83; 30.82) | 0.08 | 1.98 (-0.005; 3.97) | 0.05 | -2.84 (-4.07; -1.61) | <0.001 | -57.58 (-107.88; -7.28) | 0.03 |
| 11 weeks | 50.23 (26.50; 73.96) | <0.001 | 49.72 (34.76; 64.69) | <0.001 | 6.50 (4.68; 8.32) | <0.001 | -1.13 (-2.26; -0.01) | 0.049 | 74.39 (28.39; 120.39) | 0.002 |
| 18 weeks | 42.87 (15.18; 70.56) | 0.002 | 63.47 (46.06; 80.89) | <0.001 | 6.97 (4.85; 9.09) | <0.001 | -2.17 (-3.52; -0.83) | 0.002 | 91.77 (36.92; 146.61) | 0.001 |
| 25 weeks | 52.67 (24.43; 80.91) | <0.001 | 54.62 (36.65; 72.59) | <0.001 | 6.39 (4.20; 8.58) | <0.001 | -2.33 (-3.76; -0.90) | 0.001 | 57.95 (0.30; 115.61) | 0.049 |
| **Milk-type group** |  |  |  |  |  |  |  |  |  |  |
| Milk-type group I | 20.55 (-2.12; 43.21) | 0.08 | -0.18 (-21.17; 20.82) | 0.99 | -0.89 (-3.53; 1.75) | 0.51 | -1.55 (-2.84; -0.26) | 0.02 | 10.95 (-34.37; 56.27) | 0.64 |
| Milk-type group II | -25.44 (-51.19; 0.30) | 0.05 | -25.39 (-48.51; -2.27) | 0.03 | -3.81 (-6.71; -0.91) | 0.01 | -1.50 (-2.98; -0.03) | 0.046 | -83.33 (-135.45; -31.22) | 0.002 |
| Milk-type group III | reference |  | reference |  | reference |  | reference |  | reference |  |
| Milk-type group IV | 2.05 (-30.83; 34.92) | 0.90 | -18.30 (-47.80; 11.21) | 0.22 | -2.96 (-6.66; 0.75) | 0.12 | -2.26 (-4.06; -0.46) | 0.01 | -21.21 (-84.80; 42.38) | 0.51 |

**Appendix A**

Relative values of HMOs differed significantly between HM-types, except for LNnT. Precisely, adjusted on lactation period, and compared to HM-type III (the most prevalent), 2’FL levels were lower in HM-type I, (aß-coef = -0.2, *P* value < 0.001); relative values of 3-FL were higher in HM-type I and HM-type II (aß-coef = 0.1, *P* value < 0.001; and aß-coef = 0.3, *P* value < 0.001, respectively); 3'SL relative values were higher in HM-type II and HM-type IV (aß-coef = 0.01, *P* value = 0.03; and aß-coef = 0.04, *P* value < 0.001, respectively); relative 4'-GL was higher in HM-type IV (aß-coef = 0.0001, *P* value = 0.02); 6'-GL was higher in HM-type II and HM-type IV (aß-coef = 0.002, *P* value = 0.02; and aß-coef = 0.002, *P* value = 0.004, respectively); relative DFL was higher in HM-type I (aß-coef = 0.2, *P* value < 0.001); relative 6'-SL was higher in HM-type IV (aß-coef = 0.03, *P* value < 0.001); LNT was higher in HM-type II and HM-type IV (aß-coef = 0.1, *P* value = 0.02; and aß-coef = 0.5, *P* value < 0.001, respectively); relative LNFP-III was higher in HM-type IV (aß-coef = 0.03, *P* value = 0.007); relative LNFP-V was higher in HM-type I, HM-type II and HM-type IV (aß-coef = 0.004, *P* value = 0.002; aß-coef = 0.02, *P* value < 0.001; and aß-coef = 0.01, *P* value = 0.001, respectively); and relative LNDFHII + LNnDFHII were higher in HM-type I and HM-type II (aß-coef = 0.004, *P* value = 0.045; and aß-coef = 0.03, *P* value < 0.001, respectively).

**Appendix B**

Relative levels of total amino acids also differed significantly among HM types. Precisely, adjusted on lactation period and compared to HM-type III, the proportion of serine (median = 5.7%, IQR = 5.6%, 5.9%, aß-coef = -0.002, *P* value = 0.007), glycine (median = 2.7%, IQR = 2.7%, 2.9%, aß-coef = -0.002, *P* value = 0.02), threonine (median = 5.2%, IQR = 5.1%, 5.4%, aß-coef = -0.002, *P* value = 0.006), alanine (median = 4.6%, IQR = 4.5%, 4.7%, aß-coef = -0.002, *P* value = 0.002), and phenylalanine (median = 4.4%, IQR = 4.3%, 4.6%, aß-coef = -0.01, *P* value = 0.02) were significantly lower in HM samples of HM-type I. On the contrary, the percentage of valine (median = 6.9%, IQR = 6.8%, 7.1%, aß-coef = 0.002, *P* value = 0.001), leucine (median = 11.7%, IQR = 11.4%, 11.8%, aß-coef = 0.002, *P* value = 0.02), and methionine (median = 1.6%, IQR = 1.6%, 1.7%, aß-coef = 0.001, *P* value = 0.01) were significantly higher in HM-type II, compared to HM-type III, and adjusted on lactation period. The percentage of methionine was also significantly higher in HM-type IV samples (median = 11.6%, IQR = 11.3%, 11.6%, aß-coef = 0.001, *P* value = 0.01), compared to HM-type III.

**Appendix C**

Relative levels of free glutamic acid were significantly higher in HM-type I, compared to HM-type III, in multivariate analysis adjusted on lactation period (median = 52.0%, IQR = 49.7%, 56.4%, aß-coef = 0.04, *P* value = 0.004). On the contrary, samples of HM-type I displayed significantly lower relative levels of free glycine, compared to samples of HM-type III (median = 2.3%, IQR = 2.0%, 2.8%, aß-coef = -0.004, *P* value = 0.02). In parallel, relative levels of free valine were significantly higher in HM-type II (median = 5.2%, IQR = 4.4%, 6.8%, aß-coef = 0.12, *P* value = 0.01), whereas relative levels of glutamine were significantly lower in HM-type II (median = 11.0%, IQR = 6.7%, 13.6%, aß-coef = -0.04, *P* value = 0.03.
